# An Australian and New Zealand clinical practice guideline for the physiotherapy management of people with spinal cord injuries

**DOI:** 10.1038/s41393-025-01088-8

**Published:** 2025-08-12

**Authors:** Joanne V. Glinsky, Lisa A. Harvey, Keira E. Tranter, Leanne Rees, Mark McDonald, Brooke Wadsworth, Emilie Gollan, Verna Stavric, Jo Nunnerley, Jennifer Dunn, Deanne Wilson, Australian and New Zealand Clinical Practice Guidelines Consortium, Joanne V. Glinsky, Lisa A. Harvey, Keira E. Tranter, Leanne Rees, Mark McDonald, Brooke Wadsworth, Emilie Gollan, Verna Stavric, Jo Nunnerley, Jennifer Dunn, Deanne Wilson, Jacqui Agostinello, Marsha Ben, David J. Berlowitz, Lynn Blecher, Adrian Byak, Elizabeth Bye, Sara Calthorpe, Jackie Chu, Lydia W. Chen, Leisl Davis, Sophia Denis, Camila Quel De Oliveira, Mario D’Cruz, Fernanda Di Natal, Sheelagh Donohoe, Ché Fornusek, Amanda Haber, Annie Illman, Mel Kotze, Lucy Maugham, Joanna Mather, Jennie McCorkell, Janet McCarthy, Tony McDonald, Anthony Nakhle, Helen Patterson, Jai Peach, Donna Rainey, Jason Redhead, Jacqueline Ross, Jonathan Tang, Maree Walters, Jacqui White

**Affiliations:** 1 John Walsh Centre for Rehabilitation Research, Kolling Institute, Faculty of Medicine and Health, University of Sydney, Sydney, NSW Australia; 2 Discipline of Physiotherapy, University of Sydney, Sydney, NSW Australia; 3 Victorian Spinal Cord Service, Austin Health, Melbourne, VIC Australia; 4 Metro North Health, Brisbane, QLD Australia; 5 Princess Alexandra Hospital, Metro South Health, Brisbane, QLD Australia; 6 Auckland University of Technology, Auckland, New Zealand; 7 Orthopaedic Surgery and Musculoskeletal Medicine, University of Otago, Christchurch, New Zealand; 8 Spinal Outreach Team, South Australian Spinal Cord Injury Service, Adelaide, SA Australia; 9 Royal Rehab, Sydney, NSW Australia; 10 University of Melbourne, Melbourne, VIC Australia; 11 Austin Health, Melbourne, VIC Australia; 12 Prince of Wales Hospital, South Eastern Sydney Local Health District, Sydney, NSW Australia; 13 Optimise Your Level Physiotherapy, Sydney, NSW Australia; 14 Neuroscience Research Australia and University of NSW, Sydney, NSW Australia; 15 The Alfred, Melbourne, VIC Australia; 16 Royal North Shore Hospital, Northern Sydney Local Health District, Sydney, NSW Australia; 17 University of Technology, Sydney, NSW Australia; 18 Australian Quadriplegic Association, Melbourne, VIC Australia; 19 University of Sydney, Sydney, NSW Australia; 20 Northern Beaches Rehabilitation, Sydney, NSW Australia; 21 South Australian Spinal Cord Injury Service, Adelaide, SA Australia; 22 Queensland Spinal Cord Injuries Service, Brisbane, QLD Australia; 23 Middlemore hospital, Manukau District, Health New Zealand, Auckland, New Zealand; 24 MyTurn Rehabilitation, Brisbane, QLD Australia; 25 Royal Adelaide Hospital, Adelaide, SA Australia; 26 Brisbane, QLD Australia; 27 Granville Family Medical Centre, Sydney, NSW Australia; 28 Auckland Spinal Rehabilitation Unit, Counties Manukau, Health New Zealand, Auckland, New Zealand

**Keywords:** Health policy, Neurological disorders

## Abstract

**Study design:**

Development of a Clinical Practice Guideline (CPG).

**Objective:**

To develop a CPG for the physiotherapy management of people with Spinal Cord Injuries (SCI).

**Setting:**

Australia and New Zealand.

**Methods:**

Systematic reviews of randomised controlled trials (RCTs) of physiotherapy interventions for adults with SCI were conducted to address over 100 clinical questions. Questions were decided a priori and written in PICO format (Participant, Intervention, Comparison and Outcome). Meta-analyses were conducted across trials that made similar comparisons. A Grading of Recommendations Assessment, Development and Evaluation (GRADE) approach was used to assess evidence certainty and formulate recommendations. A Guideline panel made evidence recommendations and consensus-based opinion statements based on a standardised process that included voting.

**Results:**

Seventy-six RCTs met the inclusion criteria for the systematic reviews. These RCTs informed 20 meta-analyses that were used in the development of the CPG. More than one hundred evidence recommendations and consensus-based opinion statements across 13 categories of physiotherapy interventions were made by the panel.

**Conclusion:**

The Australian and New Zealand CPG for the Physiotherapy Management of people with SCI provide clear and readily accessible guidance to physiotherapists based on evidence and consensus of clinical experts. The Guideline is available at www.sciptguide.com.

## Introduction

High quality Clinical Practice Guidelines (CPGs) about physiotherapy management are essential to direct care for people with Spinal Cord Injuries (SCI). They provide clinicians, researchers, caregivers, and people living with SCI with recommendations based on the evidence about physiotherapy interventions. They also provide an accessible summary underpinned by methodology that has been used and adapted by many groups across many health conditions. Evidence based guidance about physiotherapy treatment is currently limited. There are guidelines that cover components of physiotherapy care such as neuropathic pain management [1], exercise prescription [2, 3], or as part of a guideline of overall SCI management (www.canscip.ca). There are also comprehensive summaries of evidence and systematic reviews that have assessed the effectiveness of different interventions (https://scireproject.com/). However, prior to the development of this guideline there was no CPG that used specific PICO (Participant, Intervention, Comparison, Outcome) questions identified by physiotherapists and people with SCI or restricted questions to interventions administered by physiotherapists. In addition, there was no CPG that used the rigorous process recommended by GRADE [4] and the Australian National Health and Medical Research Council (NHMRC) [5] to develop recommendations and consensus-based opinion statements for physiotherapy management.

The Australian and New Zealand CPG for the management of people with SCI were developed using the GRADE approach [4, 6]. Initially over 100 PICOs were identified and prioritised covering a range of commonly administered physiotherapy interventions. These PICOs were decided on by a guideline panel and informed by qualitative interviews with people with SCI and other stakeholders [7, 8]. The panel made either evidence-based recommendations or consensus-based opinion statements about the interventions captured within each PICO, using the comprehensive evidence to decision framework to inform decisions.

The recommendations and statements from the Australian and New Zealand CPG are reported within this paper. The aims of this paper are to describe the methodology used to generate our CPG and to provide an overview of the key evidence-based recommendations and consensus-based opinion statements comprising the CPG.

## Methods

A Grading of Recommendations Assessment, Development and Evaluation (GRADE) approach was used to develop recommendations [4, 6]. Guidance from the NHMRC was used to develop consensus-based opinion statements with accompanying clinical notes [5]. The process used is outlined below with a full description of the methodology detailed in Supplementary 1.

### PICO questions

Over 100 questions were decided a priori and presented in PICO format. PICO questions were based on interventions that were routine clinical practice within the Australian and New Zealand context and were written by the Guideline panel. In most PICOs the “comparison” was no intervention (or a sham intervention). However, in some of the PICOs, the “comparison” was another intervention to reflect the common decisions physiotherapists often need to make when posed with two treatment options. The panel met to discuss the drafted PICO questions which were then adopted, rejected, or changed. The PICO questions can be found in Supplementary 1. They do not cover the full scope of physiotherapy practice but do reflect the questions prioritised by the panel.

### Systematic reviews of the evidence

A systematic review was conducted on each PICO. The aim of each systematic review was to determine the effectiveness of each physiotherapy intervention compared with no intervention, a sham intervention or another physiotherapy intervention on outcomes of impairment, activity limitation or participation as defined by the PICO. The types of studies, participants, interventions, comparisons and outcomes captured are outlined below.

### Types of studies

Published randomised controlled trials (RCTs) and randomised controlled cross over trials were included. Trials with more than two parallel comparisons were included if two of the comparisons met the inclusion criteria. If trials were reported in more than one publication, then the most recent publication was used. Only trials published in English were included.

### Types of participants

Adults (>16 years) with a traumatic or non-traumatic SCI were included. Trials with a mixture of participants with different neurological conditions were only included if 80% or greater of participants within the trial had a SCI. Trials which had participants with a congenital condition involving the spinal cord such as spina bifida were excluded.

### Types of interventions and comparisons

All physiotherapy interventions identified in the list of PICOs were included (see Supplementary 1). Trials were included if they compared the interventions of interest with no intervention or a sham intervention or an alternate intervention identified in the PICO. Trials that included a co-intervention or usual care were included if the co-interventions or usual care were administered to both groups.

### Types of outcome measures

Trials were included that contained an outcome relevant to each PICO. These typically included measures of impairment, activity limitation or participation restriction.

### Search methods for identification of studies

The following electronic databases were searched to identify reports of relevant studies: Ovid MEDLINE (1946 to August 13th 2020); Ovid EMBASE (1974 to August 13th 2020); EBSCO CINAHL Plus (1937 to August 13th 2020); Physiotherapy Evidence Database (PEDro) (Searched August 13th 2020) and CENTRAL on August 13th 2020. To search Medline and Embase we used the OVID search strategy for RCTs combined with search terms for SCI. To search CINAHL we used the Cochrane search strategy for RCTs combined with search terms for SCI. To search PEDro we used category Neurotrauma combined with category RCTs. To search Central we used terms for SCI. In addition, we searched the reference lists of all identified RCTs and systematic reviews. The search strategies can be found in Supplementary 1.

### Selection of studies and collation of data

Two authors independently screened the identified titles and abstracts using the pre-defined inclusion criteria detailed above. Full texts were then retrieved to confirm eligibility. Each included study was then matched to each PICO question. Disagreements throughout the process were resolved by discussion.

Data were extracted from the studies and recorded on an excel spreadsheet. One author independently extracted descriptive data including methodology, participant characteristics and group characteristics. Two authors independently extracted data to determine mean between-group differences and 95% confidence intervals (95% CI) for continuous data. This included outcome scores and number of participants overall and in each group. Data were estimated from graphs if necessary. Mean between-group difference in post-intervention scores were prioritised. Where this was not provided, mean and standard deviation (SD) of change scores followed by mean (SD) of post-intervention scores were used. If medians and inter-quartile ranges (IQR) were provided, medians were extracted and used as means, and SDs were estimated by dividing the interquartile range by 1.35. Cross-over studies were analysed using first period data or combined data if first period were not available. RevMan 5.4.1 software was used to calculate data where necessary.

Meta-analyses were conducted across studies that made similar comparisons if there were at least two studies without excessive clinical or statistical heterogeneity. Clinical heterogeneity was assessed by examining the type of participants, type and intensity of the intervention, and other issues related to the design and conduct of the studies. Statistical heterogeneity was quantified using the I^2^ statistic where an I^2^ >75% was considered to indicate excessive heterogeneity and results were not pooled. A fixed-effects model was used to pool data if the I^2^ was less than 50%, and a random-effects model was used if the I^2^ was between 50 and 75%. If studies in a meta-analysis used the same measure and same units, effects were expressed as mean difference (MD) and 95% CI. If different measures or different units were used within a meta-analysis, effects were expressed as standardised mean difference (SMD) and 95% CI. In calculating SMD post-intervention scores were not pooled with change scores. Data were analysed using RevMan v5.4.1. No sub-group or sensitivity analyses were performed.

### Assessment of risk of bias

The risk of bias in each trial was assessed by one reviewer and checked by another reviewer using the Cochrane Risk of Bias tool (RoB 2) that categorises bias as low, high or unclear (due to a lack of information or uncertainty) for each domain [9]. The domains assessed were potential bias arising from: the randomisation process; deviations from intended interventions; missing outcome data; measurement of the outcome; selection of the reported result. The PEDro score for each study was also extracted from the PEDro website (www.pedro.org.au). An author determined the PEDro score for any study that was not on the website.

### Assessment of certainty of evidence

The evidence from each systematic review for each PICO was independently graded for certainty by two reviewers. The GRADE approach was used to define the certainty of the evidence as very low, low, moderate or high (not to be confused with the GRADE approach used to make recommendations) [4].

### The CPG development committee

A CPG Development Committee (also known as a panel) was formed to create the CPG. They represented physiotherapists, academics, consumers and other related healthcare professionals: all with either professional or lived experience of SCI (see Supplementary 1). Some panel members voted on all recommendations and others voted on just the areas they had expertise in. They were all initially trained in reading and interpreting results of RCTs and the GRADE methodology.

### Development of evidence-based recommendations

The Guideline panel used the GRADE approach for the development of recommendations (not to be confused with the GRADE approach used to assess the certainty of evidence). This approach is based on the GRADE handbook [4]. The panel made recommendations for each outcome based on a standardised process that included voting. Each recommendation required 75% agreement by the Guideline panel within three rounds of voting. Panel members needed to vote each time in one of four ways. That is, they needed to vote strongly or weakly in favour of an intervention, or strongly or weakly against an intervention. A detailed flow chart of the decision-making process can be found in Supplementary 1.

All evidence recommendations were made by initially considering the size and precision of treatment effects along with the quality of the evidence. The balance between benefits and harms, values and preferences, resource use and other relevant considerations including equity, accessibility and feasibility were then considered. These considerations were documented by two authors on the evidence to decision framework document. The direction of the recommendation was expressed using the language described by GRADE as a recommendation for an intervention, against an intervention or no recommendation. No recommendation (equivalent to neutral) was made when the panel was unable to recommend for or against the intervention based on the evidence (see Table 1).

**Table 1:** Summary of the hierarchy of the evidence recommendations.

Evidence Recommendation	Explanation
Strong evidence recommendation FOR	The guideline panel is confident that they can recommend the intervention based on the evidence. A recommendation is made that the intervention should be implemented.
Weak evidence recommendation FOR	The guideline panel is confident that they can probably recommend the intervention based on the evidence. A recommendation is made that the intervention may be implemented.
Weak evidence recommendation AGAINST	The guideline panel is confident that they probably cannot recommend the intervention based on the evidence. A recommendation is made that the intervention should not be implemented.
Strong evidence recommendation AGAINST	The guideline panel is confident that they cannot recommend the intervention based on the evidence. A recommendation is made that the intervention should definitely not be implemented.
No recommendation	The guideline panel is unable to recommend for or against the intervention based on the evidence. A consensus-based opinion statement is made.

Summary is based on the Grading of Recommendations Assessment, Development and Evaluation (GRADE) approach. Source: www.sciptguide.com.

Where no recommendation could be made or no evidence existed on which to base a recommendation, the Guideline panel voted on a consensus-based opinion statement.

### Development of consensus-based opinion statements

Consensus-based opinion statements were developed for one of two reasons. First, if no evidence from RCTs was found. Second, if the RCT or results of two or more RCTs were inconclusive or insufficient to decide on an evidence recommendation. Consensus-based opinion statements were made based on the opinions of the Guideline panel (see Table 2). Consensus-based opinion statements required 75% agreement by the panel within three rounds of voting. Panel members needed to vote each time in one of four ways. That is, they needed to vote strongly or weakly in favour of an intervention, or strongly of weakly against an intervention. The final consensus-based opinion statements were expressed in one of these four ways. If 75% agreement was not achieved after three rounds of voting, then no consensus-based opinion statement was provided.

**Table 2:** Summary of the hierarchy of the consensus-based opinion statements.

Consensus-based opinion statements	Explanation
Strong consensus FOR	The guideline panel is confident that they can recommend the intervention based on opinion. A statement is made that the intervention should be implemented.
Weak consensus FOR	The guideline panel is confident that they can probably recommend the intervention based on opinion. A statement is made that the intervention may be implemented.
Weak consensus AGAINST	The guideline panel is confident that they probably cannot recommend the intervention based on opinion. A statement is made that the intervention should not be implemented.
Strong consensus AGAINST	The guideline panel is confident that they cannot recommend the intervention based on opinion. A statement is made that the intervention should definately not be implemented.
No consensus	The guideline panel is unable to make a statement for or against the intervention based on opinion.

Source: www.sciptguide.com.

### Development of clinical notes

Clinical notes were written to accompany evidence recommendations and consensus-based opinion statements where required. These clinical notes were written by and based on the expert opinion of the panel.

### Review of the guidelines by stakeholders

The CPG was sent out to 20 stakeholders not involved in the guideline development process. These stakeholders provided detailed feedback. Any feedback that led to revisions was approved by the Guideline Panel. Future updates or an assessment for the need for updates was recommended on review. This is planned to occur within five years of launching of the CPG.

## Results

Seventy-six RCTs met the inclusion criteria for the systematic reviews. These RCTs informed 20 meta-analyses that were used to make evidence-based recommendations and consensus-based opinion statements on 13 clinical areas within the CPG. Eleven of these clinical areas will be considered in this paper (58 RCTs and 18 meta-analyses). Two clinical areas related to respiratory management (lung volumes and respiratory muscle strength; cough and secretion clearance) will be reported in an additional paper (18 RCTs and 2 meta-analyses). The overall list of evidence-based recommendations and consensus-based opinion statements, accompanying meta-analysis and information used to make decisions can be viewed in Supplementary 1 and on www.sciptguide.com.

### Overall principles of physiotherapy management

Eighteen statements about the overall principles of physiotherapy management were voted on by the panel. There were no RCTs to inform these decisions hence these overall principles are all consensus-based statements.

### Motor skills

Twenty-four PICOs related to motor skills were voted on by the panel. Two weak evidence-based recommendations in favour of skill training were made. A weak evidence-based recommendation was made in favour of manual wheelchair skills training to improve manual wheelchair skills. The pooled results of the meta-analysis indicated that manual wheelchair skills training is better than no intervention to improve manual wheelchair skills (Standardised mean difference and 95% CI; 0.7 (0 to 1.4); see Supplementary 1) [10–13]. A weak evidence-based recommendation was also made in favour of virtual reality sitting training to improve sitting balance in people with SCI. The trial results indicate that virtual reality sitting training is better than no intervention to improve sitting balance (Mean difference and 95% CI; 63 mm (38 to 89); see Supplementary 1) [14]. These two recommendations were formed considering the results of RCTs alongside other factors and the very low certainty of evidence (as per GRADE). Thirteen strong and seven weak consensus-based opinion statements in favour of and two strong consensus-based opinion statements against different forms of skill training were also made.

### Joint mobility

Nine PICOs related to joint mobility were voted on by the panel. One weak evidence-based recommendation in favour of long duration stretch for joint mobility was made. The pooled results of the meta-analysis indicate that long duration stretch is better than no intervention to improve joint mobility (Weighted mean difference and 95% CI; 2 degrees (−1 to 5); see Supplementary 1) [15–17]. This recommendation was formed considering the results of RCTs alongside other factors and the very low certainty of evidence (as per GRADE). Eight weak consensus-based opinion statements in favour of different interventions for joint mobility were also made.

### Pain

Five PICOs related to pain were voted on by the panel. One weak evidence-based recommendation in favour of TENS to improve pain was made. The pooled results of the meta-analysis indicated that TENS is better than no intervention to improve pain (Weighted mean difference and 95% CI; −2 points (−3 to −1); see Supplementary 1) [18, 19]. This recommendation was formed considering the results of one RCT alongside other factors and the very low certainty of evidence (as per GRADE). Three strong and one weak consensus-based opinion statements in favour of different forms of pain interventions were also made.

### Strength

Five PICOs related to strength and one related to atrophy were voted on by the panel. Three weak evidence-based recommendations in favour of strength training and one against were made. First, a weak evidence-based recommendation was made in favour of strength training to improve strength of non-paralysed muscles. The trial results indicate that strength training is better than no intervention to improve strength in fully innervated muscles (Standardised mean difference not possible due to statistical heterogeneity I^2^ = 78%; see Supplementary 1) [20–22]. Second, a weak evidence recommendation was made in favour of strength training to improve strength in partially paralysed muscles. The pooled results of the meta-analysis indicate that strength training is better than no intervention to improve strength in partially paralysed muscles (Standardised mean difference and 95% CI; 0.4 (-0.1 to 0.9); see Supplementary 1) [23–25]. Third, a weak evidence recommendation was also made in favour of FES cycling to reduce atrophy in paralysed muscles. The pooled results of the meta-analysis indicated that FES cycling is better than no intervention to reduce atrophy in paralysed muscles (Standardised mean difference and 95% CI; 3 (2 to 4); See Supplementary 1) [26, 27]. A weak evidence recommendation was also made against electrical stimulation alone to improve strength in partially paralysed muscles. The trial results indicate that electrical stimulation is not better than no intervention to improve strength in partially paralysed muscles (Mean difference and 95% CI; 0 Nm (−0.5 to 0.6); see Supplementary 1) [28]. These four evidence-based recommendations were formed considering the results of RCTs alongside other factors and the very low certainty of evidence (as per GRADE). One weak for and one strong against consensus-based opinion statements were also made for the combination of strength training with electrical stimulation and vibration respectively.

### Cardiorespiratory fitness and health

Seven PICOs related to cardiorespiratory fitness and health were voted on by the panel. Three weak evidence-based recommendations in favour of fitness training were made. First, a weak evidence-based recommendation was made in favour of arm cranking to improve fitness. The pooled results of the meta-analysis indicate that arm cranking is better than no intervention to improve fitness (Weighted mean difference and 95% CI VO2 peak; 4.7 ml/kg/min (1.4 to 8.0); see Supplementary 1) [29–31]. Second, a weak evidence recommendation was made in favour of hand cycling to improve fitness. The results indicate that hand cycling is better than no intervention to improve fitness (Mean difference and 95% CI VO2 peak; 5.9 ml/kg/min (3.7 to 8.1); see Supplementary 1) [32]. Third, a weak evidence recommendation was also made in favour of circuit training for fitness. The pooled results of the meta-analysis indicate that circuit training is better than no intervention to improve fitness (Standardised mean difference and 95% CI; 0.5 (0 to 0.9); see Supplementary 1) [21, 33–35]. These three evidence recommendations were formed by considering the results of RCTs alongside other factors and the very low certainty of evidence (as per GRADE). Three strong and one weak consensus-based opinion statements for other fitness training interventions were also made.

### Swelling

Four PICOs related to interventions to prevent and treat swelling were voted on by the panel. One weak evidence-based recommendation against FES cycling for swelling was made. This recommendation was formed by considering the results of one RCT alongside other factors and the very low certainty of evidence (as per GRADE). The trial results indicate that FES cycling is not better than no intervention to improve swelling (Mean difference and 95% CI; −0.1 cm (−1.5 to 1.3); see Supplementary 1) [36]. Three weak consensus-based opinion statements in favour of different forms of interventions for swelling were also made.

### Postural hypotension

One PICO related to abdominal binders for postural hypotension was voted on by the panel. There are no RCTs on this topic. A strong consensus-based opinion statement in favour of abdominal binders to improve postural hypotension was made.

### Shoulder subluxation

Two PICOs related to interventions to prevent and treat shoulder subluxation were voted on by the panel. There are no RCTs on this topic. One strong and one weak consensus-based opinion statement in favour of equipment to support the shoulder and neuromuscular stimulation were made respectively.

### Spasticity

Four PICOs related to interventions to prevent and treat spasticity were voted on by the panel. There were three RCTs on these PICOs but evidence-based recommendations could not be made. Two weak consensus-based opinion statements for and one against interventions for spasticity were made.

### Bone mineral density

One PICO related to standing for bone mineral density was voted on by the panel. One RCT was considered, however an evidence-based recommendation or a consensus-based opinion statement could not be made.

## Discussion

This paper reports on the first comprehensive CPG for the physiotherapy management of people with SCI. Fourteen evidence-based recommendations and 85 consensus-based opinion statements across 13 clinical areas are described within the CPG. These recommendations and statements will be used by physiotherapists, consumers and their caregivers across Australia and New Zealand to guide physiotherapy management.

The first aim of this paper was to describe the methodology used to develop the CPG. A priori setting of PICO questions and using a GRADE approach for assessing the certainty of the evidence and developing recommendations are key features of our methodology [4]. Setting a priori PICO questions strengthens our CPG because the comparison of interest is defined and the outcome of interest pre-specified. The GRADE approach is broad and assesses both certainty of evidence and informs development of recommendations. Assessing certainty of evidence (as very low, low, moderate and high) is useful as it judges the quality of a body of evidence against set criteria. For example, all the evidence recommendations in our CPG rise from very low certainty evidence indicating that we have very low confidence in the effect estimate. The GRADE approach to developing recommendations is robust. It utilises a framework to develop recommendations that is both transparent and rigorous [4]. This approach has been widely used in CPGs for other health conditions but it has only been reported once for use in SCI CPGs [37].

The second aim of the paper was to provide an overview of the key recommendations and consensus-based opinion statements contained within the guideline. Due to the lack of RCT evidence our panel was only able to make 14 evidence-based recommendations. Most of our recommendations were consensus-based statements. This is due to the lack of RCTs on the PICO of interest, the RCTs being inconclusive or too low quality for the panel to decide on a definitive evidence recommendation. This highlights that our field is lacking in high quality RCTs from which to make clinical recommendations. RCT evidence for people with SCI is low across the board, however it is particularly limited in providing guidance for the management of shoulder subluxation, spasticity, decreased bone mineral density and hypotension for which we could only make consensus-based opinion statements. While the number and quality of RCTs addressing questions relevant to the physiotherapy management of people with SCI are increasing, we still have few studies to answer each PICO question. The majority of studies still have major methodological flaws, and the questions asked by researchers are not addressing treatments commonly used by physiotherapists in clinical practice suggesting inadequate stakeholder engagement in SCI research priority setting and study design.

The CPG is not without limitations. First, our list of PICOs does not cover all interventions and comparisons that physiotherapists commonly use as part of routine management. However, it does cover the top PICO questions from the perspective of physiotherapists and people with SCI. If feasible, future guidelines will update current PICOs and incorporate additional questions. Second, some may argue that evidence is broader than systematic reviews and RCTs and that lower levels of evidence such as pre-post studies should have been included in our CPG. However, including lower levels of evidence increases the chance of introducing bias. Since bias in these types of research questions tends to favour the treatment, we felt that only high levels of evidence should be used for the evidence-based recommendations. In addition, our guideline only had panel members from Australia and New Zealand. This may limit generalisability to other countries. Future guidelines with an international panel are warranted to overcome this limitation.

The CPG for the physiotherapy management of people with SCI are being widely implemented across Australia and New Zealand. The uptake of the CPG is supported by qualitative studies that identified barriers and facilitators to implementation. The CPG and accompanying website (www.sciptguide.com) are an easily accessible guide for clinical practice. It summarises, interprets and explains the evidence for physiotherapists and for people with SCI.

## Supplementary information

Glinsky - supplementary
