# Supplementary material for: An Australian and New Zealand clinical practice guideline for the physiotherapy management of people with spinal cord injuries
Source: Spinal Cord. 2025 Aug 12;63(9):470–6. doi: 10.1038/s41393-025-01088-8 (PMC12413318; doi:10.1038/s41393-025-01088-8)
Supplement: Supplementary file 1 — Glinsky - supplementary [file 41393_2025_1088_MOESM1_ESM.pdf]

2022

SCI Physiotherapy Guidelines

Guiding therapy after spinal cord injury

Australian and New Zealand Clinical Practice Guidelines for the physiotherapy management of people with Spinal Cord Injury

THE AUSTRALIAN AND NEW ZEALAND SCI PHYSIOTHERAPY CLINICAL PRACTICE GUIDELINES TEAM

Responsible organisation

The development of the Australian and New Zealand Clinical Practice Guideline for the physiotherapy management of people with spinal cord injury was coordinated by Dr Joanne Glinsky and Professor Lisa Harvey of the John Walsh Centre for Rehabilitation Research, Northern Sydney Local Health District, Sydney, Australia and The Kolling Institute, University of Sydney, Sydney Australia.

Funding sources

The Clinical Practice Guideline for the physiotherapy management of people with spinal cord injuries was funded by icare NSW, National Injury Insurance Scheme Queensland, Transport Accident Commission Victoria, and Lifetime Support Authority South Australia.

How to use this guideline

This guideline is divided into two sections. Section one provides a plain language summary and a summary of the evidence recommendations and consensus-based opinion statements. Section two provides in depth information and detailed guidance of the evidence recommendations and consensus-based opinion statements.

Physiotherapists, people with SCI, caregivers, health professionals and other stakeholders can find plain language and summary information in Section One and detailed information in Section Two of the guideline. It is assumed the recommendations provided in this guideline will be used in conjunction with relevant assessments and clinical reasoning to ensure appropriate use.

The information provided in this guideline can also be found on a website. The website is www.sciptguide.com.

Recommended citation

Glinsky JV, Harvey LA and the Australian and New Zealand Physiotherapy Clinical Practice Guidelines consortium. Australian and New Zealand Clinical Practice Guideline for the physiotherapy management of people with spinal cord injury. 2022.

Acknowledgement of country

The Australian and New Zealand Clinical Practice Guidelines team acknowledges the traditional custodians of country throughout Australia and their connections to land, sea and community. We pay our respect to their elders past and present and extend that respect to all Aboriginal and Torres Strait Islander peoples today.

Honouring Te Tiriti

Ethical approval, including Māori consultation through University of Otago, Christchurch, Te Komiti Whakarite (Canterbury District Health Board), and through Maori consultation (Counties Manukau District Health Board) was obtained for Phase 1 of the guidelines process. In developing these guidelines, we were committed to meeting our obligations under Te Tiriti and reflect the principles of tino ranagatiratanga, equity, active protection, options, and partnership.

Table of Contents

Responsible organisation ii

Funding sources ii

How to use this guideline ii

Recommended citation ii

Acknowledgement of country ii

Honouring Te Tiriti ii

Abbreviations 4

SECTION ONE 5

Plain language summary 5

About this guideline 5

Description of the guideline users 5

Summary of Methods 5

Hierarchy of the evidence recommendations and consensus-based opinion statements 6

Summary of the evidence recommendations and consensus-based opinion statements 8

1. Overall principles of physiotherapy management 8
2. Physiotherapy interventions for lung volume or respiratory muscle strength 9
3. Physiotherapy interventions for cough and secretion clearance 11
4. Physiotherapy interventions for postural hypotension 12
5. Physiotherapy interventions for motor skills 12
6. Physiotherapy interventions for pain 15
7. Physiotherapy interventions for shoulder subluxation 16
8. Physiotherapy interventions for joint mobility 17
9. Physiotherapy interventions for spasticity 18
10. Physiotherapy interventions for bone mineral density 18
11. Physiotherapy interventions for swelling 19
12. Physiotherapy interventions for strength 19
13. Physiotherapy interventions for cardiorespiratory fitness and cardiovascular health 20

SECTION TWO 22

Clinical practice guidelines for the physiotherapy management of people with SCI 22

Objective 22

Description of the guideline users 22

Description of the health condition 22

Description of the interventions 22
Description of the comparisons 23
Description of the outcomes 23
Detailed Methodology 24
Organisation of the committees 24
Composition of the committees 24
Selection of PICO questions and outcomes of interest 24
Systematic Reviews of the evidence to inform the guideline 25
Aim of the systematic reviews 25
Methods 25
Development of Recommendations 28
Assessing certainty of the evidence 29
Flow of decision making 29
Development of evidence recommendations 29
Development of Consensus-based Opinion Statements 30
Development of clinical notes 31
Evidence recommendations and consensus-based opinion statements 32
1. Overall principles of physiotherapy management 32
2. Physiotherapy interventions for lung volume or respiratory muscle strength ..39
3. Physiotherapy interventions for cough and secretion clearance 53
4. Physiotherapy interventions for postural hypotension 59
5. Physiotherapy interventions for motor skills 60
6. Physiotherapy interventions for pain 84
7. Physiotherapy interventions for shoulder subluxation 92
8. Physiotherapy interventions for joint mobility 93
9. Physiotherapy interventions for spasticity 101
10. Physiotherapy interventions for bone mineral density 106
11. Physiotherapy interventions for swelling 108
12. Physiotherapy interventions for strength 111
13. Physiotherapy interventions for fitness and cardiorespiratory health 123
Appendix 1: Additional administration details for the Guidelines 132
Guideline Management Committee 132
Guideline Management Committee members 133
Guideline Development Committee 134
Guideline Development Committee members – Rehabilitation 135

Guideline Development Committee members - Respiratory 137
Conflict of interest 139
Pre- Guideline engagement 139
Public consultation 139
Appendix 2: Additional technical details for the Guidelines 140
PICO questions 140
Search strategies 146
References 149

Abbreviations

AIS	ASIA Impairment Scale
ASIA	American Spinal Injury Association
BiPAP	Bilevel Positive Airway Pressure
CPAP	Continuous Positive Airway Pressure
ES	Electrical Stimulation
FVC	Forced Vital Capacity
FES	Functional Electrical Stimulation
GRADE	Grading of Recommendations Assessment, Development and Evaluation
IPPB	Intermittent Positive Pressure Breathing
IQR	Interquartile range
MD	Mean Difference
MIP	Mean Inspiratory Pressure
NSW	New South Wales
PICO	Participant Intervention Comparison Outcome
RCT	Randomised Controlled Trial
SCI	Spinal Cord Injury
TENS	Transcutaneous Electrical Nerve Stimulation
UL	Upper Limb
VAS	Visual Analogue Scale
VC	Vital Capacity
WUSPI	Wheelchair Users Shoulder Pain Index
95% CI	95% Confidence Interval

SECTION ONE

Plain language summary

About this guideline

The objective of this guideline is to provide clear guidance on the physiotherapy management of adults with spinal cord injury (SCI) across the continuum of care. It contains evidence recommendations and consensus-based opinion statements for over 100 questions related to the physiotherapy management of people with SCI. The questions addressed in this guideline are presented in the PICO format, namely, Participant, Intervention, Comparison and Outcome.

Description of the guideline users

Physiotherapists, people with SCI, caregivers, health professionals and other stakeholders can find summarised and detailed information within this guideline. This section (Section one) provides plain language and summarised information including a condensed version of the evidence recommendations and consensus-based opinion statements. Section two provides detailed information.

Summary of Methods

The guidelines are based on original systematic reviews of randomised controlled trials of physiotherapy interventions for adults with SCI. Questions were pre-determined and approved by a Guideline Development Committee (also known as the guideline panel). These questions are presented in the PICO format. A Grading of Recommendations Assessment, Development and Evaluation (GRADE) approach was used to develop recommendations and to assess the certainty of the evidence.[1]

A Guideline Development Committee made evidence recommendations and consensus-based opinion statements for each outcome based on a standardised process that included voting. The process for each was as follows:

1. Evidence recommendations. These were made *for* or *against* an intervention and defined as *strong* or *weak* by the guideline panel. An evidence recommendation could only be made if randomised controlled trials were identified. Each evidence recommendation was rated for certainty according to the GRADE approach where evidence was defined as very low, low, moderate, or high certainty (see table 1). No evidence recommendation was made if no randomised controlled trials were identified or if the available randomised controlled trial/s provided insufficient or inconclusive evidence.
2. Consensus-based opinion statements. These were made *for* or *against* an intervention and defined as *strong* or *weak* by the guideline panel if an evidence-base recommendation could not be made. The guideline panel voted on a statement after considering many factors including clinical experience.

These recommendations and statements required 75% agreement by the Guideline Development Committee within three rounds of voting.

Certainty	Grade definition
Very low	"The true effect is probably markedly different from the estimated effect"
Low	"The true effect might be markedly different from the estimated effect"
Moderate	"The authors believe that the true effect is probably close to the estimated effect"
High	"The authors have a lot of confidence that the true effect is similar to the estimated effect"

Table 1: GRADE certainty ratings[1]

Hierarchy of the evidence recommendations and consensus-based opinion statements

A summary of the evidence recommendations and consensus-based opinion statements are provided in the next section. These recommendations are categorised by the type of the intervention and ordered using a hierarchy. Types of intervention include but are not limited to, general management, strengthening, joint mobility, pain, fitness and motor training interventions. Evidence recommendations are ranked higher than consensus-based opinion statements. The hierarchy of evidence recommendations and consensus-based opinion statements are detailed below (see table 2 and 3).

Evidence Recommendation	Explanation
**Strong** evidence recommendation **FOR**	The guideline panel is confident that they can recommend the intervention based on the evidence.<br>A recommendation is made that the intervention should be implemented.
**Weak** evidence recommendation **FOR**	The guideline panel is confident that they can probably recommend the intervention based on the evidence.<br>A recommendation is made that the intervention may be implemented.
**Weak** evidence recommendation **AGAINST**	The guideline panel is confident that they probably cannot recommend the intervention based on the evidence.<br>A recommendation is made that the intervention should not be implemented.
**Strong** evidence recommendation **AGAINST**	The guideline panel is confident that they cannot recommend the intervention based on the evidence.<br>A recommendation is made that the intervention should definitely not be implemented.
No recommendation	The guideline panel is unable to recommend for or against the intervention based on the evidence. A consensus-based opinion statement will be made.

Table 2: Summary of the strength of the evidence recommendations. The hierarchy is based on the Grading of Recommendations Assessment, Development and Evaluation (GRADE) approach.[1]

**Consensus-based opinion statements**	**Explanation**
**Strong** consensus **FOR**	The guideline panel is confident that they can recommend the intervention based on opinion.<br>A statement is made that the intervention should be implemented.
**Weak** consensus **FOR**	The guideline panel is confident that they can probably recommend the intervention based on opinion.<br>A statement is made that the intervention may be implemented.
**Weak** consensus **AGAINST**	The guideline panel is confident that they probably cannot recommend the intervention based on opinion.<br>A statement is made that the intervention should not be implemented.
**Strong** consensus **AGAINST**	The guideline panel is confident that they cannot recommend the intervention based on opinion.<br>A statement is made that the intervention should not be implemented.
**No consensus**	The guideline panel is unable to make a statement for or against the intervention based on opinion.

Table 3: Summary of the strength of the consensus-based opinion statements.

Summary of the evidence recommendations and consensus-based opinion statements

This section provides a summary of the evidence recommendations and consensus-based opinion statements for the physiotherapy management of people with Spinal Cord Injury (SCI). These recommendations and statements were formed by the Guideline Development Committee (also known as the guideline panel). They are categorised by the type of the intervention and ordered by hierarchy of the evidence for each category. For detailed information about these recommendations and statements see Section two.

1. Overall principles of physiotherapy management

CONSENSUS-BASED OPINION STATEMENTS

**Strong** consensus-based opinion statement **FOR**	The guideline panel is confident that they can recommend the intervention based on opinion.

People with a newly acquired SCI should be managed by a multidisciplinary team including a physiotherapist within a specialised SCI unit.

People with a newly acquired SCI should receive physiotherapy assessment and treatment for the management of their impairments, activity limitations, and participation restrictions.

People with a newly acquired SCI should receive physiotherapy services throughout their acute and rehabilitation phases.

People with newly acquired SCI with respiratory muscle weakness should be assessed by a physiotherapist within 24 hours of admission to hospital.

People with existing SCI admitted for the management of a respiratory condition should be assessed by a physiotherapist within 24 hours of admission to hospital.

People with SCI should only receive physiotherapy by a registered physiotherapist or a delegate.

People with SCI should receive physiotherapy treatments that are individualised and account for any general or specific precautions and contraindications relevant to the individual.

People with SCI should be informed about all the relevant risks and benefits of different physiotherapy interventions.

People with SCI should receive person-centred care.

People with SCI should be empowered to manage their injuries including managing their physical rehabilitation and physical function.

**Strong** consensus-based opinion statement **FOR**	The guideline panel is confident that they can recommend the intervention based on opinion.

People with SCI should have the opportunity to participate in setting goals for their physiotherapy sessions that are SMART, collaborative, and regularly reviewed.

People with SCI who are prescribed exercises should be provided with a hard or electronic copy of their individualised exercise programs.

People with SCI should be assessed by a multidisciplinary team (that includes a physiotherapist) as appropriate to manage spasticity.

People with SCI should be assessed by a multidisciplinary team (that includes a physiotherapist) as appropriate to prevent and treat pressure injuries.

People with tetraplegia should be assessed by a multidisciplinary team (that includes a physiotherapist) as appropriate to determine suitability for upper limb reconstructive surgery.

People with SCI should be assessed by a physiotherapist as appropriate throughout their lives.

People with SCI should have physiotherapy treatment appropriate for the management of impairments, activity limitations or participation restrictions throughout their lives.

People with SCI and respiratory muscle weakness who are at high risk of respiratory complications should have a respiratory management plan in place when discharged into the community from hospital (including education to the care team on appropriate interventions).

People with SCI should receive appropriate equipment to maximise their independence, community participation or physical activity.

2. Physiotherapy interventions for lung volume or respiratory muscle strength

EVIDENCE RECOMMENDATIONS

**Weak** evidence recommendation **FOR**	The guideline panel is confident that they can probably recommend the intervention based on the evidence.

Respiratory muscle training may be provided to improve respiratory muscle strength in people with SCI who have respiratory muscle weakness.

Abdominal binders in sitting may be provided to improve lung volume in people with SCI who have abdominal muscle weakness or paralysis.

CONSENSUS-BASED OPINION STATEMENTS

**Strong** consensus **FOR**	The guideline panel is confident that they can recommend the intervention based on opinion.

Positioning in supine should be provided (in favour of sitting) to improve lung volumes in people with SCI who have abdominal muscle paralysis or weakness.

Intermittent application of positive pressure devices should be provided to improve lung volume in non-ventilated people with SCI who have respiratory muscle weakness. Positive pressure techniques include mechanical insufflation, Intermittent Positive Pressure Breathing (IPPB), Continuous Positive Airway Pressure (CPAP) and brief periods of Bilevel Positive Airway Pressure (BiPAP).

Intermittent application of positive pressure techniques should be provided (in consultation with medical staff) to improve lung volume in ventilated people with acute SCI that are medically stable. Intermittent application of positive pressure techniques includes ventilator hyper-inflation, manual- hyperinflation and mechanical insufflation. Ventilator hyperinflation is preferred if available.

**Weak** consensus **FOR**	The guideline panel is confident that they can probably recommend the intervention based on opinion.

Deep breathing exercises may be provided to improve lung volumes in people with SCI.

Air stacking may be taught to improve lung volume in people with SCI who have respiratory muscle weakness.

**No evidence or consensus recommendation**	The guideline panel is unable to make a statement for or against the intervention based on evidence or consensus.

No recommendation can be made on **abdominal FES** to improve lung volumes.

3. Physiotherapy interventions for cough and secretion clearance

CONSENSUS-BASED OPINION STATEMENTS

**Strong** consensus **FOR**	The guideline panel is confident that they can recommend the intervention based on opinion.

Targeted postural drainage should be provided to improve secretion clearance in people with SCI who have respiratory muscle weakness or paralysis.

Manually assisted cough should be provided to improve secretion clearance in people with SCI who have abdominal muscle weakness or paralysis and an ineffective cough.

Mechanically assisted cough (insufflation-exsufflation) should be provided to improve secretion clearance in people with SCI who have abdominal muscle weakness or paralysis and an ineffective cough.

A combination of mechanically assisted cough and manually assisted cough should be provided to improve secretion clearance in people with SCI who have abdominal muscle weakness or paralysis and an ineffective cough.

**Weak** consensus **FOR**	The guideline panel is confident that they can probably recommend the intervention based on opinion.

Percussion and vibrations may be provided to improve secretion clearance in people with SCI who have respiratory muscle weakness.

Abdominal FES may be provided to improve stimulated cough in people with SCI who have abdominal muscle weakness or paralysis.

Abdominal binders may be provided to improve cough in people with SCI who have abdominal muscle weakness or paralysis.

**Weak** consensus **AGAINST**	The guideline panel is confident that they probably cannot recommend the intervention based on opinion.

Positive expiratory pressure devices should not be provided to improve secretion clearance in people with SCI who have expiratory muscle weakness. Positive expiratory pressure techniques include use of oscillating positive pressure devices.

4. Physiotherapy interventions for postural hypotension

CONSENSUS-BASED OPINION STATEMENTS

**Strong** consensus **FOR**	The guideline panel is confident that they can recommend the intervention based on opinion.

Abdominal binders should be provided to improve postural hypotension in people with SCI.

5. Physiotherapy interventions for motor skills

EVIDENCE RECOMMENDATIONS

**Weak** evidence recommendation **FOR**	The guideline panel is confident that they can probably recommend the intervention based on the evidence.

Manual wheelchair skills training may be provided to improve manual wheelchair skills in people with SCI.

Virtual Reality sitting training may be provided in conjunction with a physiotherapist to improve the ability to sit in people with SCI.

CONSENSUS-BASED OPINION STATEMENTS

**Strong** consensus **FOR**	The guideline panel is confident that they can recommend the intervention based on opinion.

Power wheelchair skills training should be provided to improve the ability to use a power wheelchair in people with SCI

Bed mobility training should be provided to improve the ability to move in bed in people with SCI.

Sitting balance training should be provided to improve the ability to sit in people with SCI.

Sitting balance training should be provided to improve the ability to sit in people with SCI and paralysis of the lower limbs/trunk.

Transfer training should be provided to improve the ability to transfer in people with SCI.

Vertical transfer training should be provided to improve the ability to vertically transfer in people with SCI who are wheelchair dependent.

**Strong** consensus **FOR**	The guideline panel is confident that they can recommend the intervention based on opinion.

Sit to stand training should be provided to improve the ability to move from sitting to standing in people with SCI and motor function in the lower limbs.

Standing training should be provided to improve the ability to stand in people with SCI who have motor function in the lower limbs.

Stair training should be provided to improve the ability to climb stairs in people with SCI who can walk.

Upper limb and hand training (with and without FES) should be provided to improve upper limb and hand function in people with tetraplegia.

Robotic upper limb training should be provided to improve upper limb function in people with tetraplegia.

Walking training should be provided to people with SCI who have motor function in the lower limbs. Walking training could include:

- Overground gait training
- Treadmill gait training (with and without body weight support)
- Treadmill gait training with electrical stimulation (+/- body weight support)
- Overground gait training and electrical stimulation
- Robotic overground gait training
- Robotic treadmill gait training
- Conventional therapy (package of interventions including gait training)
- Gait training with orthotics

Conventional therapy (package of interventions that includes gait training) should be provided (in favour of treadmill gait training alone with or without body weight support) to improve walking in people with SCI.

**Weak** consensus **FOR**	The guideline panel is confident that they can probably recommend the intervention based on opinion.

Tenodesis splinting may be provided to improve a tenodesis grip in people with C6 and C7 tetraplegia.

Upper limb and hand function training and FES may be provided to improve hand function in people with tetraplegia.

Upper limb virtual reality training may be provided to improve upper limb function in people with tetraplegia.

Overground gait training (in favour of robotic gait training) may be provided to improve walking in people with SCI.

Overground gait training (in favour of treadmill gait training with or without body weight support) may be provided to improve walking in people with SCI.

Treadmill gait training with or without body weight support may be provided (in favour of robotic gait training) to improve walking in people with SCI.

Hydrotherapy may be provided as an adjunct to land based therapy (in favour of no intervention) to improve function in people with SCI.

**Strong** consensus **AGAINST**	The guideline panel is confident that they cannot recommend the intervention based on opinion.

Gait training (BWS or robotics) should not be provided to improve functional walking in people with SCI that have no motor function in the lower limbs.

Gait training (orthotics) should not be provided to improve functional walking in people with SCI that have no motor function in the lower limbs.

6. Physiotherapy interventions for pain

EVIDENCE RECOMMENDATIONS

**Weak** evidence recommendation **FOR**	The guideline panel is confident that they can probably recommend the intervention based on the evidence.

TENS may be provided to reduce pain in people with SCI

CONSENSUS-BASED OPINION STATEMENTS

**Strong** consensus **FOR**	The guideline panel is confident that they can recommend the intervention based on opinion.

Education to avoid shoulder overuse and trauma should be provided to prevent and treat shoulder pain in people with SCI.

Shoulder muscles vulnerable to shortening should be positioned in their lengthened position to prevent shoulder pain in people with tetraplegia.

Shoulder exercises should be provided to treat shoulder pain in people with SCI.

**Weak** consensus **FOR**	The guideline panel is confident that they can probably recommend the intervention based on opinion.

Massage therapy may be provided to treat pain in people with SCI.

**No evidence or consensus recommendation**	The guideline panel is unable to make a statement for or against the following intervention based on evidence or consensus.

No recommendation can be made on **passive movements** to prevent or treat shoulder pain in people with SCI.

7. Physiotherapy interventions for shoulder subluxation

CONSENSUS-BASED OPINION STATEMENTS

**Strong** consensus **FOR**	The guideline panel is confident that they can recommend the intervention based on opinion.

Equipment to support the shoulder such as wheelchair armrests or shoulder support devices should be provided to prevent and treat shoulder subluxation.

**Weak** consensus **FOR**	The guideline panel is confident that they can probably recommend the intervention based on opinion.

Neuromuscular electrical stimulation of the shoulder may be provided to prevent and treat shoulder subluxation in people with SCI at risk of shoulder subluxation.

8. Physiotherapy interventions for joint mobility

EVIDENCE RECOMMENDATIONS

**Weak** evidence recommendation **FOR**	The guideline panel is confident that they can probably recommend the intervention based on the evidence.

Long duration stretch may be provided to prevent and treat loss of joint mobility in people with SCI.

CONSENSUS-BASED OPINION STATEMENTS

**Weak** consensus **FOR**	The guideline panel is confident that they can probably recommend the intervention based on opinion.

Passive standing may be provided to prevent and treat loss of ROM in people with SCI and paralysed lower limbs.

Active assisted exercises may be provided to prevent loss of joint mobility in people with SCI who are at risk of contracture.

Active assisted exercises may be provided to treat loss of joint mobility in people with SCI.

Serial casting may be provided to treat contracture in people with SCI.

Hand splinting may be provided to prevent hand contracture in people with tetraplegia who are at risk of contracture.

Hand splinting may be provided to treat hand contracture in people with tetraplegia.

Upper and lower limb splinting may be provided to prevent upper and lower limb contractures in people with SCI who are at risk of contracture.

Passive range of motion exercises may be provided to prevent and treat loss of joint mobility in people with SCI.

9. Physiotherapy interventions for spasticity

CONSENSUS-BASED OPINION STATEMENTS

**Weak** consensus **FOR**	The guideline panel is confident that they can probably recommend the intervention based on opinion.

Passive standing may be provided to treat spasticity in people with SCI.

FES cycling may be provided to treat spasticity in people with SCI.

**Weak** consensus **AGAINST**	The guideline panel is confident that they probably cannot recommend the intervention based on opinion.

Passive movements should not be administered to treat spasticity in people with SCI.

**No evidence or consensus recommendation**	The guideline panel is unable to make a statement for or against the intervention based on evidence or consensus.

No evidence recommendation or consensus statement could be made about **vibration** to treat spasticity in people with SCI

10. Physiotherapy interventions for bone mineral density

CONSENSUS-BASED OPINION STATEMENTS

**No evidence or consensus recommendation**	The guideline panel is unable to make a statement for or against the intervention based on evidence or consensus

No evidence recommendation or consensus statement could be made about **passive standing** to increase bone mineral density.

11. Physiotherapy interventions for swelling

CONSENSUS-BASED OPINION STATEMENTS

**Weak** consensus **FOR**	The guideline panel is confident that they can probably recommend the intervention based on opinion.

Elevation may be provided to treat extremity swelling in people with SCI.

Neuromuscular electrical stimulation may be provided to treat extremity swelling in people with SCI.

Lymphatic massage may be provided to treat extremity swelling in people with SCI.

**Weak** evidence recommendation **AGAINST**	The guideline panel is confident that they probably cannot recommend the intervention based on the evidence.

FES cycling should not be provided to decrease swelling in people with SCI.

12. Physiotherapy interventions for strength

EVIDENCE RECOMMENDATIONS

**Weak** evidence recommendation **FOR**	The guideline panel is confident that they can probably recommend the intervention based on the evidence.

Strength training may be provided to improve voluntary strength of non-paralysed muscles in people with SCI.

Strength training may be provided to improve voluntary strength of partially paralysed muscles in people with SCI.

FES cycling may be provided to decrease atrophy in people with SCI and paralysis of the lower limbs.

**Weak** evidence recommendation **AGAINST**	The guideline panel is confident that they probably cannot recommend the intervention based on the evidence.

Electrical stimulation alone should not be provided to improve voluntary strength of partially paralysed muscles in people with SCI.

CONSENSUS-BASED OPINION STATEMENTS

**Weak** consensus **FOR**	The guideline panel is confident that they can probably recommend the intervention based on opinion.

Electrical stimulation combined with strength training may be provided to improve voluntary strength of partially paralysed muscles in people with SCI.

**Strong** consensus **AGAINST**	The guideline panel is confident that they cannot recommend the intervention based on opinion.

Whole body vibration should not be provided to improve voluntary strength in people with SCI.

13. Physiotherapy interventions for cardiorespiratory fitness and cardiovascular health

EVIDENCE RECOMMENDATIONS

**Weak** evidence recommendation **FOR**	The guideline panel is confident that they can probably recommend the intervention based on the evidence.

Arm cranking may be provided to improve cardiorespiratory fitness in people with SCI.

Hand cycling may be provided to improve cardiorespiratory fitness in people with SCI.

Circuit training may be provided to improve cardiorespiratory fitness in people with SCI.

CONSENSUS-BASED OPINION STATEMENTS

**Strong** consensus **FOR**	The guideline panel is confident that they can recommend the intervention based on opinion.

Individual or team sports should be available to improve cardiovascular health in people with SCI.

**Weak** consensus **FOR**	The guideline panel is confident that they can probably recommend the intervention based on opinion.

FES cycling may be provided to improve cardiorespiratory fitness in people with SCI.

Wheelchair pushing may be provided to improve cardiorespiratory fitness in people with SCI who are wheelchair dependent.

SECTION TWO

Clinical practice guidelines for the physiotherapy management of people with SCI

Objective

The objective of this guideline is to provide clear guidance on the physiotherapy management of adults with spinal cord injury (SCI) across the continuum of care. It contains evidence recommendations and consensus-based opinion statements for over 100 questions related to the physiotherapy management of people with SCI.

Description of the guideline users

Physiotherapists, people with SCI, caregivers, health professionals and other stakeholders can find summarised and detailed information within this guideline. This section (Section two) provides detailed information about the guideline methodology and the Evidence Recommendations and Consensus-based opinion statements. Plain language and summary information can be found in Section one.

Description of the health condition

This clinical guideline includes evidence about the physiotherapy management of adults with traumatic and non-traumatic SCI. Spinal cord injury results in damage to the spinal cord either from trauma or because of a disease process (non-traumatic). The consequence of traumatic or non-traumatic SCI are either tetraplegia or paraplegia. Tetraplegia results in loss of function in the arms and legs due to loss of motor and/or sensory function in the cervical segments of the spinal cord. Paraplegia results in loss of function in the legs and is due to loss of motor and/or sensory function in the thoracic, lumbar or sacral segments of the spinal cord. Spinal cord injury is classified according to the International Standards for Neurological Classifications of SCI. This classification system defines two motor and sensory levels (left and right) and one neurological level for each individual. It is also used to determine if an injury is complete or incomplete as per the American Spinal Injury Association (ASIA) Impairment Scale (AIS).[2]

Description of the interventions

This clinical guideline includes all physiotherapy interventions considered important by the Guideline Development Committee. These include physiotherapy interventions used in the management of people with SCI in Australia and New Zealand within the acute, rehabilitation and community settings.

Description of the comparisons

This clinical guideline includes all comparisons considered important by the Guideline Development Committee. These include comparing a physiotherapy intervention to no intervention, a sham intervention or another physiotherapy intervention.

Description of the outcomes

This clinical guideline includes all outcomes considered important by the Guideline Development Committee. These include outcomes of impairment, activity limitation or participation.

Detailed Methodology

Organisation of the committees

Two committees were responsible for the Australian and New Zealand Clinical Practice Guidelines. These were a Guideline Management Committee, and the Guideline Development Committee (also known as the guideline panel).

Composition of the committees

The broad composition of the Guideline Management and Development Committees is detailed below. Members of the Guideline Management Committee were approached directly by the Chairperson. Members of the Guideline Development Committee were either approached directly by the chairperson or appointed by their organisation. All members were involved in developing terms of reference, scope and processes for each committee. Only members of the Guideline Development Committee were involved in the development of the evidence recommendations and consensus-based statements within the guideline. Members and composition of the two committees can be viewed in Appendix One.

Selection of PICO questions and outcomes of interest

The questions addressed in this guideline are presented in the PICO format, namely, Participant, Intervention, Comparison and Outcome. The PICO questions were determined prior to commencing the guideline reviews. Decisions about interventions that are routine clinical practice within the Australian and New Zealand context were made by an expert committee of physiotherapists within the Guideline Development Committee. This Committee met to discuss and formulate the PICO questions. During this process the drafted PICO questions were adopted, rejected, or changed. A list of PICO questions was approved by the Guideline Development Committee and completed prior to commencement of the guideline reviews. Additional PICO questions were added at the request of the committee during the development and guideline review process. The PICO questions can be found in Appendix Two.

PICO questions focused on impairment and activity-based physical outcomes rather than global health concerns with reference to culture or race, cultural practice or world views. As such, other outcomes that contribute or reflect participation and well-being (part of the Maori models of health) have not been included in these guidelines.

Systematic Reviews of the evidence to inform the guideline

Aim of the systematic reviews

A systematic review was conducted on each PICO. The aim of each systematic review was to determine the effectiveness of each physiotherapy intervention compared with no intervention, a sham intervention or another physiotherapy intervention on outcomes of impairment, activity limitation or participation.

Methods

Types of studies

Published randomised controlled trials (RCTs) and randomised controlled cross over trials were included. Trials with more than two parallel comparisons were included if two of the comparisons met the inclusion criteria. If trials were reported in more than one publication or interim analyses were published prior to the completion of the trial, then the most recent publication was used. Trials published only in English were included.

Types of participants

Adults (> 16 years) with a traumatic or non-traumatic SCI were included. Trials with a mixture of participants with different neurological conditions were only included if 80% or greater of participants within the trial had an SCI. Congenital condition involving the spinal cord such as spina bifida were excluded.

Types of interventions

All physiotherapy interventions identified in the list of PICOs were included. These were all interventions considered routine clinical practice in Australia and New Zealand.

Types of comparisons

Trials were included if they compared the interventions of interest with no intervention or a sham intervention. Trials that compared interventions with an alternate intervention were also included if they were a PICO of interest. Trials that included a co-intervention or usual care were included if the co-interventions or usual care were administered to both groups (making it possible to determine the added benefit of the intervention of interest).

Types of outcome measures

Trials were included that contained an outcome relevant to each PICO. These typically included measures of impairment, activity limitation and participation restriction. In situations where there was more than one measure of an outcome, we chose the outcome without looking at the results of the trial. A decision rule was used that prioritised measures considered important to clinicians and people with SCI.

Search methods for identification of studies

The following electronic databases were searched to identify reports of relevant studies: Ovid MEDLINE (1946 to August 13th 2020); Ovid EMBASE (1974 to August 13th 2020); EBSCO CINAHL Plus (1937 to August 13th 2020); Physiotherapy Evidence Database (PEDro) (Searched August 13th 2020) and CENTRAL on August 13th 2020.

To search Medline and Embase we used the OVID search strategy for RCTs combined with search terms for SCI. To search CINAHL we used the Cochrane search strategy for RCTs combined with search terms for SCI. To search PEDro we used category Neurotrauma combined with category RCTs. To search Central we used terms for SCI. Full search strategies can be found in the technical section of the guideline (Appendix One).

Searching other resources

In addition, we searched the reference lists of all identified RCTs and systematic reviews.

Selection of studies

Two authors independently screened the identified titles and abstracts using the pre-defined inclusion criteria detailed above. When required, the full text was then assessed to determine whether the trial met the inclusion criteria. If the trial met the inclusion criteria it was included. One author then selected studies from the identified list and matched them to each PICO question. If the trial did not meet the inclusion criteria it was excluded. Disagreements were resolved by discussion.

Data extraction and management

The data was extracted from the studies and recorded on an excel spreadsheet. One author independently extracted descriptive data.

The data extracted included:

- trial methodology, type of trial and design of trial
- trial participants including age, gender, neurological level of SCI, AIS classification of SCI, type of SCI (traumatic or non-traumatic) and time since injury
- the experimental intervention including type, frequency, dosage of exercise or any details of the intervention provided
- the comparison intervention
- the co-interventions in the experimental and the comparison group
- the outcome measure
- the trial including authors, year of publication, setting and country

Details of data extraction for synthesis

Two authors independently extracted data for each study to determine mean between-group differences and 95% confidence intervals (95% CI). This included outcome

scores and number of participants overall and in each group. Data were estimated from graphs if necessary. The following rules were used (from first to last) when deciding upon which data to extract:

- mean between-group difference in post-intervention scores, adjusted for baseline scores.
- mean and standard deviation (SD) of change scores provided in the studies (post-intervention scores and change scores were not pooled in meta-analyses in which results were expressed as standardised mean differences (SMD)).
- mean (SD) post-intervention scores.

If only medians and inter-quartile ranges (IQR) were provided, medians were extracted and used as means, and SDs were estimated by dividing the interquartile range by 1.35. Cross-over studies were analysed using first period data or combined data if first period were not available. RevMan 5.4.1 software was be used to convert 95% CIs, standard errors, p values and any other appropriate combination of data or statistical results into SDs when necessary. The direction of effect of each outcome was standardised.

Meta-analyses were conducted across studies that made similar comparisons if there were at least two studies without excessive clinical or statistical heterogeneity. Clinical heterogeneity was assessed by examining the type of participants, type and intensity of the intervention, and other issues related to the design and conduct of the studies. Statistical heterogeneity was quantified using the I^2 statistic where an $I^2 > 75\%$ was considered to indicate excessive heterogeneity and results were not pooled. A fixed-effects model was used to pool data if the I^2 was less than 50%, and a random-effects model was used if the I^2 was between 50 and 75%. If studies in a meta-analysis used the same measure and same units, effects were expressed as mean differences (MD) and 95% CI. If different measures or different units were used within a meta-analysis, effects were expressed as SMD and 95% CI. In calculating SMD post-intervention scores were not pooled with change scores. Data were analysed using RevMan v5.4.1. No sub-group or sensitivity analysis were performed.

Assessment of risk of bias in included studies

The risk of bias in each trial was assessed by one reviewer and checked by one reviewer using the five domains of Version 2 of the Cochrane risk-of bias tool.[3] The domains assessed were potential bias arising from: the randomisation process; deviations from intended interventions; missing outcome data; measurement of the outcome; selection of the reported result.

The level of potential bias was judged as low, high or unclear (due to a lack of information or uncertainty) for each domain. Disagreements were resolved by discussion.

The PEDro score for each study was also extracted from the PEDro database.[4] If scores were not available on the database one author assessed the score for the study.

Measures of treatment effect

Continuous data that used the same units were expressed as mean differences with 95% confidence intervals (CI). Continuous outcomes that use different units were expressed using SMD with 95% CI. Dichotomous outcomes were expressed as risk

ratios (RR) with 95% CI. Time to event data were expressed as hazard ratios (HR) with 95% CI. Data were pooled in meta-analyses where appropriate and reasonable.

Unit of analysis issues

Unit of analysis issues were considered in the following three cases:

1. Cross-over trials

   In cross-over trials data were analysed from the first period if available. Data for different periods within the trial were only used if first period data were not provided within the study.

2. Trials used in meta-analysis in which more than one type of intervention was compared

   In trials that compared two or more types of interventions with no training or a sham group, data were analysed for all intervention groups. Double-counting of the control or sham group participants was avoided by using all data from the groups but dividing data from the control or sham groups by the number of groups.

3. Trials where multiple measures were taken on the same participant

   In trials where multiple measures were taken on the same participant data at the end of the intervention period were used.

Dealing with missing data

All feasible available results were included. Authors were only contacted for missing data where clarifications were required. However, no data obtained from authors was used in the guideline. Only published data was extracted to use in analysis. All available data were converted where possible (for example, when data were reported as standard errors) using the calculator incorporated into Review Manager. If results were only presented graphically, we estimated the mean scores and SDs from graphs if it was reasonable to do so.

Assessment of heterogeneity

Data were pooled in a meta-analysis if there were two or more studies, there was clinical homogeneity (studies with similar interventions, participants and outcomes) and not excessive statistical heterogeneity (see details of data extraction for synthesis).

Development of Recommendations

The GRADE approach was used for the development of recommendations. This approach is based on the GRADE handbook.[1]

The Guideline Development Committee made recommendations for each outcome based on a standardised process that included voting. Evidence recommendations for or against an intervention were defined as strong or weak by the guideline panel. No (neutral) recommendation was made when the panel was unable to recommend for or against the intervention based on the evidence. Where no recommendation could be

made or no evidence existed on which to base a recommendation, the Guideline Development Committee voted on a consensus-based opinion statement.

Assessing certainty of the evidence

The evidence from each systematic review for each PICO was independently graded for certainty by two reviewers. The GRADE approach was used where the certainty of the evidence is defined as very low, low, moderate or high certainty.

Certainty	Grade definition
Very low	"The true effect is probably markedly different from the estimated effect"
Low	"The true effect might be markedly different from the estimated effect"
Moderate	"The authors believe that the true effect is probably close to the estimated effect"
High	"The authors have a lot of confidence that the true effect is similar to the estimated effect"

Table 4 The GRADE ratings used to rate the certainty of evidence.[1]

Flow of decision making

A detailed flow chart of the decision-making process can be found in Appendix two of the guideline.

Development of evidence recommendations

All evidence recommendations were made by initially considering the size and precision of treatment effects along with the quality of the evidence. We then took into account the balance between benefits and harms, values and preferences, resource use and other relevant considerations including equity, accessibility and feasibility. These considerations were documented by two authors on an evidence to decision table. [1] The direction of the recommendation was expressed using the language described by GRADE as a recommendation for an intervention, against an intervention or no recommendation. The strength of a recommendation for or against an intervention was expressed as Strong or Weak. This recommendation required 75% agreement by the Guideline Development Committee within three rounds of voting. Definitions from the GRADE Handbook were used throughout the guideline development process.[1]

GRADE defines a **STRONG** recommendation as:

"*A strong recommendation is one for which guideline panel is confident that the desirable effects of an intervention outweigh its undesirable effects (for an intervention) or that the undesirable effects of an intervention outweigh its desirable effects (against an intervention).*"

GRADE defines a **WEAK** recommendation as:

"A weak recommendation is one for which the desirable effects probably outweigh the undesirable effects (for an intervention) or undesirable effects probably outweigh the desirable effects (against an intervention) but appreciable uncertainty exists."

GRADE defines **NO** recommendation as justified when:

"The panel feels a recommendation is too speculative or the panel has difficulty deciding on the direction of the recommendation."[1]

Evidence Recommendation	Explanation
**Strong** evidence recommendation **FOR**	The guideline panel is confident that they can recommend the intervention based on the evidence.<br>A recommendation is made that the intervention should be implemented.
**Weak** evidence recommendation **FOR**	The guideline panel is confident that they can probably recommend the intervention based on the evidence.<br>A recommendation is made that the intervention may be implemented.
**Weak** evidence recommendation **AGAINST**	The guideline panel is confident that they probably cannot recommend the intervention based on the evidence.<br>A recommendation is made that the intervention should not be implemented.
**Strong** evidence recommendation **AGAINST**	The guideline panel is confident that they cannot recommend the intervention based on the evidence.<br>A recommendation is made that the intervention should definitely not be implemented.
No recommendation	The guideline panel is unable to recommend for or against the intervention based on the evidence. A consensus-based opinion statement will be made.

Table 5: Summary of the strength of the evidence recommendations. The hierarchy is based on the A Grading of Recommendations Assessment, Development and Evaluation (GRADE) approach.[1]

Development of Consensus-based Opinion Statements

Consensus-based opinion statements are defined by the National Health and Medical Research Council in their guidelines procedures and requirements as:

"*Recommendations formulated in the absence of quality evidence (where a systematic review of the evidence was conducted as part of the search strategy)"*[5]

In this guideline consensus-based opinion statements were developed for one of two reasons.

1. Evidence was found from the systematic review, but the panel decided that no evidence recommendation could be made. This decision was made if the randomised controlled trials contained inconclusive or insufficient evidence.

2. Evidence was not found from the systematic review. This decision was made if no randomised controlled trials were found.

Consensus-based opinion statement were made based on the expert opinions of the Guidelines Development Committee. This opinion was developed by considering the evidence or lack thereof, balance between benefits and harms, values and preferences, resource use, personal experience and other relevant considerations. Consensus-based opinion statement required 75% agreement by the committee within three rounds of voting. If 75% agreement was not achieved after three rounds of voting, then no consensus was reached.

The direction of the consensus-based opinion statements were expressed as *for* an intervention, *against* an intervention or *no statement*. No statement was given when a consensus could not be reached, or the committee did not feel it was important to make a statement. The strength of the statement in each direction was expressed as Strong or Weak.

Consensus-based opinion statements	Explanation
**Strong** consensus **FOR**	The guideline panel is confident that they can recommend the intervention based on opinion.<br>A statement is made that the intervention should be implemented.
**Weak** consensus **FOR**	The guideline panel is confident that they can probably recommend the intervention based on opinion.<br>A statement is made that the intervention may be implemented.
**Weak** consensus **AGAINST**	The guideline panel is confident that they probably cannot recommend the intervention based on opinion.<br>A statement is made that the intervention should not be implemented.
**Strong** consensus **AGAINST**	The guideline panel is confident that they cannot recommend the intervention based on opinion.<br>A statement is made that the intervention should not be implemented.
**No consensus**	The guideline panel is unable to make a statement for or against the intervention based on opinion.

Table 6: Summary of the strength of the consensus-based opinion statements

Development of clinical notes

Clinical notes were written to accompany evidence recommendations and consensus-based opinion statements where required. These clinical notes were based on the expert opinion of the committee.

Evidence recommendations and consensus-based opinion statements

1. Overall principles of physiotherapy management

CONSENSUS-BASED OPINION STATEMENTS

	Physiotherapy assessment and treatment		
**P**	People with a newly acquired SCI	**Evidence recommendation**<br>◯ No evidence recommendation<br>Reason: No RCTs	**Strong opinion statement FOR**<br>People with a newly acquired SCI should receive physiotherapy assessment and treatment for the management of their impairments, activity limitations, and participation restrictions.
**I**	Physiotherapy assessment and treatment		
**C**	No intervention	**Consensus-based opinion statement**<br>● Strong for (100%)	
**O**	Optimal outcome		

	Physiotherapy Services		
**P**	People with a newly acquired SCI	**Evidence recommendation**<br>◯ No evidence recommendation<br>Reason: No RCTs	**Strong opinion statement FOR**<br>People with a newly acquired SCI should receive physiotherapy services throughout their acute and rehabilitation phases.
**I**	Physiotherapy Services		
**C**	Optimal outcome	**Consensus-based opinion statement**<br>● Strong for (100%)	
**O**	Not stated		

<table>
<tr><th></th><th colspan="3">Respiratory assessment by a physiotherapist within 24 hours of admission to hospital (newly acquired)</th></tr>
<tr><td>P</td><td>People with a newly acquired SCI with respiratory muscle weakness</td><td rowspan="2">Evidence recommendation
◯ No evidence recommendation
Reason: No RCTs</td><td rowspan="4">Strong opinion statement <u>FOR</u>
People with newly acquired SCI with respiratory muscle weakness should be assessed by a physiotherapist within 24 hours of admission to hospital.</td></tr>
<tr><td>I</td><td>Assessment by a physiotherapist within 24 hours of admission to hospital</td></tr>
<tr><td>C</td><td>No intervention</td><td rowspan="2">Consensus-based opinion statement
● Strong for (100%)</td></tr>
<tr><td>O</td><td>Optimal outcome</td></tr>
</table>

<table>
<tr><th></th><th colspan="3">Respiratory assessment by a physiotherapist within 24 hours of admission to hospital (existing SCI and management of respiratory condition)</th></tr>
<tr><td>P</td><td>People with existing SCI admitted for the management of a respiratory condition</td><td rowspan="2">Evidence recommendation
◯ No evidence recommendation
Reason: No RCTs</td><td rowspan="4">Strong opinion statement <u>FOR</u>
People with existing SCI admitted for the management of a respiratory condition should be assessed by a physiotherapist within 24 hours of admission to hospital.</td></tr>
<tr><td>I</td><td>Respiratory Assessment by a physiotherapist within 24 hours of admission to hospital</td></tr>
<tr><td>C</td><td>No intervention</td><td rowspan="2">Consensus-based opinion statement
● Strong for (100%)</td></tr>
<tr><td>O</td><td>Optimal outcome</td></tr>
</table>

	Physiotherapy treatment by a registered physiotherapist or a delegate		
**P**	People with SCI	**Evidence recommendation**<br>○ No evidence recommendation<br>Reason: No RCTs	**Strong opinion statement FOR**<br>Physiotherapy treatments for people with SCI should be provided by a registered physiotherapist or a delegate.
**I**	Physiotherapy treatment by a registered physiotherapist or a delegate		
**C**	No intervention	**Consensus-based opinion statement**<br>● Strong for (92%)	
**O**	Optimal outcome		

	Physiotherapy treatments that are individualised		
**P**	People with SCI	**Evidence recommendation**<br>○ No evidence recommendation<br>Reason: No RCTs	**Strong opinion statement FOR**<br>People with SCI should receive physiotherapy treatments that are individualised and account for any general or specific precautions and contraindications relevant to the individual.<br><br>Clinical note: Some interventions have the potential to increase damage to the spine or spinal cord in people with recently acquired/acute SCI. Therefore, they should be administered according to informed local policies and procedures and/or after medical clearance.
**I**	Physiotherapy treatments that are individualised		
**C**	No intervention	**Consensus-based opinion statement**<br>● Strong for (100%)	
**O**	Not stated		

	Informed about all the relevant risks and benefits of different physiotherapy interventions		
**P**	People with SCI	**Evidence recommendation**<br>○ No evidence recommendation<br>Reason: No RCTs	**Strong opinion statement FOR**<br>People with SCI should be informed about all the relevant risks and benefits of different physiotherapy interventions.
**I**	Informed about all the relevant risks and benefits of different physiotherapy interventions		
**C**	No intervention	**Consensus-based opinion statement**<br>● Strong for (96%)	
**O**	Optimal outcome		

	Person centred care		
**P**	People with SCI	**Evidence recommendation**<br>○ No evidence recommendation<br>Reason: No RCTs	**Strong opinion statement FOR**<br>People with SCI should receive person-centred care.
**I**	Person centred care		
**C**	No intervention	**Consensus-based opinion statement**<br>● Strong for (100%)	
**O**	Optimal outcome		

	Empowered to manage their injuries		
**P**	People with SCI	**Evidence recommendation**<br>○ No evidence recommendation<br>Reason: No RCTs	**Strong opinion statement FOR**<br>People with SCI should be empowered to manage their injuries including managing their physical rehabilitation and physical function.
**I**	Empowered to manage their injuries		
**C**	No intervention	**Consensus-based opinion statement**<br>● Strong for (100%)	
**O**	Optimal outcome		

	SMART Goals		
**P**	People with SCI	**Evidence recommendation**<br>○ No evidence recommendation<br>Reason: No RCTs	**Strong opinion statement FOR**<br>People with SCI should have the opportunity to participate in setting goals for their physiotherapy sessions that are SMART, collaborative, and regularly reviewed.
**I**	SMART goals		
**C**	No intervention	**Consensus-based opinion statement**<br>● Strong for (96%)	
**O**	Optimal outcome		

	Provision of hard or electronic copy of individualised exercise programs		
**P**	People with SCI	**Evidence recommendation**<br>○ No evidence recommendation<br>Reason: No RCTs	**Strong opinion statement <u>FOR</u>**<br>People with SCI who are prescribed exercises should be provided with a hard or electronic copy of their individualised exercise programs.
**I**	Provision of hard or electronic copy of individualised exercise programs		
**C**	No intervention	**Consensus-based opinion statement**<br>● Strong for (86%)	
**O**	Optimal outcome		

	Assessment by a multidisciplinary team for UL reconstructive surgery		
**P**	People with tetraplegia	**Evidence recommendation**<br>○ No evidence recommendation<br>Reason: No RCTs	**Strong opinion statement <u>FOR</u>**<br>People with tetraplegia should be assessed by a multidisciplinary team (that includes a physiotherapist) as appropriate to determine suitability for upper limb reconstructive surgery.
**I**	Assessment by a multidisciplinary team for Upper Limb reconstructive surgery		
**C**	No intervention	**Consensus-based opinion statement**<br>● Strong for (100%)	
**O**	Optimal outcome		

	Assessment by a multidisciplinary team for spasticity management		
**P**	People with SCI	**Evidence recommendation**<br>○ No evidence recommendation<br>Reason: No RCTs	**Strong opinion statement <u>FOR</u>**<br>People with SCI should be assessed by a multidisciplinary team (that includes a physiotherapist) as appropriate to manage spasticity.
**I**	Assessment by a multidisciplinary team for spasticity management		
**C**	No intervention	**Consensus-based opinion statement**<br>● Strong for (100%)	
**O**	Optimal outcome		

	Assessment by a multidisciplinary team for prevention and treatment of pressure injuries		
**P**	People with SCI	**Evidence recommendation**<br>○ No evidence recommendation<br>Reason: No RCTs	**Strong opinion statement FOR**<br>People with SCI should be assessed by a multidisciplinary team (that includes a physiotherapist) as appropriate to prevent and treat pressure injuries.
**I**	Assessment by a multidisciplinary team for prevention and treatment of pressure injuries		
**C**	No intervention	**Consensus-based opinion statement**<br>● Strong for (100%)	
**O**	Optimal outcome		

	Assessment by a physiotherapist throughout the lifetime		
**P**	People with SCI	**Evidence recommendation**<br>○ No evidence recommendation<br>Reason: No RCTs	**Strong opinion statement FOR**<br>People with SCI should be assessed by a physiotherapist as appropriate throughout their lives.
**I**	Assessment by a physiotherapist as appropriate throughout the lifetime		
**C**	No intervention	**Consensus-based opinion statement**<br>● Strong for (83%)	
**O**	Optimal outcome		

	Physiotherapy as appropriate throughout the lifetime		
**P**	People with SCI	**Evidence recommendation**<br>○ No evidence recommendation<br>Reason: No RCTs	**Strong opinion statement FOR**<br>People with SCI should have physiotherapy treatment as appropriate for the management of impairments, activity limitations or participation opportunities throughout their lives.
**I**	Physiotherapy as appropriate throughout the lifetime		
**C**	No intervention	**Consensus-based opinion statement**<br>● Strong for (96%)	
**O**	Optimal outcome		

	Discharged into the community with a respiratory management plan		
**P**	People with SCI and respiratory muscle weakness who are at high risk of respiratory complications	**Evidence recommendation**<br>○ No evidence recommendation<br>Reason: No RCTs	**Strong opinion statement <u>FOR</u>**<br>People with SCI and respiratory muscle weakness who are at high risk of respiratory complications should be discharged into the community from hospital with a respiratory management plan in place (including education to the care team on appropriate interventions).
**I**	Respiratory management plan		
**C**	No intervention	**Consensus-based opinion statement**<br>● Strong for (100%)	
**O**	Optimal outcome		

	Appropriate equipment to maximise independence		
**P**	People with SCI	**Evidence recommendation**<br>○ No evidence recommendation<br>Reason: No RCTs	**Strong opinion statement <u>FOR</u>**<br>People with SCI should receive appropriate equipment to maximise their independence, community participation or physical activity.
**I**	Appropriate equipment to maximise independence		
**C**	No intervention	**Consensus-based opinion statement**<br>● Strong for (96%)	
**O**	Optimal outcome		

2. Physiotherapy interventions for lung volume or respiratory muscle strength

EVIDENCE RECOMMENDATIONS

	**Inspiratory muscle training (v no intervention) on inspiratory respiratory muscle strength in people with SCI who have respiratory muscle weakness**					
**P**	People with SCI who have respiratory muscle weakness	**Evidence recommendation**<br>◯ Weak for (100%)		**Weak evidence recommendation FOR**<br>Respiratory muscle training may be used to improve respiratory muscle strength in people with SCI who have respiratory muscle weakness.<br><br>Clinical note: Inspiratory muscle training is most commonly used in clinical practice but training can also include expiratory muscle training. Inspiratory muscle training is usually done with a training device.		
**I**	Respiratory muscle training					
**C**	No intervention	**Opinion statement**<br>◯ No opinion statements				
**O**	Muscle strength (mean inspiratory pressure)					
	**SUMMARY**	10 RCTs[6--15]		Mean difference (95% CI): Muscle strength in Mean Inspiratory Pressure<br>-13 (-17 to -9)<br>Favours respiratory muscle training		
	**GRADE**<br>Very low certainty<br>⊕◯◯◯	**Risk of bias**<br>Serious	**Inconsistency**<br>Serious	**Imprecision**<br>No serious	**Indirectness**<br>No serious	**Publication bias**<br>Serious

	Experimental			Control				Mean Difference
**Study or Subgroup**	**Mean**	**SD**	**Total**	**Mean**	**SD**	**Total**	**Weight**	**IV, Fixed, 95% CI**
Boswell-Ruys 2020	-66.4	11.6522	29	-54.9	11.6522	31	47.9%	-11.50 [-17.40, -5.60]
Liaw 2000	-58.6	16.7	10	-63.1	17.9	10	7.2%	4.50 [-10.67, 19.67]
Litchke 2008	-107.5	21.2	4	-102.4	18.5	5	2.4%	-5.10 [-31.45, 21.25]
Litchke 2011	-106.4	16.09	5	-88.29	23.96	7	3.2%	-18.11 [-40.78, 4.56]
Loveridge 1989	-100.7	19.3	6	-105.3	16.3	6	4.1%	4.60 [-15.61, 24.81]
Mueller 2013	-35.38	29.43	8	-8.88	15.21	8	3.2%	-26.50 [-49.46, -3.54]
Postma 2014	-82.7	29.7	19	-70.7	28.1	21	5.2%	-12.00 [-29.97, 5.97]
Roth 2010	-71	30.6657	16	-56	30.6657	13	3.3%	-15.00 [-37.44, 7.44]
Soumyashree 2018	-99.9	11.3293	15	-78.3	11.3293	12	22.5%	-21.60 [-30.20, -13.00]
West 2014	-135	33.541	5	-116	33.541	5	1.0%	-19.00 [-60.58, 22.58]
**Total (95% CI)**			**117**			**118**	**100.0%**	**-12.71 [-16.79, -8.63]**

Heterogeneity: $Chi^2 = 14.08$, df = 9 (P = 0.12); $I^2 = 36\%$
Test for overall effect: Z = 6.10 (P < 0.00001)

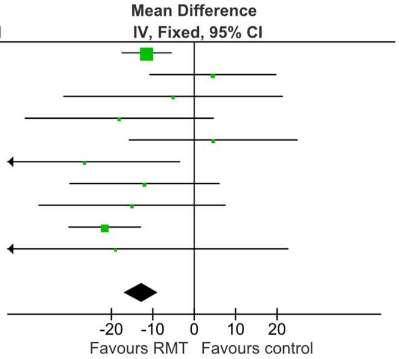

**INSPIRATORY MUSCLE TRAINING FOR RESPIRATORY MUSCLE STRENGTH: GRADE Evidence to Decision**						
**Problem**	No	Probably no	Probably yes	**Yes**		Don't know
**Desirable Effects**	Trivial	Small	**Moderate**	Large		Don't know
**Undesirable Effects**	Large	Moderate	Small	Trivial		**Don't know**

INSPIRATORY MUSCLE TRAINING FOR RESPIRATORY MUSCLE STRENGTH: GRADE Evidence to Decision						
**Certainty of evidence**	**Very low**	Low	Moderate	High		No included studies
**How Much PEOPLE Value THE MAIN OUTCOME**	Important uncertainty or variability	Possibly important uncertainty or variability	**Probably no important uncertainty or variability**	No important uncertainty or variability		
**Balance of effects**	Favours the Control	Probably favours the Control	Does not favour either the intervention (I) or the comparison (C)	Probably favours the I	**Favours the I**	Don't know
**Resources required**	Large costs	Moderate costs	**Negligible costs and savings**	Moderate savings	Large savings	Don't know
**Certainty of evidence of required resources**	Very low	Low	Moderate	High		**No included studies**
**Cost effectiveness**	Favours the comparison	Probably favours the comparison	Does not favour either the intervention or the comparison	Probably favours the intervention	Favours the intervention	**No included studies**
**Equity**	Reduced	Probably reduced	**Probably no impact**	Probably increased	Increased	Don't know
**Acceptability**	No	Probably no	**Probably yes**	Yes		Don't know
**Feasibility**	No	Probably no	**Probably yes**	Yes		Don't know

INSPIRATORY MUSCLE TRAINING FOR RESPIRATORY MUSCLE STRENGTH: Randomised Controlled Trial Details

STUDY	COMPARISON	DOSAGE/DETAILS	PARTICIPANTS	N (RX/C)	OUTCOME	ROB 2 PEDRO
**BOSWELL-RUYS 2020**	Resistive Inspiratory muscle training (RMT) V Sham RMT	3-5 sets 12 breaths 2 x day 5 days per week for 6 weeks @ > 30% MIP	C4-C8 SCI AIS A,B,C > 4 weeks post injury	29/31	Maximal Inspiratory pressure (MIP)	Very low Risk of Bias PEDro = 10/10
**LIAW 2000**	Inspiratory muscle training (& usual care) V Usual care	15-20 minutes 2 x day; 7 days per week for 6/52	C4-C7 complete SCI <6months post injury	10/10	MIP	High Risk of Bias PEDro = 4/10
**LITCHKE 2008**	Respiratory resistance training V No intervention	1 set of exercises 2-3 x per day daily for 10 weeks	>80% participants with SCI C5-T12 SCI >6months post injury	4/5	MIP	Some Concerns about Risk of Bias PEDro = 5/10
**LITCHKE 2011**	Concurrent flow resistance V No intervention	10 breaths 3 different x per day daily for 9 weeks	>80% participants with SCI C5-C7 SCI	5/7	MIP	High Risk of Bias PEDro = 3/10
**LOVERIDGE 1989**	Inspiratory muscle training V	85% of sustained inspiratory pressure 2 x day for 15 minutes	C6-C7 complete SCI >1 year post injury	6/6	MIP	Some Concerns about Risk of Bias PEDro = 4/10

INSPIRATORY MUSCLE TRAINING FOR RESPIRATORY MUSCLE STRENGTH: Randomised Controlled Trial Details

	No intervention	5 days per week for 8 weeks				
**MUELLER 2013**	Inspiratory resistance training<br>V<br>placebo	90 breaths @ > 80% max inspiratory power<br>4 x per week for 8 weeks	C5-C8 complete SCI<br>6-8 months post injury	8/8	MIP	High Risk of Bias<br>PEDro = 5/10
**POSTMA 2014**	Resistive Inspiratory muscle training (& usual care)<br>V<br>Usual care	7 sets of 2 minutes @ 60% MIP<br>5 x week for 8 weeks	T12 and above SCI AIS A-D initial rehab FEV_1 <80% predicted	19/21	MIP	High Risk of Bias<br>PEDro = 7/10
**ROTH 2010**	Expiratory muscle training<br>V<br>Sham	Exp muscle resistive training<br>10 reps, twice a day, 5 x per week for 6 weeks	T1 and above motor complete SCI	16/13	MIP	High Risk of Bias<br>PEDro = 4/10
**SOUMYASHREE 2018**	Inspiratory muscle training<br>V<br>Breathing exercises	15 minutes @ 40 MIP<br>5 x per week for 4 weeks	T1-12 SCI<br>AIS A-D	15/12	MIP	Some Concerns of Risk of bias<br>PEDro = 7/10
**WEST 2014**	Inspiratory muscle training<br>V<br>Sham	30 breaths at 50-60% Pimax<br>2 x day<br>5 days per week for 6 weeks	C5-C7 SCI<br>AIS A or B<br>≥3 years post injury	5/5	MIP	High Risk of Bias<br>PEDro = 4/10

Abdominal binders in sitting (v no intervention) on lung volumes in people with SCI who have respiratory muscle weakness

**P**	People with SCI who have respiratory muscle weakness	**Evidence recommendation**<br>◯ Weak for (100%)	**Weak evidence** recommendation **FOR**<br>Abdominal binders in sitting may be used to improve lung volume in people with SCI.<br>Clinical note: Abdominal binders (to improve lung volumes) are provided in people with respiratory compromise and abdominal muscle paralysis (full or partial). Abdominal binders may not be suitable for people with significant abdominal distension, central adiposity, or large abdomens. Abdominal binders may also be provided for purposes other than improving lung volume.
**I**	Abdominal binders		
**C**	No abdominal binder	**Consensus-based opinion statement**<br>◯ No opinion statements	
**O**	Lung volume		
	**SUMMARY**	5 RCTs[16-20]	Mean difference (95% CI): Lung volume in litres<br>0.3 (0.1 to 0.5)<br>Favours abdominal binders

**GRADE**	**Risk of bias**	**Inconsistency**	**Imprecision**	**Indirectness**	**Publication bias**
Very Low certainty<br>⊕◯◯◯	Very serious	No serious	No serious	Serious	Serious

Study or Subgroup	Experimental_AB_sitting Mean	SD	Total	Control no_AB_sitting Mean	SD	Total	Weight	Mean Difference IV, Fixed, 95% CI
Boaventura 2003	2.07	2.0555	10	2.39	2.0239	10	1.3%	-0.32 [-2.11, 1.47]
Bodin 2005	3.3	1	20	3	1	20	11.1%	0.30 [-0.32, 0.92]
Goldman 1986	2.53	0.85	7	2.27	0.84	7	5.5%	0.26 [-0.63, 1.15]
Hart 2005	2.41	0.89	10	2.04	0.81	10	7.7%	0.37 [-0.38, 1.12]
Wadsworth 2012	3.06	0.324	14	2.72	0.324	14	74.3%	0.34 [0.10, 0.58]
**Total (95% CI)**			**61**			**61**	**100.0%**	**0.32 [0.12, 0.53]**

Heterogeneity: Chi² = 0.56, df = 4 (P = 0.97); I² = 0%
Test for overall effect: Z = 3.07 (P = 0.002)

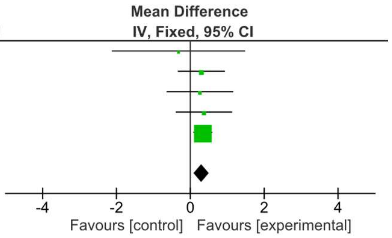

ABDOMINAL BINDERS FOR LUNG VOLUME: GRADE Evidence to Decision						
PROBLEM	No	Probably no	Probably yes	**Yes**		Don't know
DESIRABLE EFFECTS	Trivial	**Small**	Moderate	Large		Don't know
UNDESIRABLE EFFECTS	Large	Moderate	Small	**Trivial**		Don't know
CERTAINTY OF EVIDENCE	**Very low**	Low	Moderate	High		No included studies
HOW MUCH PEOPLE VALUE THE MAIN OUTCOME	Important uncertainty or variability	Possibly important uncertainty or variability	**Probably no important uncertainty or variability**	No important uncertainty or variability		
BALANCE OF EFFECTS	Favours the Control	Probably favours the Control	Does not favour either the intervention (I) or the comparison (C)	Probably favours the I	**Favours the I**	Don't know
RESOURCES REQUIRED	Large costs	Moderate costs	**Negligible costs and savings**	Moderate savings	Large savings	Don't know

ABDOMINAL BINDERS FOR LUNG VOLUME: GRADE Evidence to Decision						
CERTAINTY OF EVIDENCE OF REQUIRED RESOURCES	Very low	Low	Moderate	High		**No included studies**
COST EFFECTIVENESS	Favours the comparison	Probably favours the comparison	Does not favour either the intervention or the comparison	Probably favours the intervention	Favours the intervention	**No included studies**
EQUITY	Reduced	Probably reduced	**Probably no impact**	Probably increased	Increased	Don't know
ACCEPTABILITY	No	Probably no	Probably yes	**Yes**		Don't know
FEASIBILITY	No	Probably no	Probably yes	**Yes**		Don't know

ABDOMINAL BINDERS FOR LUNG VOLUME: Randomised Controlled Trial Details

STUDY	COMPARISON	DOSAGE/DETAILS	PARTICIPANTS	N (RX/C)	OUTCOME	ROB 2 PEDRO
**GOLDMAN 1996**	Sitting with abdominal binder V Sitting without abdominal binder	Elastic binder	C5-C7 Complete SCI >3 months post injury	7/7	Lung volume Vital Capacity (VC)	High Risk of Bias PEDro = 5/10
**BOAVENTURA 2003**	Sitting with abdominal binder V Sitting without abdominal binder	Elastic binder	C4-C7 Complete SCI 1 year post injury	10/10	Lung volume Forced Vital Capacity (FVC)	Some Concerns of Risk of bias PEDro = 6/10
**BODIN 2005**	Sitting with abdominal binder V Sitting without abdominal binder	Elastic binder	C5-C8 SCI At least 1 year post injury	20/20	Lung volume (VC)	High Risk of Bias PEDro = 4/10
**HART 2005**	Sitting with abdominal binder V Sitting without abdominal binder	Combination elastic and non-elastic binder	C5-T6 AIS A SCI	10/10	Lung volume (FVC)	High Risk of Bias PEDro = 4/10
**WADSWORTH 2012**	Sitting with abdominal binder V Sitting without abdominal binder	Elastic binder	C3-T5 AIS A or AIS B SCI Acute	14/14	Lung volume (FVC)	High Risk of Bias PEDro = 4/10

CONSENSUS-BASED OPINION STATEMENTS

	Supine (v sitting) on lung volumes in people with SCI who have abdominal muscle paralysis (full or partial)		
**P**	People with SCI who have abdominal muscle paralysis (full or partial)	**Evidence recommendation**<br>◯ No evidence recommendation<br>Reason: No recommendation due to insufficient or inconclusive evidence.	**Strong opinion statement <u>FOR</u>**<br>Positioning in supine should be provided (in favour of sitting) to improve lung volumes in people with SCI who have abdominal muscle paralysis or weakness.<br>Clinical note: Supine may not be suitable for people with significant abdominal distension, central adiposity or those with large abdomens and long-term SCI.
**I**	Supine		
**C**	Sitting	**Consensus-based opinion statement**<br>● Strong for (85%)	
**O**	Lung volume		
	**SUMMARY**	1 RCT[16]	Mean difference (95% CI): Lung volume in litres<br>0.4 (-1.3 to 2.1)<br>Favours supine

GRADE	Risk of bias	Inconsistency	Imprecision	Indirectness	Publication bias
**GRADE**<br>Very low certainty<br>⊕◯◯◯	Very serious	Serious	Serious	Serious	Serious

Study or Subgroup	supine Mean	supine SD	supine Total	sitting Mean	sitting SD	sitting Total	Mean Difference IV, Fixed, 95% CI
Boaventura 2003	2.77	1.8657	10	2.39	2.0239	10	0.38 [-1.33, 2.09]

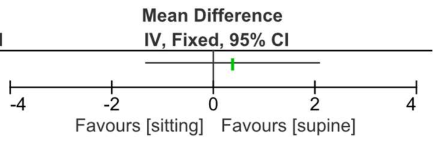

SUPINE (V SITTING) FOR LUNG VOLUMES: GRADE Evidence to Decision						
**PROBLEM**	No	Probably no	Probably yes	**Yes**		Don't know
**DESIRABLE EFFECTS**	Trivial	Small	Moderate	Large		**Don't know**
**UNDESIRABLE EFFECTS**	Large	Moderate	Small	Trivial		**Don't know**
**CERTAINTY OF EVIDENCE**	**Very low**	Low	Moderate	High		No included studies
**HOW MUCH PEOPLE VALUE THE MAIN OUTCOME**	Important uncertainty or variability	Possibly important uncertainty or variability	**Probably no important uncertainty or variability**	No important uncertainty or variability		
**BALANCE OF EFFECTS**	Favours the Control	Probably favours the Control	**Does not favour either the intervention (I) or the comparison (C)**	Probably favours the I	Favours the I	Don't know
**RESOURCES REQUIRED**	Large costs	Moderate costs	**Negligible costs and savings**	Moderate savings	Large savings	Don't know

SUPINE (V SITTING) FOR LUNG VOLUMES: GRADE Evidence to Decision						
**CERTAINTY OF EVIDENCE OF REQUIRED RESOURCES**	Very low	Low	Moderate	High		**No included studies**
**COST EFFECTIVENESS**	Favours the comparison	Probably favours the comparison	Does not favour either the intervention or the comparison	Probably favours the intervention	Favours the intervention	**No included studies**
**EQUITY**	Reduced	Probably reduced	**Probably no impact**	Probably increased	Increased	Don't know
**ACCEPTABILITY**	No	Probably no	Probably yes	**Yes**		Don't know
**FEASIBILITY**	No	Probably no	Probably yes	**Yes**		Don't know

SUPINE FOR LUNG VOLUME: Randomised Controlled Trial Details

STUDY	COMPARISON	DOSAGE/DETAILS	PARTICIPANTS	N (RX/C)	OUTCOME	ROB 2 PEDRO
**BOAVENTURA 2003**	Supine V Sitting	Elastic binder in sitting and supine	C4-C7 Complete SCI 1 year post injury	10/10	Lung volume (FVC)	High Risk of Bias PEDro = 6/10

Intermittent application of positive pressure devices (v no intervention) on lung volume in non-ventilated people with SCI who have respiratory muscle weakness

**P**	People with SCI who are not ventilated and have respiratory muscle weakness	**Evidence recommendation**<br>○ No evidence recommendation<br>Reason: No recommendation due to insufficient or inconclusive evidence.	**Strong opinion statement FOR**<br>Intermittent application of positive pressure devices should be provided to improve lung volume in non-ventilated people with SCI who have respiratory muscle weakness.<br><br>Clinical note: Contraindications and precautions for the use of positive pressure devices must be considered before prescribing these treatments. For example, positive pressure devices are contraindicated in conditions that include but are not limited to untreated pneumothorax, tracheoesophageal fistula and acute traumatic brain injury with increased or poorly controlled intracranial pressure. Positive pressure devices include mechanical insufflation, Intermittent Positive Pressure Breathing (IPPB), Continuous Positive Airway pressure (CPAP) and brief periods of Bilevel Positive Airway Pressure (BiPAP).
**I**	Intermittent application of positive pressure devices		
**C**	No intervention	**Consensus-based opinion statement**<br>● Strong for (93%)	
**O**	Lung volume (Litres)		
	**SUMMARY**	1 RCT[21]	Mean difference (95% CI): Lung volume in litres<br>0.1 (-0.5 to 0.7)<br>Favours intermittent positive pressure breathing

GRADE	Risk of bias	Inconsistency	Imprecision	Indirectness	Publication bias
Very low certainty ⊕○○○	Very serious	Serious	Serious	Serious	Serious

Study or Subgroup	Experimental Mean	Experimental SD	Experimental Total	Control Mean	Control SD	Control Total	Weight	Mean Difference IV, Fixed, 95% CI
Laffont et al 2008	2.84	0.82	14	2.71	0.78	14		0.13 [-0.46, 0.72]

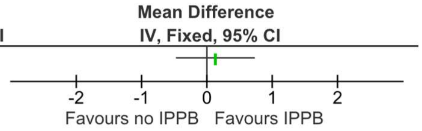

INTERMITTENT APPLICATION OF POSITIVE PRESSURE DEVICES ON LUNG VOLUME: GRADE Evidence to Decision						
**PROBLEM**	No	Probably no	Probably yes	**Yes**		**Don't know**
**DESIRABLE EFFECTS**	Trivial	Small	Moderate	Large		**Don't know**
**UNDESIRABLE EFFECTS**	Large	Moderate	Small	Trivial		**Don't know**
**CERTAINTY OF EVIDENCE**	**Very low**	Low	Moderate	High		No included studies
**HOW MUCH PEOPLE VALUE THE MAIN OUTCOME**	Important uncertainty or variability	Possibly important uncertainty or variability	**Probably no important uncertainty or variability**	No important uncertainty or variability		
**BALANCE OF EFFECTS**	Favours the Control	Probably favours the Control	**Does not favour either the intervention (I) or the comparison (C)**	Probably favours the I	Favours the I	Don't know

**INTERMITTENT APPLICATION OF POSITIVE PRESSURE DEVICES ON LUNG VOLUME: GRADE Evidence to Decision**						
**RESOURCES REQUIRED**	Large costs	Moderate costs	**Negligible costs and savings**	Moderate savings	Large savings	Don't know
**CERTAINTY OF EVIDENCE OF REQUIRED RESOURCES**	Very low	Low	Moderate	High		**No included studies**
**COST EFFECTIVENESS**	Favours the comparison	Probably favours the comparison	Does not favour either the intervention or the comparison	Probably favours the intervention	Favours the intervention	**No included studies**
**EQUITY**	Reduced	Probably reduced	**Probably no impact**	Probably increased	Increased	Don't know
**ACCEPTABILITY**	No	Probably no	Probably yes	**Yes**		Don't know
**FEASIBILITY**	No	Probably no	Probably yes	**Yes**		Don't know

INTERMITTENT POSITIVE PRESSURE FOR LUNG VOLUME: Randomised Controlled Trial Details

STUDY	COMPARISON	DOSAGE/DETAILS	PARTICIPANTS	N (RX/C)	OUTCOME	ROB 2 PEDRO
**LAFFONT 2008**	Intermittent positive pressure breathing (IPPB) V No intervention	IPPB up to 40cmH20 20mins 2 x per day 5 days per week or 2 months	C5-T6 Complete SCI <6months post injury	14/14	Lung volume (VC)	High Risk of Bias PEDro = 5/10

	Intermittent application of positive pressure (v no intervention) on lung volume in ventilated people with SCI who have respiratory muscle weakness		
**P**	People with SCI who are ventilated and have respiratory muscle weakness	**Evidence recommendation**<br>○ No evidence recommendation<br>Reason: No RCTs	**Strong opinion statement FOR**<br>Intermittent application of positive pressure therapy techniques should be used (in consultation with medical staff) for improving lung volume in ventilated people with acute SCI that are medically stable.<br><br>Clinical note: Positive pressure therapy techniques include ventilator hyper-inflation, mechanical insufflation and manual-hyperinflation. Ventilator hyperinflation is preferred if available. Positive pressure techniques are contraindicated in conditions that include but are not limited to untreated pneumothorax, tracheoesophageal fistula, increased intracranial pressure and facial trauma.
**I**	Intermittent application of positive pressure therapy techniques		
**C**	No intervention	**Consensus-based opinion statement**<br>● Strong for (100%)	
**O**	Lung volume (Litres)		

	Deep breathing exercises (v no intervention) on lung volumes in people with SCI who have respiratory muscle weakness		
**P**	People with SCI who have respiratory muscle weakness	**Evidence recommendation**<br>○ No evidence recommendation<br>Reason: No RCTs	**Weak opinion statement FOR**<br>Deep breathing exercises may be provided to improve lung volumes in people with SCI.<br><br>Clinical note: People with SCI and respiratory muscle weakness should focus on respiratory strength training exercises, rather than deep breathing exercises.<br><br>Deep breathing exercises (including with the use of Incentive Spirometers) should not be provided as the only treatment to improve lung volumes in people with SCI who have respiratory muscle paralysis.
**I**	Deep breathing exercises		
**C**	No intervention	**Consensus-based opinion statement**<br>● Weak for (100%)	
**O**	Lung volume		

Air stacking (v no intervention) on lung volumes in people with SCI who have respiratory muscle weakness

**P**	People with SCI who have respiratory muscle weakness	**Evidence recommendation**<br>◯ No evidence recommendation<br>Reason: No recommendation due to insufficient or inconclusive evidence.		**Weak opinion statement FOR**<br>Air stacking may be taught to improve lung volume in people with SCI who have respiratory muscle weakness.<br><br>Clinical note: Air stacking involves the use of any positive pressure inspiratory device. These should be provided by a mouthpiece and nose peg rather than a face mask because of the risk of pneumothorax with facemasks.		
**I**	Air stacking					
**C**	No intervention	**Consensus-based opinion statement**<br>● Weak for (100%)				
**O**	Lung volume (L)					
	**SUMMARY**	1 RCT[22]		Mean difference (95% CI): Lung volume in litres<br>0 (-0.6 to 0.6)<br>Favours air stacking		
	**GRADE**<br>Very low certainty<br>⊕◯◯◯	**Risk of bias**<br>Very serious	**Inconsistency**<br>Serious	**Imprecision**<br>Serious	**Indirectness**<br>Serious	**Publication bias**<br>Serious

Study or Subgroup	Experimental Mean	Experimental SD	Experimental Total	Control Mean	Control SD	Control Total	Mean Difference IV, Fixed, 95% CI
Jeong 2015	1.85	0.91	14	1.83	0.76	12	0.02 [-0.62, 0.66]

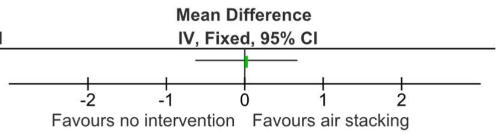

AIR STACKING ON LUNG VOLUME: GRADE Evidence to Decision						
**PROBLEM**	No	Probably no	Probably yes	**Yes**		Don't know
**DESIRABLE EFFECTS**	Trivial	Small	Moderate	Large		**Don't know**
**UNDESIRABLE EFFECTS**	Large	Moderate	Small	Trivial		**Don't know**
**CERTAINTY OF EVIDENCE**	**Very low**	Low	Moderate	High		No included studies
**HOW MUCH PEOPLE VALUE THE MAIN OUTCOME**	Important uncertainty or variability	Possibly important uncertainty or variability	**Probably no important uncertainty or variability**	No important uncertainty or variability		
**BALANCE OF EFFECTS**	Favours the Control	Probably favours the Control	**Does not favour either the intervention (I) or the comparison (C)**	Probably favours the I	Favours the I	Don't know
**RESOURCES REQUIRED**	Large costs	Moderate costs	**Negligible costs and savings**	Moderate savings	Large savings	Don't know
**CERTAINTY OF EVIDENCE OF REQUIRED RESOURCES**	Very low	Low	Moderate	High		**No included studies**

AIR STACKING ON LUNG VOLUME: GRADE Evidence to Decision						
**COST EFFECTIVENESS**	Favours the comparison	Probably favours the comparison	Does not favour either the intervention or the comparison	Probably favours the intervention	Favours the intervention	**No included studies**
**EQUITY**	Reduced	Probably reduced	**Probably no impact**	Probably increased	Increased	Don't know
**ACCEPTABILITY**	No	Probably no	Probably yes	**Yes**		Don't know
**FEASIBILITY**	No	Probably no	Probably yes	**Yes**		Don't know

AIR STACKING FOR LUNG VOLUME: Randomised Controlled Trial Details

STUDY	COMPARISON	DOSAGE/DETAILS	PARTICIPANTS	N (RX/C)	OUTCOME	ROB 2 PEDRO
**JEONG 2015**	Air stacking V Incentive spirometry	20 reps air stacking 2 x per day 5 days per week for 6 weeks	tetaplegia	14/12	Lung volume (FVC)	High Risk of Bias PEDro = 6/10

	**Abdominal FES (v no intervention) on lung volumes in people with SCI who have respiratory muscle weakness**					
**P**	People with SCI who have respiratory muscle weakness	**Evidence recommendation**<br>◯ No evidence recommendation<br>Reason: No recommendation due to insufficient or inconclusive evidence.		**No evidence recommendation or consensus-based opinion statement**		
**I**	Abdominal FES					
**C**	No intervention	**Consensus-based opinion statement**<br>◯ No consensus statements<br>Reason: No consensus could be reached				
**O**	Lung volume (Litres)					
	**SUMMARY**	1 RCT[23]		Mean difference (95% CI): Lung volume in litres<br>0.4 (-0.1 to 0.9)<br>Favours abdominal FES		
	**GRADE**<br>Very low certainty<br>⨁◯◯◯	**Risk of bias**<br>Very serious	**Inconsistency**<br>No serious	**Imprecision**<br>Serious	**Indirectness**<br>No serious	**Publication bias**<br>Serious

	Experimental			Control			Mean Difference
Study or Subgroup	Mean	SD	Total	Mean	SD	Total	IV, Fixed, 95% CI
Cheng 2006	2.26	0.7	13	1.82	0.6	13	0.44 [-0.06, 0.94]

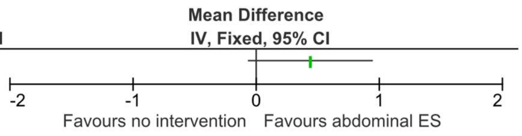

ABDOMINAL FES ON LUNG VOLUME: GRADE Evidence to Decision						
PROBLEM	No	Probably no	Probably yes	**Yes**		Don't know
DESIRABLE EFFECTS	Trivial	**Small**	Moderate	Large		Don't know
UNDESIRABLE EFFECTS	Large	Moderate	Small	Trivial		**Don't know**
CERTAINTY OF EVIDENCE	**Very low**	Low	Moderate	High		No included studies
HOW MUCH PEOPLE VALUE THE MAIN OUTCOME	Important uncertainty or variability	Possibly important uncertainty or variability	**Probably no important uncertainty or variability**	No important uncertainty or variability		
BALANCE OF EFFECTS	Favours the Control	Probably favours the Control	Does not favour either the intervention (I) or the comparison (C)	**Probably favours the I**	Favours the I	Don't know
RESOURCES REQUIRED	Large costs	Moderate costs	**Negligible costs and savings**	Moderate savings	Large savings	Don't know
CERTAINTY OF EVIDENCE OF REQUIRED RESOURCES	Very low	Low	Moderate	High		**No included studies**

**ABDOMINAL FES ON LUNG VOLUME: GRADE Evidence to Decision**						
**COST EFFECTIVENESS**	Favours the comparison	Probably favours the comparison	Does not favour either the intervention or the comparison	Probably favours the intervention	Favours the intervention	**No included studies**
**EQUITY**	Reduced	Probably reduced	**Probably no impact**	Probably increased	Increased	Don't know
**ACCEPTABILITY**	No	Probably no	Probably yes	**Yes**		Don't know
**FEASIBILITY**	No	Probably no	**Probably yes**	Yes		Don't know

ABDOMINAL FES FOR LUNG VOLUME: Randomised Controlled Trial Details

STUDY	COMPARISON	DOSAGE/DETAILS	PARTICIPANTS	N (RX/C)	OUTCOME	ROB 2 PEDRO
**CHENG 2006**	NMES plus usual care V Usual Care	NMES 30Hz; pulse width 300µs; on/off 4/4s; Intensity 0 to 100mA.	C4-C7 SCI AIS A, B <3 months post injury	13/13	Lung volume (FVC)	Some concerns about Risk of Bias PEDro = 5/10

3. Physiotherapy interventions for cough and secretion clearance

CONSENSUS-BASED OPINION STATEMENTS

	Targeted postural drainage (v no intervention) on secretion clearance in people with SCI who have respiratory muscle weakness		
**P**	People with SCI who have respiratory muscle weakness	**Evidence recommendation**<br>○ No evidence recommendation<br>Reason: No RCTs	**Strong opinion statement FOR**<br><br>Targeted postural drainage should be provided to improve secretion clearance in people with SCI who have respiratory muscle weakness or paralysis.<br><br>Clinical note: Postural drainage (including head down tilt) is usually provided as an adjunct to other respiratory therapies. Head down tilt is contraindicated in conditions that include but are not limited to heart failure, reflux and acute Traumatic Brain Injury with increased/poorly controlled intracranial pressure.
**I**	Postural drainage		
**C**	No intervention	**Consensus-based opinion statement**<br>● Strong for (85%)	
**O**	Secretion clearance		

	Manually assisted cough (v no intervention) on secretion clearance in people with SCI who have abdominal muscle weakness or paralysis and an ineffective cough		
**P**	People with SCI who have abdominal muscle paralysis (full or partial)	**Evidence recommendation**<br>○ No evidence recommendation<br>Reason: No RCTs	**Strong opinion statement FOR**<br><br>Consensus Statement: Manually assisted cough should be provided to improve secretion clearance in people with SCI who have abdominal muscle weakness or paralysis and an ineffective cough.<br><br>Clinical note: Manually assisted cough is contraindicated in conditions such as recent abdominal trauma. Manually assisted cough should be considered with caution in people with paralytic ileus or rib fractures.
**I**	Manually assisted cough		
**C**	No intervention	**Consensus-based opinion statement**<br>● Strong for (100%)	
**O**	Secretion clearance		

<table>
<tr><th></th><th colspan="3">Mechanically assisted cough (Insufflation/exsufflation) (v no intervention) on secretion clearance in people with SCI who have abdominal muscle weakness or paralysis and an ineffective cough</th></tr>
<tr><td>P</td><td>People with SCI who have respiratory muscle weakness.</td><td rowspan="2">Evidence recommendation
No evidence recommendation
Reason: No RCTs</td><td rowspan="4">Strong opinion statement <u>FOR</u>

Mechanically assisted cough (Insufflation-exsufflation) should be provided to improve secretion clearance in people with SCI who have abdominal muscle weakness or paralysis and an ineffective cough.

Clinical Note: This treatment is particularly important for those at high risk of secretion retention.

Positive pressure devices are contraindicated in conditions that include but are not limited to untreated pneumothorax, tracheoesophageal fistula and acute traumatic brain injury with increased/poorly controlled intracranial pressure.</td></tr>
<tr><td>I</td><td>Mechanically assisted cough (Insufflation-exsufflation)</td></tr>
<tr><td>C</td><td>No intervention</td><td rowspan="2">Consensus-based opinion statement
Strong for (93%)</td></tr>
<tr><td>O</td><td>Secretion clearance</td></tr>
</table>

<table>
<tr><th></th><th colspan="3">Mechanically assisted cough (Insufflation/exsufflation) plus manually assisted cough (v no intervention) on secretion clearance in people with SCI who have abdominal muscle weakness or paralysis and an ineffective cough</th></tr>
<tr><td>P</td><td>People with SCI who have abdominal muscle paralysis (full or partial).</td><td rowspan="2">Evidence recommendation
No evidence recommendation
Reason: No RCTs</td><td rowspan="4">Strong opinion statement <u>FOR</u>
A combination of mechanically assisted cough (insufflation-exsufflation) and manually assisted cough should be provided to improve secretion clearance in people with SCI who have abdominal muscle weakness or paralysis and an ineffective cough.

Clinical note: Insufflation-exsufflation and manually assisted cough can be provided independently or in combination for increasing secretion clearance in people with SCI.</td></tr>
<tr><td>I</td><td>A combination of mechanically assisted cough (Insufflation-exsufflation) and manually assisted cough</td></tr>
<tr><td>C</td><td>No intervention</td><td rowspan="2">Consensus-based opinion statement
Strong for (100%)</td></tr>
<tr><td>O</td><td>Secretion clearance</td></tr>
</table>

	**Percussion and vibration (v no intervention) on secretion clearance in people with SCI who have respiratory muscle weakness**		
**P**	People with SCI who have respiratory muscle weakness.	**Evidence recommendation**<br>○ No evidence recommendation<br>Reason: No RCTs	**Weak opinion statement FOR**<br>Consensus Statement: Percussion and vibrations may be provided to improve secretion clearance in people with SCI who have respiratory muscle weakness.<br><br>Clinical note: Percussion and vibrations are usually provided as an adjunct to other respiratory therapies.
**I**	Percussion and vibration		
**C**	No intervention	**Consensus-based opinion statement**<br>● Weak for (85%)	
**O**	Secretion clearance		

	**Abdominal FES (v no intervention) on stimulated cough in people with SCI who have abdominal muscle paralysis or weakness**					
**P**	People with SCI who have abdominal muscle weakness.	**Evidence recommendation**<br>○ No evidence recommendation<br>Reason: No recommendation due to insufficient or inconclusive evidence.		**Weak opinion statement FOR**<br>FES to the abdominal muscles may be provided to improve stimulated cough in people with SCI who have abdominal muscle paralysis or weakness.		
**I**	Abdominal FES					
**C**	No intervention	**Consensus-based opinion statement**<br>● Weak for (92%)				
**O**	Secretion clearance					
	**SUMMARY**	1 RCT[23]		Mean difference (95% CI): Peak Expiratory Flow in Litres<br>1 (0.4 to 1.7)<br>Favours abdominal FES		
	**GRADE**<br>Very low certainty<br>⊕○○○	**Risk of bias**<br>Serious	**Inconsistency**<br>Serious	**Imprecision**<br>No serious	**Indirectness**<br>No serious	**Publication bias**<br>Serious

Study or Subgroup	experimental Mean	SD	Total	control Mean	SD	Total	Mean Difference IV, Fixed, 95% CI
Cheng 2006	3.93	0.9	13	2.9	0.7	13	1.03 [0.41, 1.65]

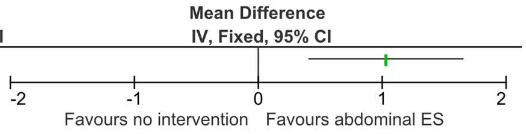

ABDOMINAL FES ON PEAK EXPIRATORY FLOW: GRADE Evidence to Decision						
PROBLEM	No	Probably no	Probably yes	**Yes**		Don't know
DESIRABLE EFFECTS	Trivial	**Small**	Moderate	Large		Don't know
UNDESIRABLE EFFECTS	Large	Moderate	Small	Trivial		**Don't know**
CERTAINTY OF EVIDENCE	**Very low**	Low	Moderate	High		No included studies
HOW MUCH PEOPLE VALUE THE MAIN OUTCOME	Important uncertainty or variability	Possibly important uncertainty or variability	**Probably no important uncertainty or variability**	No important uncertainty or variability		
BALANCE OF EFFECTS	Favours the Control	Probably favours the Control	Does not favour either the intervention (I) or the comparison (C)	Probably favours the I	**Favours the I**	Don't know
RESOURCES REQUIRED	Large costs	Moderate costs	**Negligible costs and savings**	Moderate savings	Large savings	Don't know
CERTAINTY OF EVIDENCE OF REQUIRED RESOURCES	Very low	Low	Moderate	High		**No included studies**
COST EFFECTIVENESS	Favours the comparison	Probably favours the comparison	Does not favour either the intervention or the comparison	Probably favours the intervention	Favours the intervention	**No included studies**
EQUITY	Reduced	Probably reduced	**Probably no impact**	Probably increased	Increased	Don't know
ACCEPTABILITY	No	Probably no	**Probably yes**	Yes		Don't know
FEASIBILITY	No	Probably no	**Probably yes**	Yes		Don't know

FOR PEAK EXPIRATORY FLOW: Randomised Controlled Trial Details

STUDY	COMPARISON	DOSAGE/DETAILS	PARTICIPANTS	N (RX/C)	OUTCOME	ROB 2 PEDRO
**CHENG 2006**	NMES plus usual care V Usual Care	NMES 30Hz; pulse width 300µs; on/off 4/4s; Intensity 0 to 100mA.	C4-C7 SCI AIS A or B <3 months post injury	13/13	Peak Expiratory Flow (PEF)	Some concerns about Risk of Bias PEDro = 5/10

Abdominal binders (v no intervention) to improve cough in people with SCI who have abdominal muscle paralysis (full or partial)

**P**	People with SCI who have abdominal muscle weakness.	**Evidence recommendation**<br>○ No evidence recommendation<br>Reason: No recommendation due to insufficient or inconclusive evidence.	**Weak opinion statement FOR**<br>Abdominal binder may be provided to improve cough in people with SCI who have abdominal muscle weakness.<br><br>Clinical note: Abdominal binders (to improve cough) are provided in people with abdominal paralysis (partial or full) and may not be suitable for people significant abdominal distension, central adiposity or large abdomens. Abdominal binders may also be provided for purposes other than improving cough.
**I**	Abdominal binder		
**C**	No intervention	**Consensus-based opinion statement**<br>● Weak for (100%)	
**O**	Secretion clearance		
	**SUMMARY**	1 RCT[17]	Mean difference (95% CI): Peak expiratory flow in Litres<br>0.8 (0.1 to 1.5)<br>Favours abdominal binder

GRADE	Risk of bias	Inconsistency	Imprecision	Indirectness	Publication bias
Very low certainty ⊕○○○	Serious	Serious	No serious	No serious	Serious

Study or Subgroup	Experimental Mean	Experimental SD	Experimental Total	Control Mean	Control SD	Control Total	Weight	Mean Difference IV, Fixed, 95% CI
Wadsworth 2012	4.67	0.735	14	3.86	1.07	14		0.81 [0.13, 1.49]

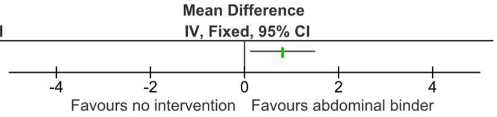

ABDOMINAL BINDERS ON COUGH: GRADE Evidence to Decision						
**PROBLEM**	No	Probably no	Probably yes	**Yes**		Don't know
**DESIRABLE EFFECTS**	Trivial	**Small**	Moderate	Large		Don't know
**UNDESIRABLE EFFECTS**	Large	Moderate	Small	Trivial		**Don't know**
**CERTAINTY OF EVIDENCE**	**Very low**	Low	Moderate	High		No included studies
**HOW MUCH PEOPLE VALUE THE MAIN OUTCOME**	Important uncertainty or variability	Possibly important uncertainty or variability	**Probably no important uncertainty or variability**	No important uncertainty or variability		
**BALANCE OF EFFECTS**	Favours the Control	Probably favours the Control	Does not favour either the intervention (I) or the comparison (C)	Probably favours the I	**Favours the I**	Don't know
**RESOURCES REQUIRED**	Large costs	Moderate costs	**Negligible costs and savings**	Moderate savings	Large savings	Don't know
**CERTAINTY OF EVIDENCE OF REQUIRED RESOURCES**	Very low	Low	Moderate	High		**No included studies**

ABDOMINAL BINDERS ON COUGH: GRADE Evidence to Decision						
**COST EFFECTIVENESS**	Favours the comparison	Probably favours the comparison	Does not favour either the intervention or the comparison	Probably favours the intervention	Favours the intervention	**No included studies**
**EQUITY**	Reduced	Probably reduced	**Probably no impact**	Probably increased	Increased	Don't know
**ACCEPTABILITY**	No	Probably no	**Probably yes**	Yes		Don't know
**FEASIBILITY**	No	Probably no	**Probably yes**	Yes		Don't know

ABDOMINAL BINDERS FOR PEAK EXPIRATORY FLOW: Randomised Controlled Trial Details

STUDY	COMPARISON	DOSAGE/DETAILS	PARTICIPANTS	N (RX/C)	OUTCOME	ROB 2 PEDRO
**WADSWORTH 2012**	Sitting with abdominal binder V Sitting without abdominal binder	Elastic binder	C3-T5 SCI AIS A or BI Acute	14/14	Peak Expiratory Flow	High Risk of Bias PEDro = 4/10

	**Positive expiratory pressure devices (v no intervention) on secretion clearance in people with SCI who have expiratory muscle weakness**		
**P**	People with SCI who have respiratory muscle weakness.	**Evidence recommendation**<br>○ No evidence recommendation<br>Reason: No RCTs	**Weak opinion statement <u>AGAINST</u>**<br>Positive expiratory pressure devices should not be provided to improve secretion clearance in people with SCI who have expiratory muscle weakness.<br><br>Clinical note: Positive expiratory pressure techniques include oscillating positive pressure devices.
**I**	Positive expiratory pressure devices		
**C**	No intervention	**Consensus-based opinion statement**<br>● Weak against (75%)	
**O**	Secretion clearance		

4. Physiotherapy interventions for postural hypotension

CONSENSUS-BASED OPINION STATEMENTS

<table>
<tr><th colspan="4">Abdominal binders v no intervention on postural hypotension in people with SCI</th></tr>
<tr><td>P</td><td>People with SCI who have abdominal muscle paralysis (full or partial).</td><td rowspan="2">Evidence recommendation
◯ No evidence recommendation
Reason: No RCTs</td><td rowspan="4">Strong opinion statement <u>FOR</u>

Abdominal binders should be provided to improve postural hypotension in people with SCI.

Clinical note: Abdominal binders are only provided in people with abdominal paralysis (partial or full) and may not be suitable for people significant abdominal distension, central adiposity or large abdomens. Abdominal binders may also be provided for purposes other than postural hypotension.</td></tr>
<tr><td>I</td><td>Abdominal binders</td></tr>
<tr><td>C</td><td>No intervention</td><td rowspan="2">Consensus-based opinion statement
● Strong for (83%)</td></tr>
<tr><td>O</td><td>Postural hypotension</td></tr>
</table>

5. Physiotherapy interventions for motor skills

EVIDENCE RECOMMENDATIONS

	Manual wheelchair skills training (v no intervention) on wheelchair skills in people with SCI					
**P**	People with SCI	**Evidence recommendation**<br>◯ Weak for (95%)		**Weak evidence** recommendation **FOR**<br>Evidence recommendation: manual wheelchair skills training may be provided to improve manual wheelchair skills in people with SCI.		
**I**	Manual wheelchair skills training					
**C**	No intervention	**Consensus-based opinion statement**<br>◯ No opinion statements				
**O**	Wheelchair skills					
	**SUMMARY**	5 RCTs[24-27]		Standardised Mean difference (95% CI):<br>0.7 (0 to 1.4)<br>Favours wheelchair skills training		
	**GRADE**<br>Very low certainty<br>⊕◯◯◯	**Risk of bias**<br>Serious	**Inconsistency**<br>Very serious	**Imprecision**<br>Very serious	**Indirectness**<br>Serious	**Publication bias**<br>Serious

Study or Subgroup	Experimental Mean	Experimental SD	Experimental Total	Control Mean	Control SD	Control Total	Weight	Std. Mean Difference IV, Random, 95% CI
Kirby_2016	87.3	12.8	47	87.9	10	49	31.8%	-0.05 [-0.45, 0.35]
Rice et al, 2013	-0.87	0.0735	6	-1.1	0.09	9	13.8%	2.58 [1.09, 4.07]
Worobey et al, 2016	87	13.4	36	81.4	11.3	43	31.0%	0.45 [0.00, 0.90]
Yeo et al, 2018	42.47	7.73	13	34.55	9.04	11	23.4%	0.92 [0.06, 1.77]
**Total (95% CI)**			**102**			**112**	**100.0%**	**0.69 [-0.02, 1.40]**

Heterogeneity: Tau² = 0.37; Chi² = 14.33, df = 3 (P = 0.002); I² = 79%
Test for overall effect: Z = 1.92 (P = 0.06)

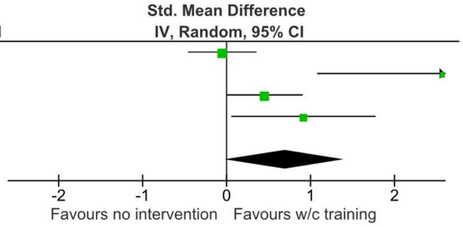

MANUAL WHEELCHAIR SKILLS TRAINING FOR WHEELCHAIR SKILLS: GRADE Evidence to Decision						
**PROBLEM**	No	Probably no	Probably yes	**Yes**		Don't know
**DESIRABLE EFFECTS**	Trivial	**Small**	Moderate	Large		Don't know
**UNDESIRABLE EFFECTS**	Large	Moderate	Small	Trivial		**Don't know**
**CERTAINTY OF EVIDENCE**	**Very low**	Low	Moderate	High		No included studies
**HOW MUCH PEOPLE VALUE THE MAIN OUTCOME**	Important uncertainty or variability	Possibly important uncertainty or variability	**Probably no important uncertainty or variability**	No important uncertainty or variability		

MANUAL WHEELCHAIR SKILLS TRAINING FOR WHEELCHAIR SKILLS: GRADE Evidence to Decision						
**BALANCE OF EFFECTS**	Favours the Control	Probably favours the Control	Does not favour either the intervention (I) or the comparison (C)	Probably favours the I	**Favours the I**	Don't know
**RESOURCES REQUIRED**	Large costs	Moderate costs	**Negligible costs and savings**	Moderate savings	Large savings	Don't know
**CERTAINTY OF EVIDENCE OF REQUIRED RESOURCES**	Very low	Low	Moderate	High		**No included studies**
**COST EFFECTIVENESS**	Favours the comparison	Probably favours the comparison	Does not favour either the intervention or the comparison	Probably favours the intervention	Favours the intervention	**No included studies**
**EQUITY**	Reduced	Probably reduced	**Probably no impact**	Probably increased	Increased	Don't know
**ACCEPTABILITY**	No	Probably no	Probably yes	**Yes**		Don't know
**FEASIBILITY**	No	Probably no	Probably yes	**Yes**		Don't know

MANUAL WHEELCHAIR SKILLS TRAINING FOR WHEELCHAIR SKILLS: Randomised Controlled Trial Details

STUDY	COMPARISON	DOSAGE/DETAILS	PARTICIPANTS	N (RX/C)	OUTCOME	ROB 2 PEDRO
**KIRBY 2016**	Wheelchair skills training V Educational Control	Intervention: Five individual training sessions<br><br>Control: Five education sessions	People with SCI living in community	47/49	Wheelchair skills test	Some concerns about Risk of Bias PEDro = 7/10
**RICE 2013**	Wheelchair skills training V No Intervention	3 visits of real time feedback from a Smart wheel while pushing	People with SCI living in community SCI<2 years	6/9	Stroke frequency	Some concerns about Risk of Bias PEDro = 4/10
**WOROBEY 2016**	Wheelchair skills training V Control	Between 2-8, 60–80-minute group training sessions	People with SCI living in community	36/43	Wheelchair skills test	High Risk of Bias PEDro = 7
**YEO 2018**	Wheelchair skills training V No Intervention	1 hour per day, 3 days/week for 8 weeks	People with tetraplegia living in community	13/11	Wheelchair skills test	Some concerns about Risk of Bias PEDro = 4

Virtual reality sitting training (v no intervention) on ability to sit in people with SCI

**P**	People with SCI	**Evidence recommendation**<br>◯ Weak for (95%)		**Weak evidence** recommendation **FOR**<br>Virtual Reality sitting training may be provided to improve the ability in sitting in people with SCI.		
**I**	Virtual reality (VR) sitting training					
**C**	No intervention	**Consensus-based opinion statement**<br>◯ No opinion statements				
**O**	Ability to sit					
	**SUMMARY**	1 RCT[28]		Mean difference (95% CI): Seated reach in mm<br>63 (38 to 89)<br>Favours VR sitting training		
	**GRADE**<br>Very low certainty<br>⊕◯◯◯	**Risk of bias**<br>Serious	**Inconsistency**<br>Serious	**Imprecision**<br>Serious	**Indirectness**<br>Serious	**Publication bias**<br>Serious

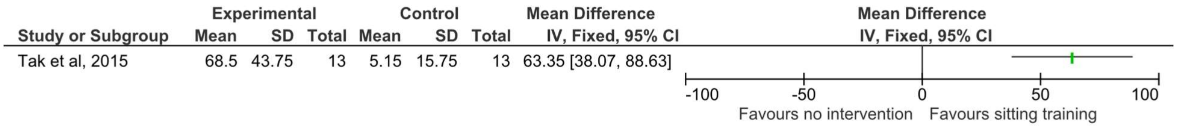

VIRTUAL REALITY SITTING TRAINING FOR ABILITY TO SIT: GRADE Evidence to Decision						
**PROBLEM**	No	Probably no	Probably yes	**Yes**		Don't know
**DESIRABLE EFFECTS**	Trivial	Small	**Moderate**	Large		Don't know
**UNDESIRABLE EFFECTS**	Large	Moderate	Small	Trivial		**Don't know**
**CERTAINTY OF EVIDENCE**	**Very low**	Low	Moderate	High		No included studies
**HOW MUCH PEOPLE VALUE THE MAIN OUTCOME**	Important uncertainty or variability	Possibly important uncertainty or variability	**Probably no important uncertainty or variability**	No important uncertainty or variability		
**BALANCE OF EFFECTS**	Favours the Control	Probably favours the Control	Does not favour either the intervention (I) or the comparison (C)	Probably favours the I	**Favours the I**	Don't know
**RESOURCES REQUIRED**	Large costs	**Moderate costs**	Negligible costs and savings	Moderate savings	Large savings	Don't know
**CERTAINTY OF EVIDENCE OF REQUIRED RESOURCES**	Very low	Low	Moderate	High		**No included studies**

VIRTUAL REALITY SITTING TRAINING FOR ABILITY TO SIT: GRADE Evidence to Decision						
**COST EFFECTIVENESS**	Favours the comparison	Probably favours the comparison	Does not favour either the intervention or the comparison	Probably favours the intervention	Favours the intervention	**No included studies**
**EQUITY**	Reduced	**Probably reduced**	Probably no impact	Probably increased	Increased	Don't know
**ACCEPTABILITY**	No	Probably no	**Probably yes**	Yes		Don't know
**FEASIBILITY**	No	Probably no	**Probably yes**	Yes		Don't know

VIRTUAL REALITY SITTING TRAINING FOR ABILITY TO SIT: Randomised Controlled Trial Details

STUDY	COMPARISON	DOSAGE/DETAILS	PARTICIPANTS	N (RX/C)	OUTCOME	ROB 2 PEDRO
**TAK 2015**	Game based virtual reality sitting training (plus usual care) V Usual care	6 weeks, 30 minutes ×3 sessions per week of Nintendo Wii-based VR balance training	AIS A or B SCI (cervical and thoracic)	13/13	Modified functional reach test (front)	Some concerns of Risk of Bias PEDro = 7/10

CONSENSUS-BASED OPINION STATEMENTS

	Walking training (v no intervention) on ability to walk in people who have lower limb motor function		
**P**	People with SCI who have lower limb motor function	**Evidence recommendation**<br>○ No evidence recommendation<br>Reason: No RCTs	**Strong opinion statement <u>FOR</u>**<br>Walking training should be provided to people with SCI who have lower limb motor function.<br><br>Walking training can include:<br>• Overground gait training (100%)<br>• Treadmill gait training (with and without body weight support) (100%)<br>• Treadmill gait training with electrical stimulation (+/- body weight support) (100%)<br>• Overground gait training and electrical stimulation (100%)<br>• Robotic overground gait training (92%)<br>• Robotic treadmill gait training (75%)<br>• Conventional therapy (package of interventions including gait training) (85%)<br>• Gait training with orthotics (100%)
**I**	Walking training		
**C**	No intervention	**Consensus-based opinion statement**<br>● Strong for (75% - 100%)	
**O**	Walking ability		

Conventional therapy (package of interventions including gait training) vs treadmill gait training (with or without body weight support) to improve walking in people with SCI and motor function in the lower limbs

**P**	People with SCI and motor function in the lower limbs	**Evidence recommendation**<br>○ No evidence recommendation<br>Reason: No recommendation due to insufficient or inconclusive evidence.	**Strong opinion statement FOR**<br>Conventional therapy (package of interventions that includes gait training) should be provided (in favour of treadmill gait training with or without body weight support) to improve walking in people with SCI.
**I**	Conventional therapy (package of interventions that includes gait training)		
**C**	Treadmill gait training (with or without body weight support)	**Consensus-based opinion statement**<br>● Strong for (96%)	
**O**	Walking ability		

**SUMMARY**	4 RCTS[29-32]	Mean difference (95% CI): Walking speed in m/s<br>0.08 (-0.12 to 0.27)<br>Favours conventional therapy (package of therapies including gait training)

**GRADE**	**Risk of bias**	**Inconsistency**	**Imprecision**	**Indirectness**	**Publication bias**
Very low certainty ⊕○○○	Very serious	No serious	Serious	No serious	Serious

Study or Subgroup	Experimental Mean	Experimental SD	Experimental Total	Control Mean	Control SD	Control Total	Weight	Mean Difference IV, Fixed, 95% CI
Alexeeva et al, 2011	0.46	0.4	9	0.51	0.36	12	35.0%	-0.05 [-0.38, 0.28]
Lucareli et al, 2011	1.25	0.41	12	0.98	0.65	12	20.3%	0.27 [-0.16, 0.70]
Piira et al, 2019b	-4.9	0.2864	7	-5	0.2864	7	42.7%	0.10 [-0.20, 0.40]
Sadeghi et al, 2015	0.175131	1.457726	10	0.233318	1.443001	7	2.0%	-0.06 [-1.46, 1.34]
**Total (95% CI)**			**38**			**38**	**100.0%**	**0.08 [-0.12, 0.27]**

Heterogeneity: Chi² = 1.38, df = 3 (P = 0.71); I² = 0%
Test for overall effect: Z = 0.79 (P = 0.43)

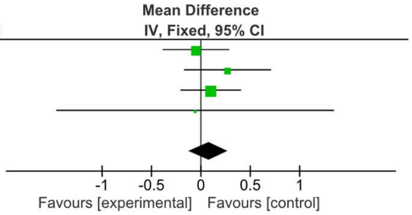

CONVENTIONAL THERAPY V TREADMILL ON ABILITY TO WALK: GRADE Evidence to Decision						
**PROBLEM**	No	Probably no	Probably yes	**Yes**		Don't know
**DESIRABLE EFFECTS**	Trivial	Small	Moderate	Large		**Don't know**
**UNDESIRABLE EFFECTS**	Large	Moderate	Small	Trivial		**Don't know**
**CERTAINTY OF EVIDENCE**	**Very low**	**Low**	Moderate	High		No included studies
**HOW MUCH PEOPLE VALUE THE MAIN OUTCOME**	Important uncertainty or variability	Possibly important uncertainty or variability	**Probably no important uncertainty or variability**	No important uncertainty or variability		
**BALANCE OF EFFECTS**	Favours the Control	Probably favours the Control	Does not favour either the intervention (I) or the comparison (C)	**Probably favours the I**	Favours the I	Don't know

CONVENTIONAL THERAPY V TREADMILL ON ABILITY TO WALK: GRADE Evidence to Decision						
RESOURCES REQUIRED	Large costs	Moderate costs	**Negligible costs and savings**	Moderate savings	Large savings	Don't know
CERTAINTY OF EVIDENCE OF REQUIRED RESOURCES	Very low	Low	Moderate	High		**No included studies**
COST EFFECTIVENESS	Favours the comparison	Probably favours the comparison	Does not favour either the intervention or the comparison	Probably favours the intervention	Favours the intervention	**No included studies**
EQUITY	Reduced	Probably reduced	**Probably no impact**	Probably increased	Increased	Don't know
ACCEPTABILITY	No	Probably no	Probably yes	**Yes**		Don't know
FEASIBILITY	No	Probably no	Probably yes	**Yes**		Don't know

CONVENTIONAL THERAPY V TREADMILL ON ABILITY TO WALK: Randomised Controlled Trial Details

STUDY	COMPARISON	DOSAGE/DETAILS	PARTICIPANTS	N (RX/C)	OUTCOME	ROB 2 PEDRO
**ALEXEEVA 2011**	Conventional therapy V Body weight support treadmill training (BWSTT)	BWSTT: 30% BWS 60 mins of training, 3 x per week for 13 weeks	AIS C and D SCI	9/12	Walking speed m/s	Some Concerns of Risk of Bias PEDro = 7/10
**LUCARELI 2011**	Conventional therapy V BWSTT	BWSTT: 30 mins of training, 2 x per week for 4 months (total 30 sessions)	AIS C and D SCI	12/12	Walking speed m/s	Some Concerns of Risk of Bias PEDro = 6/10
**PIIRA 2019**	Conventional therapy V BWSTT	BWSTT: 2 daily sessions, 90 minutes per day, 5 days per week over 12 weeks	AIS C and D SCI	7/7	Walking speed m/s	Some Concerns of Risk of Bias PEDro = 7/10
**SADEGHI 2015**	Conventional therapy V BWSTT	BWSTT: 60 min per session, 4 x per week for 12 weeks	AIS B and C SCI	10/7	Walking speed m/s	High Risk of Bias PEDro = 6/10

	Power wheelchair skills training (v no intervention) on power wheelchair skills in people with SCI who are dependent on a power wheelchair for mobility		
**P**	People with SCI who are dependent on a power wheelchair for mobility	**Evidence recommendation**<br>○ No evidence recommendation<br>Reason: No RCTs	**Strong opinion statement <u>FOR</u>**<br>Power wheelchair skills training should be provided to improve the ability to use a power wheelchair in people with SCI who are dependent on a power wheelchair for mobility.
**I**	Power wheelchair skills training		
**C**	No intervention	**Consensus-based opinion statement**<br>● Strong for (100%)	
**O**	Power wheelchair mobility		

	Bed mobility (v no intervention) on ability to move in bed in people with SCI		
**P**	People with SCI	**Evidence recommendation**<br>○ No evidence recommendation<br>Reason: No RCTs	**Strong opinion statement <u>FOR</u>**<br>Bed mobility training should be provided to improve the ability to move in bed in people with SCI.<br><br>Clinical note: This statement includes rolling and moving from supine to sitting for people with SCI that have sufficient muscle strength to actively participate in bed mobility training.
**I**	Bed mobility training		
**C**	No intervention	**Consensus-based opinion statement**<br>● Strong for (100%)	
**O**	Ability to move in bed		

	Sitting training (v no intervention) on ability to sit in people with SCI and motor function in the lower limbs		
**P**	People with SCI and motor function in the lower limbs	**Evidence recommendation**<br>○ No evidence recommendation<br>Reason: No RCTs	**Strong opinion statement <u>FOR</u>**<br>Sitting balance training should be provided to improve the ability to sit in people with SCI and motor function in the lower limbs.
**I**	Sitting balance training		
**C**	No intervention	**Consensus-based opinion statement**<br>● Strong for (78%)	
**O**	Ability to sit		

Sitting training (v no intervention) on ability to sit in people with SCI and paralysis of the lower limbs/trunk

**P**	People with SCI and paralysis of the lower limbs/trunk.	**Evidence recommendation**<br>◯ No evidence recommendation<br>Reason: No recommendation due to insufficient or inconclusive evidence.	**Strong opinion statement FOR**<br><br>Sitting balance training should be provided to improve the ability in sitting in people with SCI and paralysis of the lower limbs/trunk.
**I**	Sitting balance training		
**C**	No intervention	**Consensus-based opinion statement**<br>● Strong for (82%)	
**O**	Ability to sit		
	**SUMMARY**	2 RCTS[33-34]	Mean difference (95% CI): Reach distance in mm<br>22 (-60 to 104)<br>Favours sitting training

GRADE	Risk of bias	Inconsistency	Imprecision	Indirectness	Publication bias
Very low certainty ⊕◯◯◯	No serious	Very serious	Very serious	Serious	Serious

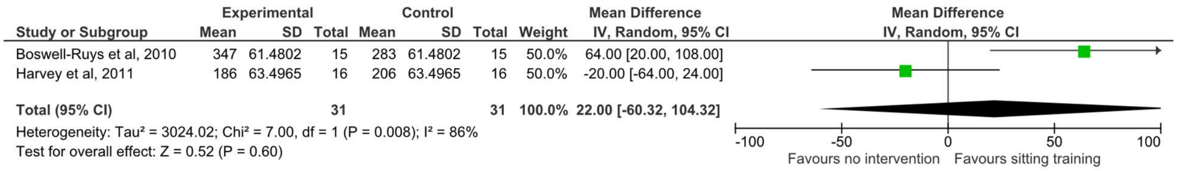

SITTING TRAINING ON ABILITY TO SIT: GRADE Evidence to Decision						
**PROBLEM**	No	Probably no	Probably yes	**Yes**		Don't know
**DESIRABLE EFFECTS**	Trivial	Small	Moderate	Large		**Don't know**
**UNDESIRABLE EFFECTS**	Large	Moderate	Small	Trivial		**Don't know**
**CERTAINTY OF EVIDENCE**	**Very low**	**Low**	Moderate	High		No included studies
**HOW MUCH PEOPLE VALUE THE MAIN OUTCOME**	Important uncertainty or variability	Possibly important uncertainty or variability	**Probably no important uncertainty or variability**	No important uncertainty or variability		
**BALANCE OF EFFECTS**	Favours the Control	Probably favours the Control	Does not favour either the intervention (I) or the comparison (C)	**Probably favours the I**	Favours the I	Don't know
**RESOURCES REQUIRED**	Large costs	Moderate costs	**Negligible costs and savings**	Moderate savings	Large savings	Don't know
**CERTAINTY OF EVIDENCE OF REQUIRED RESOURCES**	Very low	Low	Moderate	High		**No included studies**

SITTING TRAINING ON ABILITY TO SIT: GRADE Evidence to Decision

**COST EFFECTIVENESS**	Favours the comparison	Probably favours the comparison	Does not favour either the intervention or the comparison	Probably favours the intervention	Favours the intervention	**No included studies**
**EQUITY**	Reduced	Probably reduced	**Probably no impact**	Probably increased	Increased	Don't know
**ACCEPTABILITY**	No	Probably no	Probably yes	**Yes**		Don't know
**FEASIBILITY**	No	Probably no	Probably yes	**Yes**		Don't know

SITTING TRAINING ON ABILITY TO SIT: Randomised Controlled Trial Details

STUDY	COMPARISON	DOSAGE/DETAILS	PARTICIPANTS	N (RX/C)	OUTCOME	ROB 2 PEDRO
**BOSWELL-RUYS 2010**	Sitting training V No intervention	1 hour of training, 3 x per week for 6 weeks	T1-T12 with chronic SCI	15/15	Maximal balance range test (mm)	Some concerns of Risk of Bias PEDro = 8/10
**HARVEY 2011**	Sitting training (plus usual care) V Usual care	3 additional 30-minute sessions per week of motor retraining for sitting	Acute paraplegia	16/16	Maximal lean test (mm)	Some concerns of Risk of Bias PEDro = 8/10

Transfer training v no intervention on ability to transfer in people with SCI

**P**	People with SCI	**Evidence recommendation**<br>◯ No evidence recommendation<br>Reason: No RCTs	**Strong opinion statement FOR**<br>Transfer training should be provided to improve the ability to transfer in people with SCI.<br><br>Clinical note: This statement includes transfers for people with SCI that have sufficient muscle strength to actively participate in transfer training. The method of transfer will depend on muscle strength.
**I**	Transfer training		
**C**	No intervention	**Consensus-based opinion statement**<br>● Strong for (100%)	
**O**	Ability to transfer		

<table>
<tr><th colspan="4">Vertical transfer training (v no intervention) on ability to vertically transfer in people with SCI who are wheelchair dependent</th></tr>
<tr><td>P</td><td>People with SCI that are wheelchair dependent</td><td rowspan="2">Evidence recommendation
No evidence recommendation
Reason: No RCTs</td><td rowspan="4">Strong opinion statement <u>FOR</u>
Vertical transfer training should be provided to improve the ability to vertically transfer in people with SCI who are wheelchair dependent.

Clinical note: This statement includes floor to wheelchair and wheelchair to floor transfers for people with sufficient strength to participate in vertical transfer training.</td></tr>
<tr><td>I</td><td>Vertical transfer training</td></tr>
<tr><td>C</td><td>No intervention</td><td rowspan="2">Consensus-based opinion statement
Strong for (81%)</td></tr>
<tr><td>O</td><td>Ability to transfer</td></tr>
</table>

<table>
<tr><th colspan="4">Sit to stand training (v no intervention) on ability to move from sit to stand in people with SCI and motor function in the lower limbs</th></tr>
<tr><td>P</td><td>People with SCI and motor function in the lower limbs</td><td rowspan="2">Evidence recommendation
No evidence recommendation
Reason: No RCTs</td><td rowspan="4">Strong opinion statement <u>FOR</u>
Sit to stand training should be provided to improve the ability to move from sit to stand in people with SCI and motor function in the lower limbs.

Clinical note: This statement includes standing up from sitting for people with SCI that have sufficient muscle strength in the lower limbs to actively participate in sit to stand training.</td></tr>
<tr><td>I</td><td>Sit to stand training</td></tr>
<tr><td>C</td><td>No intervention</td><td rowspan="2">Consensus-based opinion statement
Strong for (89%)</td></tr>
<tr><td>O</td><td>Ability to move into standing</td></tr>
</table>

<table>
<tr><th colspan="4">Standing training (v no intervention) on ability to stand in people with SCI and motor function in the lower limbs</th></tr>
<tr><td>P</td><td>People with SCI and motor function in the lower limbs</td><td rowspan="2">Evidence recommendation
No evidence recommendation
Reason: No RCTs</td><td rowspan="4">Strong opinion statement <u>FOR</u>
Standing training should be provided to improve the ability to stand in people with SCI (who have motor function in the lower limbs).

Clinical note: This statement includes standing training for people with SCI that have sufficient muscle strength to actively participate in standing training.</td></tr>
<tr><td>I</td><td>Standing training</td></tr>
<tr><td>C</td><td>No intervention</td><td rowspan="2">Consensus-based opinion statement
Strong for (95%)</td></tr>
<tr><td>O</td><td>Ability to stand</td></tr>
</table>

	Stair training (v no intervention) on ability to climb stairs in people with SCI and motor function in the lower limbs		
**P**	People with SCI and motor function in the lower limbs	**Evidence recommendation**<br>○ No evidence recommendation<br>Reason: No RCTs	**Strong opinion statement FOR**<br>Stair training should be provided to improve the ability to climb stairs in people with SCI who can walk.<br><br>Clinical note: This statement includes ascending and descending stairs for people with SCI (and upright mobility) that have sufficient muscle strength and/or appropriate assistive devices to actively participate in stair training.
**I**	Stair training		
**C**	No intervention	**Consensus-based opinion statement**<br>● Strong for (85%)	
**O**	Ability to climb stairs		

	Upper limb and hand function training (v no intervention) on upper limb and hand function in people with tetraplegia					
**P**	People with tetraplegia	**Evidence recommendation**<br>○ No evidence recommendation<br>Reason: No recommendation due to insufficient or inconclusive evidence.		**Strong opinion statement FOR**<br>Upper limb function training should be provided to improve hand function in people with tetraplegia.		
**I**	Upper limb function training					
**C**	No intervention	**Consensus-based opinion statement**<br>● Strong for (92%)				
**O**	Upper limb and hand function					
	**SUMMARY**	1 RCT[35]		Mean difference (95% CI): Hand function in points on Jebsen Hand Function test<br>128 (60 to 196)<br>Favours hand training		
	**GRADE**<br>Very low certainty<br>⊕○○○	**Risk of bias**<br>Very serious	**Inconsistency**<br>Serious	**Imprecision**<br>No serious	**Indirectness**<br>Serious	**Publication bias**<br>Serious

Study or Subgroup	Experimental Mean	Experimental SD	Experimental Total	Control Mean	Control SD	Control Total	Mean Difference IV, Fixed, 95% CI
Beekhuizen et al, 2008	128.89	84.3359	6	0.74	3.8947	6	128.15 [60.60, 195.70]

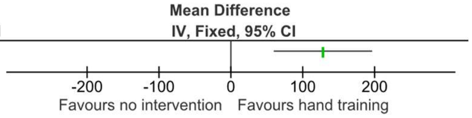

UPPER LIMB and HAND TRAINING ON UPPER LIMB and HAND FUNCTION: GRADE Evidence to Decision						
**PROBLEM**	No	Probably no	Probably yes	**Yes**		Don't know
**DESIRABLE EFFECTS**	Trivial	**Small**	Moderate	Large		Don't know
**UNDESIRABLE EFFECTS**	Large	Moderate	Small	Trivial		**Don't know**
**CERTAINTY OF EVIDENCE**	**Very low**	Low	Moderate	High		No included studies
**HOW MUCH PEOPLE VALUE THE MAIN OUTCOME**	Important uncertainty or variability	Possibly important uncertainty or variability	**Probably no important uncertainty or variability**	No important uncertainty or variability		
**BALANCE OF EFFECTS**	Favours the Control	Probably favours the Control	Does not favour either the intervention (I) or the comparison (C)	**Probably favours the I**	Favours the I	Don't know
**RESOURCES REQUIRED**	Large costs	Moderate costs	**Negligible costs and savings**	Moderate savings	Large savings	Don't know
**CERTAINTY OF EVIDENCE OF REQUIRED RESOURCES**	Very low	Low	Moderate	High		**No included studies**
**COST EFFECTIVENESS**	Favours the comparison	Probably favours the comparison	Does not favour either the intervention or the comparison	Probably favours the intervention	Favours the intervention	**No included studies**
**EQUITY**	Reduced	Probably reduced	**Probably no impact**	Probably increased	Increased	Don't know
**ACCEPTABILITY**	No	Probably no	Probably yes	**Yes**		Don't know
**FEASIBILITY**	No	Probably no	Probably yes	**Yes**		Don't know

UPPER LIMB and HAND TRAINING ON UPPER LIMB and HAND FUNCTION: Randomised Controlled Trial Details

STUDY	COMPARISON	DOSAGE/DETAILS	PARTICIPANTS	N (RX/C)	OUTCOME	ROB 2 PEDRO
**BEEKHUIZEN 2008**	Hand training v No intervention	2 hours of massed practice hand training 5 x per week for 3 weeks	C4-C7 tetraplegia	6/6	Jebsen Hand Function test (points)	High Risk of Bias PEDro = 4/10

<table>
<tr><th colspan="4">Robotic Upper limb training (v no intervention) on upper limb function in people with tetraplegia</th></tr>
<tr><td>P</td><td>People with tetraplegia</td><td rowspan="2">Evidence recommendation
○ No evidence recommendation
Reason: No RCTs</td><td rowspan="4">Strong opinion statement <u>FOR</u>
Robotic upper limb training should be provided to improve upper limb function in people with tetraplegia.</td></tr>
<tr><td>I</td><td>Robotic upper limb training</td></tr>
<tr><td>C</td><td>No intervention</td><td rowspan="2">Consensus-based opinion statement
● Strong for (89%)</td></tr>
<tr><td>O</td><td>Upper limb function</td></tr>
</table>

<table>
<tr><th colspan="4">Tenodesis splinting (v no intervention) on a tenodesis grip in people with C6 or C7 tetraplegia</th></tr>
<tr><td>P</td><td>People with C6 and C7 tetraplegia</td><td rowspan="2">Evidence recommendation
○ No evidence recommendation
Reason: No RCTs</td><td rowspan="4">Weak opinion statement <u>FOR</u>
Tenodesis splinting may be provided to improve a tenodesis grip in people with C6 and C7 tetraplegia.

Clinical note: Tenodesis grip is contraindicated for people for people who may be candidates for upper limb nerve and tendon transfer surgery</td></tr>
<tr><td>I</td><td>Tenodesis splinting</td></tr>
<tr><td>C</td><td>No intervention</td><td rowspan="2">Consensus-based opinion statement
● Weak for (100%)</td></tr>
<tr><td>O</td><td>Tenodesis grip</td></tr>
</table>

	**Upper limb and hand function training and FES (v no intervention) on hand function in people with tetraplegia**		
**P**	People with tetraplegia	**Evidence recommendation**<br>◯ No evidence recommendation<br>Reason: No recommendation due to insufficient or inconclusive evidence.	**Weak opinion statement <u>FOR</u>**<br>Upper limb and hand function training and FES may be provided to improve hand function in people with tetraplegia.
**I**	Upper limb and hand function training and FES		
**C**	No intervention	**Consensus-based opinion statement**<br>● Weak for (96%)	
**O**	Upper limb and hand function		
	**SUMMARY**	2 RCTs[36-37]	Standardised mean difference (95% CI)<br>0.2 (-0.3 to 0.8)<br>Favours hand training with FES

**GRADE**<br>Very low certainty<br>⊕◯◯◯	**Risk of bias**<br>Very serious	**Inconsistency**<br>No serious	**Imprecision**<br>Very serious	**Indirectness**<br>No serious	**Publication bias**<br>Serious

Study or Subgroup	Experimental Mean	Experimental SD	Experimental Total	Control Mean	Control SD	Control Total	Weight	Std. Mean Difference IV, Random, 95% CI
Harvey et al, 2017	34.1	10.3434	35	33.2	10.3434	31	73.5%	0.09 [-0.40, 0.57]
Hoffman et al, 2013	411.1	297.4	10	235.2	174.2	9	26.5%	0.68 [-0.25, 1.61]
**Total (95% CI)**			**45**			**40**	**100.0%**	**0.24 [-0.27, 0.76]**

Heterogeneity: Tau² = 0.03; Chi² = 1.23, df = 1 (P = 0.27); I² = 18%
Test for overall effect: Z = 0.93 (P = 0.35)

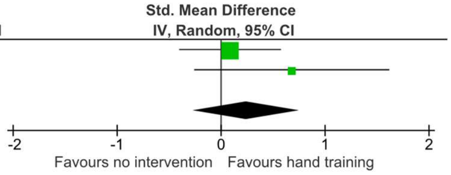

**UPPER LIMB AND HAND FUNCTION TRAINING PLUS FES ON UPPER LIMB AND HAND FUNCTION: GRADE Evidence to Decision**						
**PROBLEM**	No	Probably no	Probably yes	**Yes**		Don't know
**DESIRABLE EFFECTS**	Trivial	Small	Moderate	Large		**Don't know**
**UNDESIRABLE EFFECTS**	Large	Moderate	Small	Trivial		**Don't know**
**CERTAINTY OF EVIDENCE**	**Very low**	Low	Moderate	High		No included studies
**HOW MUCH PEOPLE VALUE THE MAIN OUTCOME**	Important uncertainty or variability	Possibly important uncertainty or variability	**Probably no important uncertainty or variability**	No important uncertainty or variability		
**BALANCE OF EFFECTS**	Favours the Control	Probably favours the Control	Does not favour either the intervention (I) or the comparison (C)	**Probably favours the I**	Favours the I	Don't know

**UPPER LIMB AND HAND FUNCTION TRAINING PLUS FES ON UPPER LIMB AND HAND FUNCTION: GRADE Evidence to Decision**						
**RESOURCES REQUIRED**	Large costs	Moderate costs	**Negligible costs and savings**	Moderate savings	Large savings	Don't know
**CERTAINTY OF EVIDENCE OF REQUIRED RESOURCES**	Very low	Low	Moderate	High		**No included studies**
**COST EFFECTIVENESS**	Favours the comparison	Probably favours the comparison	Does not favour either the intervention or the comparison	Probably favours the intervention	Favours the intervention	**No included studies**
**EQUITY**	Reduced	Probably reduced	**Probably no impact**	Probably increased	Increased	Don't know
**ACCEPTABILITY**	No	Probably no	Probably yes	**Yes**		Don't know
**FEASIBILITY**	No	Probably no	Probably yes	**Yes**		Don't know

UPPER LIMB AND HAND FUNCTION TRAINING PLUS FES ON UPPER LIMB AND HAND FUNCTION: Randomised Controlled Trial Details

STUDY	COMPARISON	DOSAGE/DETAILS	PARTICIPANTS	N (RX/C)	OUTCOME	ROB 2 PEDRO
**HARVEY 2017**	Hand training plus FES (plus usual care) V Usual care	1 hour per day, 5 days per week for 8 weeks.	C2-T1 tetraplegia	35/31	Action Research Arm Test (ARAT)	Low Risk of Bias PEDro = 8/10
**HOFFMAN 2013**	Hand training plus FES V No intervention	5 x per week, 2 hours per day, for 3 weeks.	Chronic tetraplegia	10/9	Jebsen Hand function test	High Risk of Bias PEDro = 3/10

Upper limb virtual reality (v no intervention) on upper limb function in people with tetraplegia

**P**	People with tetraplegia	**Evidence recommendation**<br>◯ No evidence recommendation<br>Reason: No recommendation due to insufficient or inconclusive evidence.	**Weak opinion statement FOR**<br>Upper limb virtual reality training may be provided to improve UL function in people with tetraplegia.
**I**	Upper limb virtual reality training may be provided to improve UL function in people with tetraplegia.		
**C**	No intervention	**Consensus-based opinion statement**<br>● Weak for (100%)	
**O**	Upper limb function		
	**SUMMARY**	3 RCTs[38-40]	Standardised mean difference (95% CI)<br>0.7 (-1.6 to 0.2)<br>Favours no intervention

GRADE	Risk of bias	Inconsistency	Imprecision	Indirectness	Publication bias
Very low certainty ⊕◯◯◯	Very serious	Serious	Very serious	No serious	Serious

Study or Subgroup	Experimental Mean	Experimental SD	Experimental Total	Control Mean	Control SD	Control Total	Weight	Std. Mean Difference IV, Random, 95% CI
Dimbwadyo-Terrer et al, 2016	3.87	1.3998	15	6.07	1.3998	16	34.4%	-1.53 [-2.34, -0.72]
Lim et al, 2020	76.22	16.25	10	74.51	19.44	10	33.1%	0.09 [-0.79, 0.97]
Prasad et al, 2018	2	6.363961	11	5.34099	4.065279	9	32.5%	-0.59 [-1.49, 0.32]
**Total (95% CI)**			**36**			**35**	**100.0%**	**-0.69 [-1.63, 0.26]**

Heterogeneity: Tau² = 0.50; Chi² = 7.18, df = 2 (P = 0.03); I² = 72%
Test for overall effect: Z = 1.42 (P = 0.15)

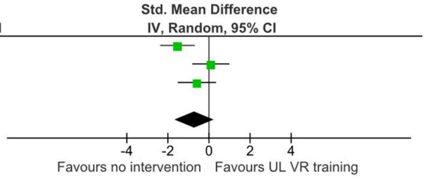

UPPER LIMB VIRTUAL REALITY TRAINING ON UPPER LIMB FUNCTION: GRADE Evidence to Decision						
**PROBLEM**	No	Probably no	Probably yes	**Yes**		Don't know
**DESIRABLE EFFECTS**	Trivial	Small	Moderate	Large		**Don't know**
**UNDESIRABLE EFFECTS**	Large	Moderate	Small	Trivial		**Don't know**
**CERTAINTY OF EVIDENCE**	**Very low**	Low	Moderate	High		No included studies
**HOW MUCH PEOPLE VALUE THE MAIN OUTCOME**	Important uncertainty or variability	Possibly important uncertainty or variability	**Probably no important uncertainty or variability**	No important uncertainty or variability		
**BALANCE OF EFFECTS**	Favours the Control	**Probably favours the Control**	Does not favour either the intervention (I) or the comparison (C)	Probably favours the I	Favours the I	Don't know

UPPER LIMB VIRTUAL REALITY TRAINING ON UPPER LIMB FUNCTION: GRADE Evidence to Decision						
**RESOURCES REQUIRED**	Large costs	Moderate costs	**Negligible costs and savings**	Moderate savings	Large savings	Don't know
**CERTAINTY OF EVIDENCE OF REQUIRED RESOURCES**	Very low	Low	Moderate	High		**No included studies**
**COST EFFECTIVENESS**	Favours the comparison	Probably favours the comparison	Does not favour either the intervention or the comparison	Probably favours the intervention	Favours the intervention	**No included studies**
**EQUITY**	Reduced	Probably reduced	**Probably no impact**	Probably increased	Increased	Don't know
**ACCEPTABILITY**	No	Probably no	**Probably yes**	Yes		Don't know
**FEASIBILITY**	No	Probably no	**Probably yes**	Yes		Don't know

UPPER LIMB VIRTUAL REALITY TRAINING ON UPPER LIMB FUNCTION: Randomised Controlled Trial Details

STUDY	COMPARISON	DOSAGE/DETAILS	PARTICIPANTS	N (RX/C)	OUTCOME	ROB 2 PEDRO
**DIMBWADYO-TERRER 2016**	Virtual reality UL training (plus usual care) V Usual care	15 sessions with Toyra(®) virtual reality system for 5 30 minutes per day, 3 days/week for 5 weeks	Complete tetraplegia	15/16	SCIM (self-care sub-score)	Some Concerns of Risk Bias PEDro = 6/10
**LIM 2020**	Virtual reality (plus usual care) V Usual care	30 minutes of VR training and 30 minutes of conventional therapy per day, 4 x per week for 4 weeks	C4-C6 tetraplegia	10/10	SCIM	High Risk of Bias PEDro = 5/10
**PRASAD 2018**	Virtual reality UL training (plus usual care) V Usual care	3 x per week for 4 weeks	tetraplegia	11/9	Box and block test	High Risk of Bias PEDro = 6/10

Overground gait training v Robotic gait training to improve walking in people with SCI and motor function in the lower limbs

**P**	People with SCI and motor function in the lower limbs	**Evidence recommendation**<br>○ No evidence recommendation<br>Reason: No recommendation due to insufficient or inconclusive evidence.	**Weak opinion statement FOR**<br>Overground gait training may be provided (in favour of robotic gait training) to improve walking in people with SCI.<br><br>Clinical note: Robotic gait training includes the use of devices such as the Lokomat (with and without electrical stimulation) and exoskeletons.<br>.
**I**	Overground gait training		
**C**	Robotic gait training	**Consensus-based opinion statement**<br>● Weak for (85%)	
**O**	Ability to walk		
	**SUMMARY**	3 RCTs[41-43]	Mean difference (95% CI): Walking ability: WISCI points<br>3 (-1 to 7)<br>Favours robotic gait training

**GRADE**	**Risk of bias**	**Inconsistency**	**Imprecision**	**Indirectness**	**Publication bias**
Very low certainty ⊕○○○	Serious	Very serious	Serious	No serious	Serious

Study or Subgroup	Experimental Mean	Experimental SD	Experimental Total	Control Mean	Control SD	Control Total	Weight	Mean Difference IV, Random, 95% CI
Alcobendas-Maestro et al, 2012	16	7.777778	37	9	5.925926	38	30.6%	7.00 [3.86, 10.14]
Esclarin-Ruz et al, 2014	12.9724	4.9485	41	10.92	4.7652	42	34.3%	2.05 [-0.04, 4.14]
Hornby et al, 2005	5.782123	2.178771	10	6.564246	2.011173	10	35.1%	-0.78 [-2.62, 1.06]
**Total (95% CI)**			**88**			**90**	**100.0%**	**2.57 [-1.39, 6.54]**

Heterogeneity: $Tau^2 = 10.78$; $Chi^2 = 18.01$, $df = 2$ ($P = 0.0001$); $I^2 = 89\%$
Test for overall effect: $Z = 1.27$ ($P = 0.20$)

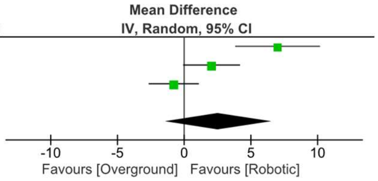

**OVERGROUND GAIT TRAINING V ROBOTIC GAIT TRAINING ON WALKING: GRADE Evidence to Decision**						
**PROBLEM**	No	Probably no	Probably yes	**Yes**		Don't know
**DESIRABLE EFFECTS**	Trivial	Small	Moderate	Large		**Don't know**
**UNDESIRABLE EFFECTS**	Large	Moderate	Small	Trivial		**Don't know**
**CERTAINTY OF EVIDENCE**	**Very low**	Low	Moderate	High		No included studies
**HOW MUCH PEOPLE VALUE THE MAIN OUTCOME**	Important uncertainty or variability	Possibly important uncertainty or variability	**Probably no important uncertainty or variability**	No important uncertainty or variability		
**BALANCE OF EFFECTS**	Favours the Control	Probably favours the Control	Does not favour either the intervention (I) or the comparison (C)	**Probably favours Robotic training**	Favours the I	Don't know
**RESOURCES REQUIRED**	Large costs Robotic training	Moderate costs	**Negligible costs and savings of overground walking training**	Moderate savings	Large savings	Don't know

OVERGROUND GAIT TRAINING V ROBOTIC GAIT TRAINING ON WALKING: GRADE Evidence to Decision						
CERTAINTY OF EVIDENCE OF REQUIRED RESOURCES	Very low	Low	Moderate	High		**No included studies**
COST EFFECTIVENESS	Favours the comparison	Probably favours the comparison	Does not favour either the intervention or the comparison	Probably favours the intervention	Favours the intervention	**No included studies**
EQUITY	Reduced	Probably reduced	**Probably no impact for overground walking training**	Probably increased	Increased	Don't know
ACCEPTABILITY	No	Probably no	Probably yes	**Yes for overground walking training**		Don't know
FEASIBILITY	No	Probably no	Probably yes	**Yes for overground walking training**		Don't know

OVERGROUND GAIT TRAINING V ROBOTIC GAIT TRAINING ON WALKING: Randomised Controlled Trial Details

STUDY	COMPARISON	DOSAGE/DETAILS	PARTICIPANTS	N (RX/C)	OUTCOME	ROB 2 PEDRO
**ALCOBENDAS-MAESTRO 2012**	Overground gait training V Robotic Gait training	Intervention: 40 sessions of overground gait training<br><br>Comparison: 40 sessions of lokomat	C2 to T12 AIS C and D SCI	37/38	Walking Index for SCI	Some Concerns of Risk of Bias PEDro = 8/10
**ESCLARIN-RUZ 2014**	Overground gait training V Robotic Gait training	Intervention: Overground training 60 minute, 5 days/week for 8 weeks<br><br>Comparison: Lokomat 60 minutes 5 days/week for 8 weeks	AIS C or D SCI	41/42	Walking Index For SCI	Some Concerns of Risk of Bias PEDro = 8/10
**HORNBY 2005**	Overground gait training V Robotic Gait training	Intervention: Overground gait training 3 x 30mins per week for 8 weeks<br><br>Comparison: Robotic gait training 3 x 30 mins per week for 8 weeks	T10 to L4 AIS B,C,D SCI	10/10	Walking index for SCI	Some Concerns of Risk of Bias PEDro = 3/10

Overground gait training vs Treadmill gait training (with or without body weight support) to improve walking in people with SCI and motor function in the lower limbs

**P**	People with SCI and motor function in the lower limbs	**Evidence recommendation**<br>◯ No evidence recommendation<br>Reason: No recommendation due to insufficient or inconclusive evidence.	**Weak opinion statement FOR**<br>Overground gait training may be provided (in favour of treadmill gait training with or without body weight support) to improve walking in people with SCI.
**I**	Overground gait training		
**C**	Treadmill gait training (with and without body weight support)	**Consensus-based opinion statement**<br>● Weak for (79%)	
**O**	Ability to walk		

**SUMMARY**	4 RCTs[44-47]	Standardised Mean Difference (95% CI)<br>0 (-0.3 to 0.4)	

**GRADE**	**Risk of bias**	**Inconsistency**	**Imprecision**	**Indirectness**	**Publication bias**
Very low certainty<br>⊕◯◯◯	Serious	Serious	Very serious	Serious	Serious

	Experimental			Control				Std. Mean Difference	Std. Mean Difference
Study or Subgroup	Mean	SD	Total	Mean	SD	Total	Weight	IV, Fixed, 95% CI	IV, Fixed, 95% CI
Dobkin et al, 2006	1.1	0.81	35	1	1.08	33	56.6%	0.10 [-0.37, 0.58]	
Hornby et al, 2005	5.224719	1.938202	10	6.657303	2.078652	10	15.6%	-0.68 [-1.59, 0.23]	
Senthilvelkumar et al, 2015	2.4	4.8681	7	2.1	4.8681	7	11.7%	0.06 [-0.99, 1.11]	
Yang et al, 2014	0.0657	0.0813	10	0.03	0.061	10	16.1%	0.48 [-0.42, 1.37]	
**Total (95% CI)**			**62**			**60**	**100.0%**	**0.04 [-0.32, 0.39]**	

Heterogeneity: Chi² = 3.42, df = 3 (P = 0.33); I² = 12%
Test for overall effect: Z = 0.20 (P = 0.84)

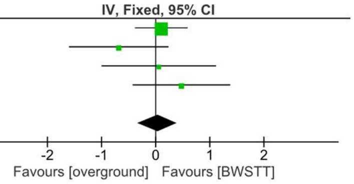

OVERGROUND GAIT TRAINING V TREADMILL GAIT TRAINING ON WALKING: GRADE Evidence to Decision

**PROBLEM**	No	Probably no	Probably yes	**Yes**		Don't know
**DESIRABLE EFFECTS**	Trivial	Small	Moderate	Large		**Don't know**
**UNDESIRABLE EFFECTS**	Large	Moderate	Small	Trivial		**Don't know**
**CERTAINTY OF EVIDENCE**	**Very low**	Low	Moderate	High		No included studies
**HOW MUCH PEOPLE VALUE THE MAIN OUTCOME**	Important uncertainty or variability	Possibly important uncertainty or variability	**Probably no important uncertainty or variability**	No important uncertainty or variability		
**BALANCE OF EFFECTS**	Favours the Control	Probably favours the Control	**Does not favour either the intervention (I) or the comparison (C)**	Probably favours the I	Favours the I	Don't know
**RESOURCES REQUIRED**	Large costs	Moderate costs	**Negligible costs and savings**	Moderate savings	Large savings	Don't know

OVERGROUND GAIT TRAINING V TREADMILL GAIT TRAINING ON WALKING: GRADE Evidence to Decision						
**CERTAINTY OF EVIDENCE OF REQUIRED RESOURCES**	Very low	Low	Moderate	High		**No included studies**
**COST EFFECTIVENESS**	Favours the comparison	Probably favours the comparison	Does not favour either the intervention or the comparison	Probably favours the intervention	Favours the intervention	**No included studies**
**EQUITY**	Reduced	Probably reduced	**Probably no impact for overground walking training**	Probably increased	Increased	Don't know
**ACCEPTABILITY**	No	Probably no	Probably yes	**Yes for overground walking training**		Don't know
**FEASIBILITY**	No	Probably no	Probably yes	**Yes for overground walking training**		Don't know

OVERGROUND GAIT TRAINING V TREADMILL GAIT TRAINING ON WALKING: Randomised Controlled Trial Details

STUDY	COMPARISON	DOSAGE/DETAILS	PARTICIPANTS	N (RX/C)	OUTCOME	ROB 2 PEDRO
**DOBKIN 2006**	Overground gait training V Treadmill gait training	Intervention: Mobility training one hour per day, 5 x per week for 12 weeks<br><br>Comparison: Treadmill training plus mobility training one hour per day, 5 x per week for 12 weeks	People with SCI	35/33	Walking speed m/s	High Risk of Bias PEDro = 7/10
**HORNBY 2005**	Overground gait training V Treadmill gait training	Intervention: Overground gait training 3 x 30mins per week for 8 weeks<br><br>Comparison: BWSTT 3 x 30 mins per week for 8 weeks	T10 to L4 AIS B,C,D SCI	10/10	Walking Index for SCI	Some Concerns of Risk of Bias PEDro = 3/10
**SENTHILVELKUMAR 2015**	Overground gait training V Body weight support treadmill gait training	Intervention: Body weight support overground training, 30 mins 5 x per week for 8 weeks<br><br>Comparison: treadmill training, 30 mins 5 x per week for 8 weeks	People with SCI	7/7	Walking Index for SCI	Some Concerns of Risk of Bias PEDro = 7/10

OVERGROUND GAIT TRAINING V TREADMILL GAIT TRAINING ON WALKING: Randomised Controlled Trial Details

**YANG 2014**	Overground gait training<br>V<br>Treadmill gait training	Intervention: Overground training one hour per day, 5 x per week for 2 months<br><br>Comparison: BWSTT one hour per day, 5 times x week for 2 months	People with SCI	10/10	Walking speed m/s	High Risk of Bias<br>PEDro = 6/10

Treadmill gait training (with or without body weight support) vs Robotic gait training to improve walking in people with SCI and motor function in the lower limbs

**P**	People with SCI and motor function in the lower limbs	**Evidence recommendation**<br>◯ No evidence recommendation<br>Reason: No recommendation due to insufficient or inconclusive evidence.	**Weak opinion statement FOR**<br>Treadmill gait training with or without body weight support may be provided (in favour of robotic gait training) to improve walking in people with SCI.
**I**	Treadmill gait training (with and without body weight support)		
**C**	Robotic gait training	**Consensus-based opinion statement**<br>● Weak for (89%)	
**O**	Ability to walk		
	**SUMMARY**	2 RCTs[43,47]	Standardised Mean Difference (95% CI)<br>-0.2 (-0.8 to 0.4)<br>Favours treadmill gait training (with or without body weight support)

**GRADE**	**Risk of bias**	**Inconsistency**	**Imprecision**	**Indirectness**	**Publication bias**
Very low certainty<br>⊕◯◯◯	Serious	Serious	Very serious	Serious	Very serious

Study or Subgroup	Experimental Mean	Experimental SD	Experimental Total	Control Mean	Control SD	Control Total	Weight	Std. Mean Difference IV, Fixed, 95% CI
Field_Fote et al, 2011	0.01	0.05	14	0.04	0.07	17	60.0%	-0.47 [-1.19, 0.25]
Hornby et al, 2005	5.758427	2.219101	10	5.252809	1.882022	10	40.0%	0.24 [-0.64, 1.12]
**Total (95% CI)**			**24**			**27**	**100.0%**	**-0.19 [-0.75, 0.37]**

Heterogeneity: $Chi^2 = 1.49$, df = 1 (P = 0.22); $I^2 = 33\%$
Test for overall effect: Z = 0.67 (P = 0.50)

Std. Mean Difference
IV, Fixed, 95% CI

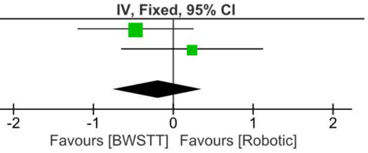

**TREADMILL GAIT TRAINING V ROBOTIC GAIT TRAINING ON WALKING: GRADE Evidence to Decision**						
**PROBLEM**	No	Probably no	Probably yes	**Yes**		Don't know
**DESIRABLE EFFECTS**	Trivial	Small	Moderate	Large		**Don't know**
**UNDESIRABLE EFFECTS**	Large	Moderate	Small	Trivial		**Don't know**

TREADMILL GAIT TRAINING V ROBOTIC GAIT TRAINING ON WALKING: GRADE Evidence to Decision						
CERTAINTY OF EVIDENCE	**Very low**	Low	Moderate	High		No included studies
HOW MUCH PEOPLE VALUE THE MAIN OUTCOME	Important uncertainty or variability	Possibly important uncertainty or variability	**Probably no important uncertainty or variability**	No important uncertainty or variability		
BALANCE OF EFFECTS	Favours the Control	**Probably favours treadmill gait training**	Does not favour either the intervention (I) or the comparison (C)	Probably favours the I	Favours the I	Don't know
Resources required	**Large costs for both**	Moderate costs	Negligible costs and savings	Moderate savings	Large savings	Don't know
CERTAINTY OF EVIDENCE OF REQUIRED RESOURCES	Very low	Low	Moderate	High		**No included studies**
COST EFFECTIVENESS	Favours the comparison	Probably favours the comparison	Does not favour either the intervention or the comparison	Probably favours the intervention	Favours the intervention	**No included studies**
EQUITY	**Reduced**	Probably reduced	Probably no impact	Probably increased	Increased	Don't know
ACCEPTABILITY	No	Probably no	Probably yes	**Yes For treadmill gait training**		Don't know
FEASIBILITY	No	Probably no	Probably yes	**Yes For treadmill gait training**		Don't know

TREADMILL GAIT TRAINING V ROBOTIC GAIT TRAINING ON WALKING: Randomised Controlled Trial Details

STUDY	COMPARISON	DOSAGE/DETAILS	PARTICIPANTS	N (RX/C)	OUTCOME	ROB 2 PEDRO
**FIELD-FOTE 2011**	Treadmill gait training V Robotic gait training	Intervention: BWSTT with manual assistance 5 days per week for 12 weeks Comparison: Robotic gait training 5 days per week for 12 weeks	Chronic SCI	14/17	Speed m/s	High Risk of Bias PEDro = 6/10
**HORNBY 2005**	Treadmill gait training V Robotic gait training	Intervention: BWSTT 3 x 30mins per week for 8 weeks<br><br>Comparison: Robotic BWSTT 3 x 30mins per week for 8 weeks	T10 to L4 AIS B,C,D SCI	10/10	Walking index for SCI	Some Concerns of Risk of Bias PEDro = 3/10

	**Hydrotherapy as an adjunct to land based therapy vs no intervention to improve function in people with SCI.**		
**P**	People with SCI	**Evidence recommendation**<br>◯ No evidence recommendation<br>Reason: No RCTs	**Weak opinion statement FOR**<br><br>Hydrotherapy may be provided as an adjunct to land based therapy to improve function in people with SCI.<br><br>Clinical note; Hydrotherapy should not be chosen over land therapy but can be used as a useful adjunct.
**I**	Hydrotherapy as an adjunct to land based therapy		
**C**	No intervention	**Consensus-based opinion statement**<br>● Weak for (95%)	
**O**	Function		

	**Gait training (BWS or robotics) vs no intervention to improve functional walking in people with SCI that have no motor function in the lower limbs**		
**P**	People with SCI that have no motor function in the lower limbs.	**Evidence recommendation**<br>◯ No evidence recommendation<br>Reason: No RCTs	**Strong opinion statement AGAINST**<br><br>Gait training (BWS or robotics) should not be provided to improve functional walking in people with SCI that have no motor function in the lower limbs.
**I**	Gait training		
**C**	No intervention	**Consensus-based opinion statement**<br>● Strong Against (86%)	
**O**	Ability to walk		

	**Gait training (orthotics) vs no intervention to improve functional walking in people with SCI that have no motor function in the lower limbs**		
**P**	People with SCI that have no motor function in the lower limbs.	**Evidence recommendation**<br>◯ No evidence recommendation<br>Reason: No RCTs	**Strong opinion statement AGAINST**<br><br>Gait training (orthotics) should not be provided to improve functional walking in people with SCI that have no motor function in the lower limbs.<br><br>Clinical note: Bilateral knee ankle foot orthosis (KAFOs) or hip knee ankle foot orthosis (HKAFOs) may be useful in certain circumstances for goals such as standing or fitness.
**I**	Gait training		
**C**	No intervention	**Consensus-based opinion statement**<br>● Strong Against (89%)	
**O**	Ability to walk		

6. Physiotherapy interventions for pain

EVIDENCE RECOMMENDATIONS

	TENS (v no intervention) on pain in people with SCI					
**P**	People with SCI	**Evidence recommendation**<br>◯ Weak for (95%)		**Weak evidence recommendation <u>FOR</u>**<br>Evidence recommendation: TENS may be provided for pain in people with SCI.		
**I**	TENS					
**C**	No intervention	**Consensus-based opinion statement**<br>◯ No opinion statements				
**O**	Pain					
	**SUMMARY**	2 RCTs[48-49]		Mean difference (95% CI): Pain (VAS)<br>-2 (-3 to -1)<br>Favours TENS		
	**GRADE**<br>Very low certainty<br>⊕◯◯◯	**Risk of bias**<br>Very serious	**Inconsistency**<br>Serious	**Imprecision**<br>No serious	**Indirectness**<br>Serious	**Publication bias**<br>Serious

Study or Subgroup	Experimental Mean	Experimental SD	Experimental Total	Control Mean	Control SD	Control Total	Weight	Mean Difference IV, Random, 95% CI
Bi et al, 2015	2.14	0.91	24	3.87	1.45	24	63.8%	-1.73 [-2.41, -1.05]
Celik et al 2013	3.88	2.5	17	6.77	1.42	16	36.2%	-2.89 [-4.27, -1.51]
**Total (95% CI)**			**41**			**40**	**100.0%**	**-2.15 [-3.24, -1.06]**

Heterogeneity: Tau² = 0.36; Chi² = 2.19, df = 1 (P = 0.14); I² = 54%
Test for overall effect: Z = 3.86 (P = 0.0001)

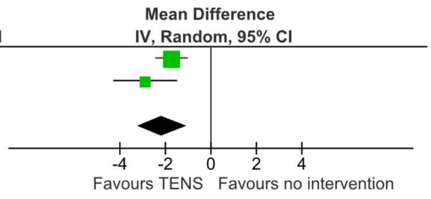

TENS FOR PAIN: GRADE Evidence to Decision						
PROBLEM	No	Probably no	Probably yes	**Yes**		Don't know
DESIRABLE EFFECTS	Trivial	Small	**Moderate**	Large		Don't know
UNDESIRABLE EFFECTS	Large	Moderate	Small	Trivial		**Don't know**
CERTAINTY OF EVIDENCE	**Very low**	Low	Moderate	High		No included studies
HOW MUCH PEOPLE VALUE THE MAIN OUTCOME	Important uncertainty or variability	Possibly important uncertainty or variability	**Probably no important uncertainty or variability**	No important uncertainty or variability		

TENS FOR PAIN: GRADE Evidence to Decision						
BALANCE OF EFFECTS	Favours the Control	Probably favours the Control	Does not favour either the intervention (I) or the comparison (C)	Probably favours the I	**Favours the I**	Don't know
RESOURCES REQUIRED	Large costs	Moderate costs	**Negligible costs and savings**	Moderate savings	Large savings	Don't know
CERTAINTY OF EVIDENCE OF REQUIRED RESOURCES	Very low	Low	Moderate	High		**No included studies**
COST EFFECTIVENESS	Favours the comparison	Probably favours the comparison	Does not favour either the intervention or the comparison	Probably favours the intervention	Favours the intervention	**No included studies**
EQUITY	Reduced	Probably reduced	**Probably no impact**	Probably increased	Increased	Don't know
ACCEPTABILITY	No	Probably no	Probably yes	**Yes**		Don't know
FEASIBILITY	No	Probably no	Probably yes	**Yes**		Don't know

TENS FOR PAIN: Randomised Controlled Trial Details

STUDY	COMPARISON	DOSAGE/DETAILS	PARTICIPANTS	N (RX/C)	OUTCOME	ROB 2 PEDRO
**BI 2015**	TENS V Sham TENS	TENS 20 minutes, 3 x per week for 12 weeks	People with SCI	24/24	Visual Analogue Pain scale	Some Concerns of Risk of Bias PEDro = 7/10
**CELIK 2013**	TENS V Sham TENS	30 mins per day for 10 days	People with SCI	17/16	Visual Analogue Pain scale	High Risk of Bias PEDro = 4/10

CONSENSUS-BASED OPINION STATEMENTS

	Education to avoid overuse and trauma (v no intervention) on shoulder pain in people with SCI		
**P**	People with SCI	**Evidence recommendation**<br>○ No evidence recommendation<br>Reason: No RCTs	**Strong opinion statement FOR**<br>Education to avoid shoulder overuse and trauma should be provided to prevent and treat shoulder pain in people with SCI.<br>Clinical note: Education could include education about strategies to avoid shoulder overuse and trauma.
**I**	Education to avoid shoulder overuse and trauma		
**C**	No intervention	**Consensus-based opinion statement**<br>● Strong for (100%)	
**O**	Shoulder pain		

Shoulder exercises (v no intervention) on shoulder pain (treatment) in people with SCI

	PICO	Evidence recommendation	Strong opinion statement **FOR**
**P**	People with SCI who have shoulder pain	**Evidence recommendation**<br>○ No evidence recommendation<br>Reason: No recommendation due to insufficient or inconclusive evidence.	Shoulder exercises should be provided to treat shoulder pain in people with SCI.
**I**	Shoulder exercises		
**C**	No intervention	**Consensus-based opinion statement**<br>● Strong for (81%)	
**O**	Shoulder pain		

**SUMMARY**	5 RCTs[50-54]	Mean difference (95% CI): Pain on Wheelchair Users Shoulder Pain Index in points<br>Consider studies independently. Unable to pool I^2=76%

**GRADE**	**Risk of bias**	**Inconsistency**	**Imprecision**	**Indirectness**	**Publication bias**
Very low certainty ⊕○○○	Very serious	Very serious	Serious	No serious	Serious

Study or Subgroup	Experimental Mean	Experimental SD	Experimental Total	Control Mean	Control SD	Control Total	Mean Difference IV, Random, 95% CI
Cardenas et al, 2019	-53.6	30.49	11	-39.23	28.62	8	-14.37 [-41.16, 12.42]
Curtis et al, 2011	-14	20.1	17	-11.8	17.6	18	-2.20 [-14.75, 10.35]
Dondal et al, 2015	-57.7	13.68	15	-69.9	19.82	15	12.20 [0.01, 24.39]
Mulroy et al, 2011	-14.9	14	26	-45.6	38.2	32	30.70 [16.41, 44.99]
Nightingale et al, 2018	-13	13	13	-14	15	8	1.00 [-11.57, 13.57]

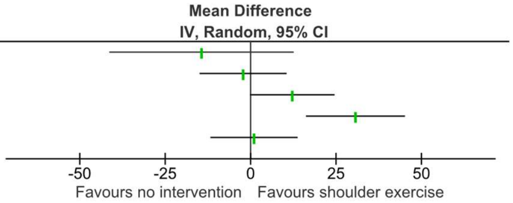

**SHOULDER EXERCISES FOR SHOULDER PAIN: GRADE Evidence to Decision**						
**PROBLEM**	No	Probably no	Probably yes	**Yes**		Don't know
**DESIRABLE EFFECTS**	Trivial	Small	Moderate	Large		**Don't know**
**UNDESIRABLE EFFECTS**	Large	Moderate	Small	Trivial		**Don't know**
**CERTAINTY OF EVIDENCE**	**Very low**	Low	Moderate	High		No included studies
**HOW MUCH PEOPLE VALUE THE MAIN OUTCOME**	Important uncertainty or variability	Possibly important uncertainty or variability	**Probably no important uncertainty or variability**	No important uncertainty or variability		
**BALANCE OF EFFECTS**	Favours the Control	Probably favours the Control	Does not favour either the intervention (I) or the comparison (C)	**Probably favours the I**	Favours the I	Don't know
**RESOURCES REQUIRED**	Large costs	Moderate costs	**Negligible costs and savings**	Moderate savings	Large savings	Don't know

SHOULDER EXERCISES FOR SHOULDER PAIN: GRADE Evidence to Decision						
**CERTAINTY OF EVIDENCE OF REQUIRED RESOURCES**	Very low	Low	Moderate	High		**No included studies**
**COST EFFECTIVENESS**	Favours the comparison	Probably favours the comparison	Does not favour either the intervention or the comparison	Probably favours the intervention	Favours the intervention	**No included studies**
**EQUITY**	Reduced	Probably reduced	**Probably no impact**	Probably increased	Increased	Don't know
**ACCEPTABILITY**	No	Probably no	Probably yes	**Yes**		Don't know
**FEASIBILITY**	No	Probably no	Probably yes	**Yes**		Don't know

SHOULDER EXERCISES FOR SHOULDER PAIN Randomised Controlled Trial Details

STUDY	COMPARISON	DOSAGE/DETAILS	PARTICIPANTS	N (RX/C)	OUTCOME	ROB 2 PEDRO
**CARDENAS 2020**	Shoulder home exercise programme V Control (education)	3 x per week for 12 weeks based on Mulroy 2011	People with SCI and shoulder pain (all levels)	11/8	Wheelchair users Shoulder Pain Index (WUSPI)	High Risk of Bias PEDro = 6/10
**CURTIS 2011**	Shoulder exercises V No intervention	5 exercises twice daily for 6 months	C6 or lower SCI	17/18	WUSPI	Some Concerns of Risk of Bias PEDro = 4/10
**DONDAL 2015**	Shoulder strengthening and stretching exercises V No intervention	3 x per week for 4 weeks	Below T1 SCI	15/15	WUSPI	High Risk of Bias PEDro = 6/10
**MULROY 2011**	Home-based shoulder exercise programme V Control (education)	3 x per week for 12 weeks	T2 to T7 SCI with shoulder pain	26/32	WUSPI	High Risk of Bias PEDro = 7/10
**NIGHTINGALE 2018**	Arm cranking (portable desktop ergometer) V No intervention	4 x per week for 6 weeks (moderate intensity)	Below T2 SCI	13/8	WUSPI	Some Concerns of Risk of Bias PEDro = 5/10

Shoulder Positioning (v no intervention) on shoulder pain (prevention) in people with SCI

**P**	People with SCI at risk of shoulder pain	**Evidence recommendation**<br>◯ No evidence recommendation<br>Reason: No recommendation due to insufficient or inconclusive evidence.	**Strong opinion statement FOR**<br>Shoulder positioning in a lengthened position should be provided to prevent shoulder pain in people with tetraplegia.<br>.
**I**	Shoulder positioning		
**C**	No intervention	**Consensus-based opinion statement**<br>● Weak for (100%)	
**O**	Shoulder pain		
	**SUMMARY**	1 RCT[55]	Mean difference (95% CI): Pain on Visual Analogue Scale<br>-0.4 (-1.6 to 0.9)<br>Favours positioning

GRADE	Risk of bias	Inconsistency	Imprecision	Indirectness	Publication bias
Very low certainty ⊕◯◯◯	Very serious	Serious	No serious	Serious	Serious

Study or Subgroup	Experimental Mean	Experimental SD	Experimental Total	Control Mean	Control SD	Control Total	Mean Difference IV, Fixed, 95% CI
Crowe et al, 2000	1.7	2	18	2.1	2	21	-0.40 [-1.66, 0.86]

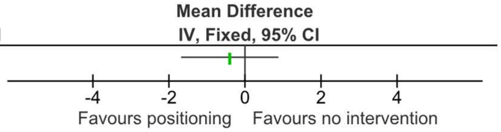

SHOULDER POSITIONING ON SHOULDER PAIN: GRADE Evidence to Decision						
**PROBLEM**	No	Probably no	Probably yes	**Yes**		Don't know
**DESIRABLE EFFECTS**	Trivial	Small	Moderate	Large		**Don't know**
**UNDESIRABLE EFFECTS**	Large	Moderate	Small	Trivial		**Don't know**
**CERTAINTY OF EVIDENCE**	**Very low**	Low	Moderate	High		No included studies
**HOW MUCH PEOPLE VALUE THE MAIN OUTCOME**	Important uncertainty or variability	Possibly important uncertainty or variability	**Probably no important uncertainty or variability**	No important uncertainty or variability		
**BALANCE OF EFFECTS**	Favours the Control	Probably favours the Control	Does not favour either the intervention (I) or the comparison (C)	**Probably favours the I**	Favours the I	Don't know
**RESOURCES REQUIRED**	Large costs	Moderate costs	**Negligible costs and savings**	Moderate savings	Large savings	Don't know
**CERTAINTY OF EVIDENCE OF REQUIRED RESOURCES**	Very low	Low	Moderate	High		**No included studies**

SHOULDER POSITIONING ON SHOULDER PAIN: GRADE Evidence to Decision						
COST EFFECTIVENESS	Favours the comparison	Probably favours the comparison	Does not favour either the intervention or the comparison	Probably favours the intervention	Favours the intervention	**No included studies**
EQUITY	Reduced	Probably reduced	**Probably no impact**	Probably increased	Increased	Don't know
ACCEPTABILITY	No	Probably no	Probably yes	**Yes**		Don't know
FEASIBILITY	No	Probably no	Probably yes	**Yes**		Don't know

SHOULDER POSITIONING ON SHOULDER PAIN: Randomised Controlled Trial Details

STUDY	COMPARISON	DOSAGE/DETAILS	PARTICIPANTS	N (RX/C)	OUTCOME	ROB 2 PEDRO
**CROWE 2000**	Positioning (and usual care) V Usual care alone	45 mins of positioning once daily on weekdays for the period of participants were in acute care facility.	C2-C7 tetraplegia	18/21	Visual Analogue Pain Scale	High Risk of Bias PEDro = 6/10

Massage (v no intervention) on pain in people with SCI

**P**	People with SCI	**Evidence recommendation**<br>◯ No evidence recommendation<br>Reason: No recommendation due to insufficient or inconclusive evidence.	**Weak opinion statement FOR**<br>Massage therapy may be provided to treat pain in people with SCI.
**I**	Massage therapy		
**C**	No intervention	**Consensus-based opinion statement**<br>● Weak for (96%)	
**O**	Pain		
	**SUMMARY**	2 RCTs[56-57]	Mean difference (95% CI)<br>0.1 (-0.4 to 0.5)<br>Favours no intervention

**GRADE**<br>Very low certainty<br>⊕◯◯◯	**Risk of bias**<br>Very serious	**Inconsistency**<br>No Serious	**Imprecision**<br>Serious	**Indirectness**<br>Serious	**Publication bias**<br>Serious	

Massage (v no intervention) on pain in people with SCI

Study or Subgroup	Experimental Mean	Experimental SD	Experimental Total	Control Mean	Control SD	Control Total	Weight	Std. Mean Difference IV, Fixed, 95% CI
Chase, 2013	4.03	2.04	20	3.91	2.4	20	50.0%	0.05 [-0.57, 0.67]
Lovas, 2017	9.1	8.1	20	8.6	8.9	20	50.0%	0.06 [-0.56, 0.68]
**Total (95% CI)**			**40**			**40**	**100.0%**	**0.06 [-0.38, 0.49]**

Heterogeneity: Chi² = 0.00, df = 1 (P = 0.99); I^2 = 0%
Test for overall effect: Z = 0.25 (P = 0.81)

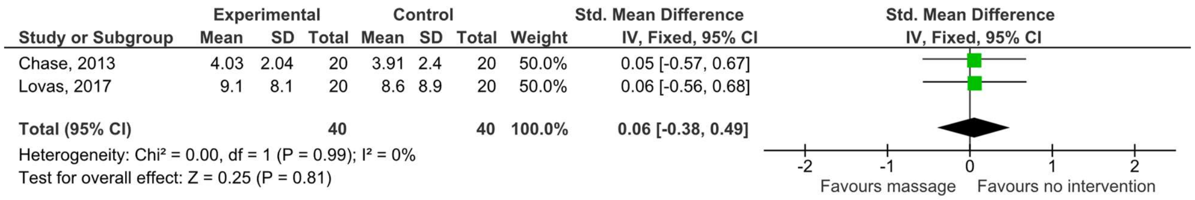

MASSAGE ON PAIN: GRADE Evidence to Decision						
PROBLEM	No	Probably no	Probably yes	**Yes**		Don't know
DESIRABLE EFFECTS	Trivial	Small	Moderate	Large		**Don't know**
UNDESIRABLE EFFECTS	Large	Moderate	Small	Trivial		**Don't know**
CERTAINTY OF EVIDENCE	**Very low**	Low	Moderate	High		No included studies
HOW MUCH PEOPLE VALUE THE MAIN OUTCOME	Important uncertainty or variability	Possibly important uncertainty or variability	**Probably no important uncertainty or variability**	No important uncertainty or variability		
BALANCE OF EFFECTS	Favours the Control	Probably favours the Control	**Does not favour either the intervention (I) or the comparison (C)**	Probably favours the I	Favours the I	Don't know
RESOURCES REQUIRED	Large costs	Moderate costs	**Negligible costs and savings**	Moderate savings	Large savings	Don't know
CERTAINTY OF EVIDENCE OF REQUIRED RESOURCES	Very low	Low	Moderate	High		**No included studies**
COST EFFECTIVENESS	Favours the comparison	Probably favours the comparison	Does not favour either the intervention or the comparison	Probably favours the intervention	Favours the intervention	**No included studies**
EQUITY	Reduced	Probably reduced	**Probably no impact**	Probably increased	Increased	Don't know
ACCEPTABILITY	No	Probably no	Probably yes	**Yes**		Don't know
FEASIBILITY	No	Probably no	**Probably yes**	Yes		Don't know

MASSAGE ON PAIN: Randomised Controlled Trial Details

STUDY	COMPARISON	DOSAGE/DETAILS	PARTICIPANTS	N (RX/C)	OUTCOME	ROB 2 PEDRO
**CHASE 2013**	Massage V No intervention	Six 20 min massage sessions over 2 weeks	People with complete and incomplete SCI	20/20	Shortform McGill Pain Questionnaire (SF-MPQ)	High Risk of Bias PEDro = 5/10

MASSAGE ON PAIN: Randomised Controlled Trial Details

**LOVAS 2017**	Massage<br>V<br>Guided imagery relaxation	1 x per week (30 mins) for 5 weeks	People with complete and incomplete SCI	20/20	Intensity on the Brief Pain Inventory	High Risk of Bias<br>PEDro = 4/10

<table>
<tr><th></th><th colspan="3">Passive movements (v no intervention) on shoulder pain</th></tr>
<tr><td>P</td><td>People with SCI at risk of shoulder pain</td><td rowspan="2">Evidence recommendation
◯ No evidence recommendation
Reason: No RCTs</td><td rowspan="4">No evidence recommendation or consensus-based opinion statement</td></tr>
<tr><td>I</td><td>Passive movements</td></tr>
<tr><td>C</td><td>No intervention</td><td rowspan="2">Consensus-based opinion statement
◯ No consensus statements
Reason: No consensus could be reached</td></tr>
<tr><td>O</td><td>Shoulder pain</td></tr>
</table>

7. Physiotherapy interventions for shoulder subluxation

CONSENSUS-BASED OPINION STATEMENTS

	Equipment to support the shoulder (v no intervention) on shoulder subluxation (prevention) in people with SCI at risk of shoulder subluxation		
P	People with SCI at risk of shoulder subluxation	**Evidence recommendation**<br>○ No evidence recommendation<br>Reason: No RCTs	**Strong opinion statement FOR**<br>Equipment to support the shoulder such as wheelchair armrests or shoulder support devices should be provided to prevent and treat shoulder subluxation.<br><br>Clinical note: Equipment to support the shoulder includes wheelchair armrests, pillows under the elbows or shoulder support braces.
I	Equipment to support the shoulder		
C	No intervention	**Consensus-based opinion statement**<br>● Strong for (83%)	
O	Shoulder subluxation		

	Neuromuscular electrical stimulation (v no intervention) on shoulder subluxation (prevention and treatment) in people with SCI at risk of shoulder subluxation		
P	People with SCI at risk of shoulder subluxation	**Evidence recommendation**<br>○ No evidence recommendation<br>Reason: No RCTs	**Weak opinion statement FOR**<br>Neuromuscular electrical stimulation of the shoulder may be provided to prevent and treat shoulder subluxation in people with SCI that are at risk of shoulder subluxation.<br><br>Clinical note: This statement applies to people with partial innervation to the shoulder following SCI.
I	Neuromuscular electrical stimulation of the shoulder		
C	No intervention	**Consensus-based opinion statement**<br>● Weak for (100%)	
O	Shoulder subluxation		

8. Physiotherapy interventions for joint mobility

EVIDENCE RECOMMENDATIONS

	**Long duration stretch (v no intervention) on joint mobility in people with SCI**					
**P**	People with SCI	**Evidence recommendation**<br>◯ Weak for (95%)		**Weak evidence** recommendation **FOR**<br>Long duration stretch may be provided to prevent and treat loss of joint mobility in people with SCI.		
**I**	Long duration stretch					
**C**	No intervention	**Consensus-based opinion statement**<br>◯ No opinion statements				
**O**	Joint mobility					
	**SUMMARY**	3 RCTs[58-60]		Mean difference (95% CI): Joint mobility in degrees<br>2 (-1 to 5) Favours long duration stretch<br>Favours stretch		
	**GRADE**<br>Very Low certainty<br>⊕◯◯◯	**Risk of bias**<br>Serious	**Inconsistency**<br>Serious	**Imprecision**<br>No serious	**Indirectness**<br>Serious	**Publication bias**<br>Serious

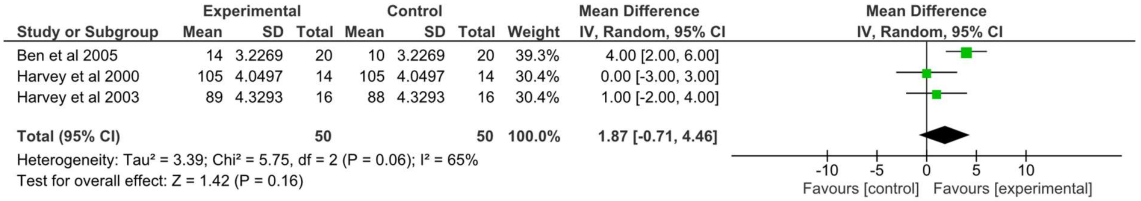

**LONG DURATION STRETCH ON JOINT MOBILITY: GRADE Evidence to Decision**						
**PROBLEM**	No	Probably no	Probably yes	**Yes**		Don't know
**DESIRABLE EFFECTS**	Trivial	**Small**	Moderate	Large		Don't know
**UNDESIRABLE EFFECTS**	Large	Moderate	Small	Trivial		**Don't know**
**CERTAINTY OF EVIDENCE**	**Very low**	Low	Moderate	High		No included studies
**HOW MUCH PEOPLE VALUE THE MAIN OUTCOME**	Important uncertainty or variability	Possibly important uncertainty or variability	**Probably no important uncertainty or variability**	No important uncertainty or variability		

LONG DURATION STRETCH ON JOINT MOBILITY: GRADE Evidence to Decision						
BALANCE OF EFFECTS	Favours the Control	Probably favours the Control	Does not favour either the intervention (I) or the comparison (C)	Probably favours the I	**Favours the I**	Don't know
RESOURCES REQUIRED	Large costs	Moderate costs	**Negligible costs and savings**	Moderate savings	Large savings	Don't know
CERTAINTY OF EVIDENCE OF REQUIRED RESOURCES	Very low	Low	Moderate	High		**No included studies**
COST EFFECTIVENESS	Favours the comparison	Probably favours the comparison	Does not favour either the intervention or the comparison	Probably favours the intervention	Favours the intervention	**No included studies**
EQUITY	Reduced	Probably reduced	**Probably no impact**	Probably increased	Increased	Don't know
ACCEPTABILITY	No	Probably no	Probably yes	**Yes**		Don't know
FEASIBILITY	No	Probably no	Probably yes	**Yes**		Don't know

LONG DURATION STRETCH ON JOINT MOBILITY: Randomised Controlled Trial Details

STUDY	COMPARISON	DOSAGE/DETAILS	PARTICIPANTS	N (RX/C)	OUTCOME	ROB 2 PEDRO
**BEN 2005**	Long duration stretch V No intervention	30 minutes, 3 x per week for 12 weeks of standing on TT	People with SCI and LL paralysis	20/20	Ankle mobility (degrees)	Some Concerns of Risk of Bias PEDro = 8/10
**HARVEY 2000**	Long duration stretch V No intervention	30 mins, 5 x per week for 4 weeks	People with SCI and LL paralysis	7/7	Ankle mobility (degrees)	Some Concerns of Risk of Bias PEDro = 8/10
**HARVEY 2003**	Long duration stretch V No intervention	30 mins, 5 x per week for 4 weeks	People with SCI and LL paralysis	16/16	Joint mobility/Hamstring length (degrees)	Some Concerns of Risk of Bias PEDro = 7/10

CONSENSUS-BASED OPINION STATEMENTS

	Passive standing (v no intervention) on joint mobility in people with SCI and paralysed lower limbs		
P	People with SCI	**Evidence recommendation**<br>◯ No evidence recommendation<br>Reason: No recommendation due to insufficient or inconclusive evidence.	**Weak opinion statement FOR**<br>Passive standing may be provided to prevent and treat loss of ROM in people with SCI and paralysed lower limbs.<br><br>Clinical note: Passive standing includes standing in frames, devices or on a tilt table.
I	Passive standing		
C	No intervention	**Consensus-based opinion statement**<br>● Weak for (90%)	
O	Joint mobility		
	**SUMMARY**	1 RCT[58]	Mean difference (95% CI): Joint mobility in degrees<br>4 (2 to 6)<br>Favours passive standing

**GRADE**	**Risk of bias**	**Inconsistency**	**Imprecision**	**Indirectness**	**Publication bias**
Very low certainty<br>⊕◯◯◯	Serious	Serious	No serious	Serious	Serious

Study or Subgroup	Experimental Mean	Experimental SD	Experimental Total	Control Mean	Control SD	Control Total	Mean Difference IV, Fixed, 95% CI
Ben et al 2005	14	3.2269	20	10	3.2269	20	4.00 [2.00, 6.00]

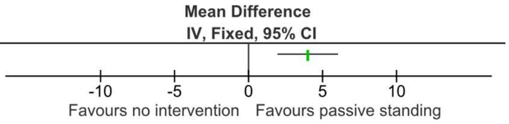

**PASSIVE STANIDNG ON JOINT MOBILITY: GRADE Evidence to Decision**						
**PROBLEM**	No	Probably no	Probably yes	**Yes**		Don't know
**DESIRABLE EFFECTS**	Trivial	**Small**	Moderate	Large		Don't know
**UNDESIRABLE EFFECTS**	Large	Moderate	Small	Trivial		**Don't know**
**CERTAINTY OF EVIDENCE**	**Very low**	Low	Moderate	High		No included studies
**HOW MUCH PEOPLE VALUE THE MAIN OUTCOME**	Important uncertainty or variability	Possibly important uncertainty or variability	**Probably no important uncertainty or variability**	No important uncertainty or variability		
**BALANCE OF EFFECTS**	Favours the Control	Probably favours the Control	Does not favour either the intervention (I) or the comparison (C)	Probably favours the I	**Favours the I**	Don't know
**RESOURCES REQUIRED**	Large costs	Moderate costs	**Negligible costs and savings**	Moderate savings	Large savings	Don't know

PASSIVE STANIDNG ON JOINT MOBILITY: GRADE Evidence to Decision						
CERTAINTY OF EVIDENCE OF REQUIRED RESOURCES	Very low	Low	Moderate	High		**No included studies**
COST EFFECTIVENESS	Favours the comparison	Probably favours the comparison	Does not favour either the intervention or the comparison	Probably favours the intervention	Favours the intervention	**No included studies**
EQUITY	Reduced	Probably reduced	**Probably no impact**	Probably increased	Increased	Don't know
ACCEPTABILITY	No	Probably no	Probably yes	**Yes**		Don't know
FEASIBILITY	No	Probably no	Probably yes	**Yes**		Don't know

PASSIVE STANIDNG ON JOINT MOBILITY: Randomised Controlled Trial Details

STUDY	COMPARISON	DOSAGE/DETAILS	PARTICIPANTS	N (RX/C)	OUTCOME	ROB 2 PEDRO
**BEN 2005**	Long duration stretch V No intervention	30 minutes, 3 x per week for 12 weeks of standing on tilt table	People with SCI and LL paralysis	20/20	Ankle mobility (degrees)	Some Concerns of Risk of Bias PEDro = 8/10

Active Assisted Exercise (v no intervention) on joint mobility (prevention) in people with SCI who are at risk of contracture

**P**	People with SCI at risk of contracture	**Evidence recommendation**<br>◯ No evidence recommendation<br>Reason: No RCTs	**Weak opinion statement FOR**<br>Active assisted exercises may be provided to prevent loss of joint mobility in people with SCI who are at risk of contracture.
**I**	Active assisted exercises		
**C**	No intervention	**Consensus-based opinion statement**<br>● Weak for (92%)	
**O**	Contracture		

	Active Assisted Exercise (v no intervention) on joint mobility (treatment) in people with SCI who are at risk of contracture		
**P**	People with SCI at risk of contracture	**Evidence recommendation**<br>No evidence recommendation<br>Reason: No RCTs	**Weak opinion statement FOR**<br>Active assisted exercises may be provided to treat loss of joint mobility in people with SCI
I	Active assisted exercises		
**C**	No intervention	**Consensus-based opinion statement**<br>Weak for (96%)	
**O**	Contracture		

	Serial casting (v no intervention) on joint mobility in people with SCI that have contracture		
**P**	People with SCI that have contracture	**Evidence recommendation**<br>No evidence recommendation<br>Reason: No RCTs	**Weak opinion statement FOR**<br>Serial casting may be provided to treat contracture in people with SCI.<br><br>Clinical note: Serial casting is only recommended if the contractures are impacting activity and participation. It can cause serious pressure injuries, particularly in those with spasticity, and/or impaired or absent sensation, so should only be administered by physiotherapists with experience in serial casting and with careful ongoing monitoring.
I	Serial casting		
**C**	No intervention	**Consensus-based opinion statement**<br>Weak for (79%)	
**O**	Contracture		

	Hand splinting versus no intervention on prevention of hand contractures in people with tetraplegia		
**P**	People with tetraplegia	**Evidence recommendation**<br>No evidence recommendation<br>Reason: No RCTs	**Weak opinion statement FOR**<br>Hand splinting may be provided to prevent hand contracture in people with tetraplegia who are at risk of contracture.
I	Hand splinting		
**C**	No intervention	**Consensus-based opinion statement**<br>Weak for (100%)	
**O**	Contracture		

	Hand splinting versus no intervention on treatment of hand contractures in people with tetraplegia		
**P**	People with tetraplegia	**Evidence recommendation**<br>◯ No evidence recommendation<br>Reason: No RCTs	**Weak opinion statement FOR**<br>Hand splinting may be provided to treat hand contracture in people with tetraplegia.
I	Hand splinting		
**C**	No intervention	**Consensus-based opinion statement**<br>● Weak for (92%)	
**O**	Contracture		

	Upper and lower limb splinting versus no intervention on prevention of contractures in people with SCI who are at risk of contracture		
**P**	People with SCI who are at risk of contracture	**Evidence recommendation**<br>◯ No evidence recommendation<br>Reason: No RCTs	**Weak opinion statement FOR**<br>Upper and lower limb splinting may be provided to prevent joint contracture in people with SCI who are at risk of contracture.<br><br>Clinical note: Splinting can cause serious pressure injuries, particularly in those with spasticity, and/or impaired or absent sensation, so should only be administered by physiotherapists with experience in splinting and with careful ongoing monitoring.
I	Upper and lower limb splinting		
**C**	No intervention	**Consensus-based opinion statement**<br>● Weak for (100%)	
**O**	Contracture		

	Passive range of motion exercises (v no intervention) on joint mobility in people with SCI					
P	People with SCI	Evidence recommendation<br>◯ No evidence recommendation<br>Reason: No recommendation due to insufficient or inconclusive evidence.		**Weak opinion statement FOR**<br>Passive range of motion exercises may be provided to prevent and treat loss of joint mobility in people with SCI.		
I	Passive range of motion exercises					
C	No intervention	**Consensus-based opinion statement**<br>● Weak for (100%)				
O	Joint mobility					
	**SUMMARY**	1 RCT[61]		Mean difference (95% CI): Joint mobility in degrees<br>4 (2 to 6)<br>Favours passive movements		
	**GRADE**<br>Very low certainty<br>⊕◯◯◯	**Risk of bias**<br>Serious	**Inconsistency**<br>Serious	**Imprecision**<br>No serious	**Indirectness**<br>Serious	**Publication bias**<br>Serious

Study or Subgroup	Experimental Mean	Experimental SD	Experimental Total	Control Mean	Control SD	Control Total	Mean Difference IV, Fixed, 95% CI	Mean Difference IV, Fixed, 95% CI
Harvey et al, 2009	91	3.2269	20	87	3.2269	20	4.00 [2.00, 6.00]	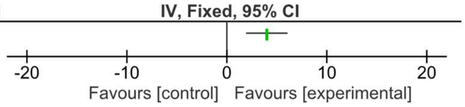

PASSIVE RANGE OF MOTION EXERCISES ON JOINT MOBILITY: GRADE Evidence to Decision						
PROBLEM	No	Probably no	Probably yes	**Yes**		Don't know
DESIRABLE EFFECTS	Trivial	**Small**	Moderate	Large		Don't know
UNDESIRABLE EFFECTS	Large	Moderate	Small	Trivial		**Don't know**
CERTAINTY OF EVIDENCE	**Very low**	Low	Moderate	High		No included studies
HOW MUCH PEOPLE VALUE THE MAIN OUTCOME	Important uncertainty or variability	Possibly important uncertainty or variability	**Probably no important uncertainty or variability**	No important uncertainty or variability		
BALANCE OF EFFECTS	Favours the Control	Probably favours the Control	Does not favour either the intervention (I) or the comparison (C)	**Probably favours the I**	Favours the I	Don't know
RESOURCES REQUIRED	Large costs	Moderate costs	**Negligible costs and savings**	Moderate savings	Large savings	Don't know
CERTAINTY OF EVIDENCE OF REQUIRED RESOURCES	Very low	Low	Moderate	High		**No included studies**

PASSIVE RANGE OF MOTION EXERCISES ON JOINT MOBILITY: GRADE Evidence to Decision						
COST EFFECTIVENESS	Favours the comparison	Probably favours the comparison	Does not favour either the intervention or the comparison	Probably favours the intervention	Favours the intervention	**No included studies**
EQUITY	Reduced	Probably reduced	**Probably no impact**	Probably increased	Increased	Don't know
ACCEPTABILITY	No	Probably no	Probably yes	**Yes**		Don't know
FEASIBILITY	No	Probably no	Probably yes	**Yes**		Don't know

PASSIVE RANGE OF MOTION EXERCISES ON JOINT MOBILITY: Randomised Controlled Trial Details

STUDY	COMPARISON	DOSAGE/DETAILS	PARTICIPANTS	N (RX/C)	OUTCOME	ROB 2 PEDRO
**HARVEY 2009**	Passive Movements V No intervention	10 minutes of ankle passive movements, 10 x per week for 6 months	C3 -C7 tetraplegia	20/20	Modified Ashworth	Some Concerns of Risk of Bias PEDro = 8/10

9. Physiotherapy interventions for spasticity

CONSENSUS-BASED OPINION STATEMENTS

Passive standing (v no intervention) on spasticity in people with SCI

**P**	People with SCI	**Evidence recommendation**<br>◯ No evidence recommendation<br>Reason: No recommendation due to insufficient or inconclusive evidence.	**Weak opinion statement <u>FOR</u>**<br>Passive standing may be provided to treat spasticity in people with SCI.<br><br>Clinical note: Passive standing includes standing in frames, devices or on a tilt table.
**I**	Passive standing		
**C**	No intervention	**Consensus-based opinion statement**<br>● Weak for (100%)	
**O**	Spasticity		
	**SUMMARY**	1 RCT[63]	Mean difference (95% CI): Spasticity on the Spinal Cord Injury Spasticity Evaluation Tool<br>0.1 (-0.3 to 0.1)<br>Favours no intervention

**GRADE**	**Risk of bias**	**Inconsistency**	**Imprecision**	**Indirectness**	**Publication bias**
Very low certainty ⊕◯◯◯	Serious	Serious	No serious	Serious	Serious

	Experimental			Control			Mean Difference
**Study or Subgroup**	**Mean**	**SD**	**Total**	**Mean**	**SD**	**Total**	**IV, Fixed, 95% CI**
Kwok et al, 2005	-0.1	0.3719	17	0	0.3719	17	-0.10 [-0.35, 0.15]

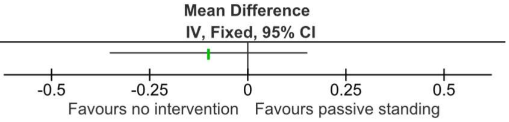

**PASSIVE STANDING ON SPASTICITY: GRADE Evidence to Decision**						
**PROBLEM**	No	Probably no	Probably yes	**Yes**		Don't know
**DESIRABLE EFFECTS**	Trivial	Small	Moderate	Large		**Don't know**
**UNDESIRABLE EFFECTS**	Large	Moderate	Small	Trivial		**Don't know**
**CERTAINTY OF EVIDENCE**	**Very low**	Low	Moderate	High		No included studies
**HOW MUCH PEOPLE VALUE THE MAIN OUTCOME**	Important uncertainty or variability	Possibly important uncertainty or variability	**Probably no important uncertainty or variability**	No important uncertainty or variability		

PASSIVE STANDING ON SPASTICITY: GRADE Evidence to Decision						
**BALANCE OF EFFECTS**	Favours the Control	**Probably favours the Control**	Does not favour either the intervention (I) or the comparison (C)	Probably favours the I	Favours the I	Don't know
**RESOURCES REQUIRED**	Large costs	**Moderate costs**	Negligible costs and savings	Moderate savings	Large savings	Don't know
**CERTAINTY OF EVIDENCE OF REQUIRED RESOURCES**	Very low	Low	Moderate	High		**No included studies**
**COST EFFECTIVENESS**	Favours the comparison	Probably favours the comparison	Does not favour either the intervention or the comparison	Probably favours the intervention	Favours the intervention	**No included studies**
**EQUITY**	Reduced	Probably reduced	**Probably no impact**	Probably increased	Increased	Don't know
**ACCEPTABILITY**	No	Probably no	Probably yes	**Yes**		Don't know
**FEASIBILITY**	No	Probably no	Probably yes	**Yes**		Don't know

PASSIVE STANDING ON SPASTICITY: Randomised Controlled Trial Details

STUDY	COMPARISON	DOSAGE/DETAILS	PARTICIPANTS	N (RX/C)	OUTCOME	ROB 2 PEDRO
**KWOK 2005**	Passive standing (and usual care) V Usual care	Tilt-table standing 5 x per week for 6 weeks (30 mins)	C5-T7 wheelchair dependent people with SCI	17/17	Spinal Cord Injury Spasticity Evaluation tool	Low Risk of Bias PEDro = 8/10

	FES cycling (v no intervention) on spasticity in people with SCI		
**P**	People with SCI	**Evidence recommendation**<br>◯ No evidence recommendation<br>Reason: No recommendation due to insufficient or inconclusive evidence.	**Weak opinion statement FOR**<br>FES cycling may be provided to treat spasticity in people with SCI.
**I**	FES cycling		
**C**	No intervention	**Consensus-based opinion statement**<br>● Weak for (100%)	
**O**	Spasticity		
	**SUMMARY**	1 RCT[64]	Mean difference (95% CI): Spasticity on the Ashworth Scale<br>-2 (-4 to 1)<br>Favours FES cycling

FES cycling (v no intervention) on spasticity in people with SCI

GRADE	Risk of bias	Inconsistency	Imprecision	Indirectness	Publication bias
Very low certainty ⨁◯◯◯	No serious	Serious	Serious	Serious	Serious

Study or Subgroup	Experimental Mean	Experimental SD	Experimental Total	Control Mean	Control SD	Control Total	Mean Difference IV, Fixed, 95% CI
Ralston et al, 2013	3.2	4.0497	14	5.1	4.0497	14	-1.90 [-4.90, 1.10]

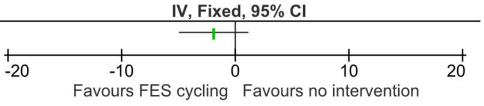

FES CYCLING ON SPASTICITY: GRADE Evidence to Decision						
PROBLEM	No	Probably no	Probably yes	**Yes**		Don't know
DESIRABLE EFFECTS	Trivial	Small	Moderate	Large		**Don't know**
UNDESIRABLE EFFECTS	Large	Moderate	Small	Trivial		**Don't know**
CERTAINTY OF EVIDENCE	**Very low**	Low	Moderate	High		No included studies
HOW MUCH PEOPLE VALUE THE MAIN OUTCOME	Important uncertainty or variability	Possibly important uncertainty or variability	**Probably no important uncertainty or variability**	No important uncertainty or variability		
BALANCE OF EFFECTS	Favours the Control	Probably favours the Control	Does not favour either the intervention (I) or the comparison (C)	**Probably favours the I**	Favours the I	Don't know
RESOURCES REQUIRED	Large costs	**Moderate costs**	Negligible costs and savings	Moderate savings	Large savings	Don't know
CERTAINTY OF EVIDENCE OF REQUIRED RESOURCES	Very low	Low	Moderate	High		**No included studies**
COST EFFECTIVENESS	Favours the comparison	Probably favours the comparison	Does not favour either the intervention or the comparison	Probably favours the intervention	Favours the intervention	**No included studies**
EQUITY	Reduced	**Probably reduced**	Probably no impact	Probably increased	Increased	Don't know
ACCEPTABILITY	No	Probably no	Probably yes	**Yes**		Don't know
FEASIBILITY	No	Probably no	**Probably yes**	Yes		Don't know

FES CYCLING ON SPASTICITY: Randomised Controlled Trial Details

STUDY	COMPARISON	DOSAGE/DETAILS	PARTICIPANTS	N (RX/C)	OUTCOME	ROB 2 PEDRO
RALSTON 2013	ES cycling V No intervention	Four x a week for two weeks (30-45 minutes)	C4 to T10 SCI	14/14	Spasticity - Ashworth	Low Risk of Bias PEDro = 8

	**Passive range of motion exercises (v no intervention) on spasticity in people with SCI**					
**P**	People with SCI	◯ No evidence recommendation<br>Reason: No recommendation due to insufficient or inconclusive evidence. Evidence recommendation		**Weak opinion statement AGAINST**<br>Passive range of motion exercises should not be provided to treat spasticity in people with SCI.		
**I**	Passive range of motion exercises					
**C**	No intervention	**Consensus-based opinion statement**<br>● Weak against (100%)				
**O**	Spasticity					
	**SUMMARY**	2 RCTs[61-62]		Mean difference (95% CI):<br>Consider studies independently. Unable to pool<br>$I^2 = 90\%$		
	**GRADE**<br>Very low certainty<br>⊕○○○	**Risk of bias**<br>Serious	**Inconsistency**<br>Very serious	**Imprecision**<br>Very serious	**Indirectness**<br>Very serious	**Publication bias**<br>Serious

Study or Subgroup	Experimental Mean	Experimental SD	Experimental Total	Control Mean	Control SD	Control Total	Mean Difference IV, Fixed, 95% CI
Chang et al, 2013	2.714286	1.112697	7	3.857143	0.899735	7	-1.14 [-2.20, -0.08]
Harvey et al, 2009	0	0.8067	20	0	0.8067	20	0.00 [-0.50, 0.50]

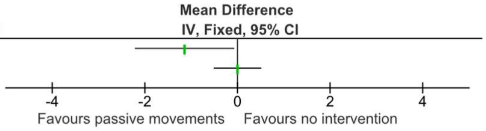

**PASSIVE RANGE OF MOTION EXERCISES ON SPASTICITY: GRADE Evidence to Decision**						
**PROBLEM**	No	Probably no	Probably yes	**Yes**		Don't know
**DESIRABLE EFFECTS**	Trivial	Small	Moderate	Large		**Don't know**
**UNDESIRABLE EFFECTS**	Large	Moderate	Small	Trivial		**Don't know**
**CERTAINTY OF EVIDENCE**	**Very low**	Low	Moderate	High		No included studies
**HOW MUCH PEOPLE VALUE THE MAIN OUTCOME**	Important uncertainty or variability	Possibly important uncertainty or variability	**Probably no important uncertainty or variability**	No important uncertainty or variability		
**BALANCE OF EFFECTS**	Favours the Control	Probably favours the Control	Does not favour either the intervention (I) or the comparison (C)	**Probably favours the I**	Favours the I	Don't know
**RESOURCES REQUIRED**	Large costs	Moderate costs	**Negligible costs and savings**	Moderate savings	Large savings	Don't know
**CERTAINTY OF EVIDENCE OF REQUIRED RESOURCES**	Very low	Low	Moderate	High		**No included studies**

PASSIVE RANGE OF MOTION EXERCISES ON SPASTICITY: GRADE Evidence to Decision						
COST EFFECTIVENESS	Favours the comparison	Probably favours the comparison	Does not favour either the intervention or the comparison	Probably favours the intervention	Favours the intervention	**No included studies**
EQUITY	Reduced	Probably reduced	**Probably no impact**	Probably increased	Increased	Don't know
ACCEPTABILITY	No	Probably no	Probably yes	**Yes**		Don't know
FEASIBILITY	No	Probably no	Probably yes	**Yes**		Don't know

PASSIVE RANGE OF MOTION EXERCISES ON SPASTICITY: Randomised Controlled Trial Details

STUDY	COMPARISON	DOSAGE/DETAILS	PARTICIPANTS	N (RX/C)	OUTCOME	ROB 2 PEDRO
**CHANG 2013**	Continuous Passive Motion (CPM) V No intervention	CPM of the ankle joint for 1 hour per day, 5 x per week for 4 weeks	C5-T12 SCI	7/7	Modified Ashworth	Some Concerns of Risk of Bias PEDro = 5/10
**HARVEY 2009**	Passive Movements V No intervention	10 minutes of ankle passive movements, 10 x per week for 6 months	C3 -C7 tetraplegia	20/20	Modified Ashworth	Some Concerns of Risk of Bias PEDro = 8/10

	Vibration (v no intervention) on spasticity in people with SCI		
**P**	People with SCI	**Evidence recommendation**<br>◯ No evidence recommendation<br>Reason: No RCTs	**No evidence recommendation or consensus-based opinion statement**
**I**	Vibration		
**C**	No intervention	**Consensus-based opinion statement**<br>◯ No consensus statements<br>Reason: No consensus could be reached	
**O**	Spasticity		

10. Physiotherapy interventions for bone mineral density

CONSENSUS-BASED OPINION STATEMENTS

	Passive standing (v no intervention) on bone mineral density					
**P**	People with SCI	**Evidence recommendation**<br>◯ No evidence recommendation<br>Reason: No recommendation due to insufficient or inconclusive evidence.		**No evidence recommendation or consensus-based opinion statement**<br><br>Clinical note: Passive standing includes standing in frames, devices or on a tilt table.		
**I**	Passive standing					
**C**	No intervention	**Consensus-based opinion statement**<br>◯ No consensus statements<br>Reason: No consensus could be reached				
**O**	Bone mineral density					
	**SUMMARY**	1 RCT[56]		Mean difference (95% CI): Bone mineral density g/cm^2<br>0.01 (-0.02 to 0.03)<br>Favours passive standing		
	**GRADE**<br>Very low certainty<br>⊕◯◯◯	**Risk of bias**<br>Serious	**Inconsistency**<br>Serious	**Imprecision**<br>No serious	**Indirectness**<br>Serious	**Publication bias**<br>Serious

Study or Subgroup	Experimental Mean	Experimental SD	Experimental Total	Control Mean	Control SD	Control Total	Mean Difference IV, Fixed, 95% CI
Ben et al 2005	0.857	0.0323	20	0.852	0.0323	20	0.01 [-0.02, 0.03]

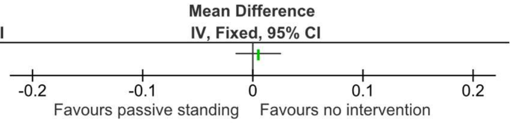

PASSIVE STANDING ON BONE MINERAL DENSITY: GRADE Evidence to Decision						
PROBLEM	No	Probably no	Probably yes	**Yes**		Don't know
DESIRABLE EFFECTS	Trivial	Small	Moderate	Large		**Don't know**
UNDESIRABLE EFFECTS	Large	Moderate	Small	Trivial		**Don't know**
CERTAINTY OF EVIDENCE	**Very low**	Low	Moderate	High		No included studies
HOW MUCH PEOPLE VALUE THE MAIN OUTCOME	Important uncertainty or variability	Possibly important uncertainty or variability	**Probably no important uncertainty or variability**	No important uncertainty or variability		

PASSIVE STANDING ON BONE MINERAL DENSITY: GRADE Evidence to Decision						
BALANCE OF EFFECTS	Favours the Control	Probably favours the Control	**Does not favour either the intervention (I) or the comparison (C)**	Probably favours the I	Favours the I	Don't know
RESOURCES REQUIRED	Large costs	**Moderate costs**	Negligible costs and savings	Moderate savings	Large savings	Don't know
CERTAINTY OF EVIDENCE OF REQUIRED RESOURCES	Very low	Low	Moderate	High		**No included studies**
COST EFFECTIVENESS	Favours the comparison	Probably favours the comparison	Does not favour either the intervention or the comparison	Probably favours the intervention	Favours the intervention	**No included studies**
EQUITY	Reduced	Probably reduced	**Probably no impact**	Probably increased	Increased	Don't know
ACCEPTABILITY	No	Probably no	Probably yes	**Yes**		Don't know
FEASIBILITY	No	Probably no	**Probably yes**	Yes		Don't know

PASSIVE STANDING ON BONE MINERAL DENSITY: Randomised Controlled Trial Details

STUDY	COMPARISON	DOSAGE/DETAILS	PARTICIPANTS	N (RX/C)	OUTCOME	ROB 2 PEDRO
**BEN 2005**	Long duration stretch V No intervention	30 minutes, 3 x per week for 12 weeks of standing on TT	People with SCI and LL paralysis	20/20	Bone mineral density g/cm2	Some Concerns of Risk of Bias PEDro = 8/10

11. Physiotherapy interventions for swelling

CONSENSUS-BASED OPINION STATEMENTS

	Elevation (v no intervention) on swelling in people with SCI		
**P**	People with SCI	**Evidence recommendation**<br>◯ No evidence recommendation<br>Reason: No RCTs	**Weak opinion statement <u>FOR</u>**<br>Elevation may be provided to treat extremity swelling in people with SCI.
**I**	Elevation		
**C**	No intervention	**Consensus-based opinion statement**<br>● Weak for (78%)	
**O**	Swelling		

	Neuromuscular electrical stimulation (v no intervention) on swelling in people with SCI		
**P**	People with SCI	**Evidence recommendation**<br>◯ No evidence recommendation<br>Reason: No RCTs	**Weak opinion statement <u>FOR</u>**<br>Neuromuscular electrical stimulation (NMES) may be provided to treat extremity swelling in people with SCI.<br><br>Clinical note: NMES for the treatment of swelling is only recommended for people who can be stimulated with NMES.
**I**	Neuromuscular electrical stimulation		
**C**	No intervention	**Consensus-based opinion statement**<br>● Weak for (96%)	
**O**	Swelling		

	Lymphatic massage (v no intervention) on swelling in people with SCI		
**P**	People with SCI	**Evidence recommendation**<br>◯ No evidence recommendation<br>Reason: No RCTs	**Weak opinion statement <u>FOR</u>**<br><br>Lymphatic massage may be provided to treat extremity swelling in people with SCI.
**I**	Lymphatic massage		
**C**	No intervention	**Consensus-based opinion statement**<br>● Weak for (93%)	
**O**	Swelling		

	FES cycling (v no intervention) on swelling in people with SCI					
**P**	People with SCI	**Evidence recommendation**<br>◯ No evidence recommendation<br>Reason: No recommendation due to insufficient or inconclusive evidence.		**Weak opinion statement <u>AGAINST</u>**<br><br>FES cycling should not be provided to decrease swelling in people with SCI.		
**I**	FES cycling					
**C**	No intervention	**Consensus-based opinion statement**<br>● Weak Against (86%)				
**O**	Swelling					
	**SUMMARY**	1 RCT[62]		Mean difference (95% CI): Swelling in cm<br>-0.1 (-1.5 to 1.3)<br>Favours no intervention		
	**GRADE**<br>Very low certainty<br>⊕◯◯◯	**Risk of bias**<br>No serious	**Inconsistency**<br>Serious	**Imprecision**<br>Serious	**Indirectness**<br>Serious	**Publication bias**<br>Serious

Study or Subgroup	Experimental Mean	Experimental SD	Experimental Total	Control Mean	Control SD	Control Total	Weight	Mean Difference IV, Fixed, 95% CI
Ralston et al, 2013	49.7	1.9	14	49.8	1.9	14		-0.10 [-1.51, 1.31]

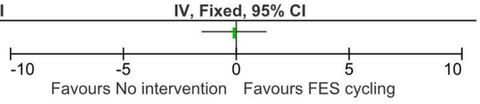

FES CYCLING ON SWELLING: GRADE Evidence to Decision						
**PROBLEM**	No	Probably no	Probably yes	**Yes**		Don't know
**DESIRABLE EFFECTS**	Trivial	Small	Moderate	Large		**Don't know**
**UNDESIRABLE EFFECTS**	Large	Moderate	Small	Trivial		**Don't know**
**CERTAINTY OF EVIDENCE**	**Very low**	Low	Moderate	High		No included studies
**HOW MUCH PEOPLE VALUE THE MAIN OUTCOME**	Important uncertainty or variability	Possibly important uncertainty or variability	**Probably no important uncertainty or variability**	No important uncertainty or variability		
**BALANCE OF EFFECTS**	Favours the Control	Probably favours the Control	**Does not favour either the intervention (I) or the comparison (C)**	Probably favours the I	Favours the I	Don't know
**RESOURCES REQUIRED**	**Large costs**	Moderate costs	Negligible costs and savings	Moderate savings	Large savings	Don't know
**CERTAINTY OF EVIDENCE OF REQUIRED RESOURCES**	Very low	Low	Moderate	High		**No included studies**
**COST EFFECTIVENESS**	Favours the comparison	Probably favours the comparison	Does not favour either the intervention or the comparison	Probably favours the intervention	Favours the intervention	**No included studies**
**EQUITY**	Reduced	**Probably reduced**	Probably no impact	Probably increased	Increased	Don't know
**ACCEPTABILITY**	No	Probably no	Probably yes	**Yes**		Don't know
**FEASIBILITY**	No	Probably no	Probably yes	**Yes**		Don't know

FES CYCLING ON SWELLING: Randomised Controlled Trial Details

STUDY	COMPARISON	DOSAGE/DETAILS	PARTICIPANTS	N (RX/C)	OUTCOME	ROB 2 PEDRO
**RALSTON 2013**	FES cycling V No intervention	Four x a week for two weeks (30-45 minutes)	C4 to T10 SCI	14/14	Swelling (cm)	Low Risk of Bias PEDro = 8

12. Physiotherapy interventions for strength

EVIDENCE RECOMMENDATIONS

	**Strength training (v no intervention) on voluntary strength of non-paralysed muscles in people with SCI**					
**P**	People with SCI (non-paralysed muscles)	**Evidence recommendation**<br>Weak for (91%)		**Weak evidence** recommendation **FOR**<br>Strength training may be provided to improve voluntary strength of non-paralysed muscles in people with SCI.		
**I**	Strength training					
**C**	No intervention	**Consensus-based opinion statement**<br>No opinion statements				
	Voluntary strength					
	**SUMMARY**	3 RCTs[53,65-66]		Consider studies independently. Unable to pool<br>$I^2 = 78\%$		
	**GRADE**<br>Very low certainty<br>⊕○○○	**Risk of bias**<br>Very serious	**Inconsistency**<br>Very serious	**Imprecision**<br>Serious	**Indirectness**<br>No serious	**Publication bias**<br>Serious

Study or Subgroup	Experimental Mean	Experimental SD	Experimental Total	Control Mean	Control SD	Control Total	Std. Mean Difference IV, Random, 95% CI
Hicks et al, 2003	18.1	7.3	11	13.2	6.2	12	0.70 [-0.15, 1.55]
Mulroy et al, 2011	74.2	28.3	26	63.3	19	32	0.46 [-0.07, 0.98]
Yildirim et al 2016	42.8	3.8	13	32.9	4.8	13	2.21 [1.21, 3.22]

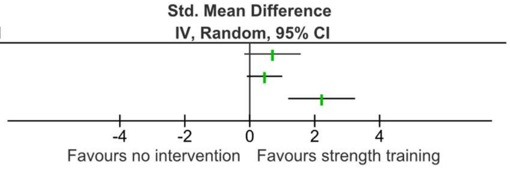

**STRENGTH TRAINING ON VOLUNTARY STRENGTH INNERVATED MUSCLES: GRADE Evidence to Decision**						
**PROBLEM**	No	Probably no	Probably yes	**Yes**		Don't know
**DESIRABLE EFFECTS**	Trivial	**Small**	Moderate	Large		Don't know
**UNDESIRABLE EFFECTS**	Large	Moderate	Small	Trivial		**Don't know**
**CERTAINTY OF EVIDENCE**	**Very low**	Low	Moderate	High		No included studies
**HOW MUCH PEOPLE VALUE THE MAIN OUTCOME**	Important uncertainty or variability	Possibly important uncertainty or variability	**Probably no important uncertainty or variability**	No important uncertainty or variability		

STRENGTH TRAINING ON VOLUNTARY STRENGTH INNERVATED MUSCLES: GRADE Evidence to Decision						
**BALANCE OF EFFECTS**	Favours the Control	Probably favours the Control	Does not favour either the intervention (I) or the comparison (C)	Probably favours the I	**Favours the I**	Don't know
**RESOURCES REQUIRED**	Large costs	Moderate costs	**Negligible costs and savings**	Moderate savings	Large savings	Don't know
**CERTAINTY OF EVIDENCE OF REQUIRED RESOURCES**	Very low	Low	Moderate	High		**No included studies**
**COST EFFECTIVENESS**	Favours the comparison	Probably favours the comparison	Does not favour either the intervention or the comparison	Probably favours the intervention	Favours the intervention	**No included studies**
**EQUITY**	Reduced	Probably reduced	**Probably no impact**	Probably increased	Increased	Don't know
**ACCEPTABILITY**	No	Probably no	Probably yes	**Yes**		Don't know
**FEASIBILITY**	No	Probably no	Probably yes	**Yes**		Don't know

STRENGTH TRAINING ON VOLUNTARY STRENGTH INNERVATED MUSCLES: Randomised Controlled Trial Details

STUDY	COMPARISON	DOSAGE/DETAILS	PARTICIPANTS	N (RX/C)	OUTCOME	ROB 2 PEDRO
**HICKS 2003**	Circuit training (Pushing, arm ergometry and PRE) V Education	Supervised progressive exercise 2 x weekly for 9 months. Each session 90-120 minutes	C4-L2 SCI	11/12	Elbow flexion strength in kg	High Risk of Bias PEDro = 5/10
**MULROY 2011**	Home-based shoulder exercise programme V Education	3 x per week for 12 weeks	T2 to T7 SCI	26/32	Shoulder abduction in Nm	High Risk of Bias PEDro = 7/10
**YILDRIM 2016**	Strength training V No intervention	Upper extremity training 5 x per week for 6 weeks	Paraplegia	13/13	Elbow flexion Nm/kg	Some Concerns of Risk of Bias PEDro = 4/10

Strength training (v no intervention) on voluntary strength of partially paralysed muscles in people with SCI

**P**	People with SCI (partially-paralysed muscles)	**Evidence recommendation**<br>◯ Weak for (90%)	**Weak evidence recommendation FOR**<br>Strength training may be provided to improve voluntary strength of partially paralysed muscles in people with SCI.
**I**	Strength training		
**C**	No intervention	**Consensus-based opinion statement**<br>◯ No opinion statements	
**O**	Voluntary strength		
	**SUMMARY**	3 RCTs[67-69]	Mean difference (95% CI):<br>0.4 (0 to 0.9)<br>Favours strength training

GRADE	Risk of bias	Inconsistency	Imprecision	Indirectness	Publication bias
Very low certainty ⊕◯◯◯	Serious	Very serious	Serious	Serious	Serious

Study or Subgroup	Experimental Mean	Experimental SD	Experimental Total	Control Mean	Control SD	Control Total	Weight	Std. Mean Difference IV, Random, 95% CI
Bye et al 2017	29.1	4.7425	30	24.8	4.7425	30	32.9%	0.89 [0.36, 1.43]
Chen et al, 2020	6.9	2.4834	58	6.5	2.4834	59	41.7%	0.16 [-0.20, 0.52]
Glinsky et al, 2008	3.3	0.9937	15	3.1	0.9937	16	25.3%	0.20 [-0.51, 0.90]
**Total (95% CI)**			**103**			**105**	**100.0%**	**0.41 [-0.08, 0.90]**

Heterogeneity: Tau² = 0.11; Chi² = 5.25, df = 2 (P = 0.07); I² = 62%
Test for overall effect: Z = 1.66 (P = 0.10)

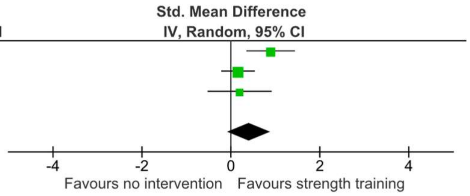

STRENGTH TRAINING ON VOLUNTARY STRENGTH PARTIALLY PARALYSED MUSCLES: GRADE Evidence to Decision						
**PROBLEM**	No	Probably no	Probably yes	**Yes**		Don't know
**DESIRABLE EFFECTS**	Trivial	Small	**Moderate**	Large		Don't know
**UNDESIRABLE EFFECTS**	Large	Moderate	Small	Trivial		**Don't know**
**CERTAINTY OF EVIDENCE**	**Very low**	Low	Moderate	High		No included studies
**HOW MUCH PEOPLE VALUE THE MAIN OUTCOME**	Important uncertainty or variability	Possibly important uncertainty or variability	**Probably no important uncertainty or variability**	No important uncertainty or variability		
**BALANCE OF EFFECTS**	Favours the Control	Probably favours the Control	Does not favour either the intervention (I) or the comparison (C)	Probably favours the I	**Favours the I**	Don't know
**RESOURCES REQUIRED**	Large costs	Moderate costs	**Negligible costs and savings**	Moderate savings	Large savings	Don't know

STRENGTH TRAINING ON VOLUNTARY STRENGTH PARTIALLY PARALYSED MUSCLES: GRADE Evidence to Decision						
CERTAINTY OF EVIDENCE OF REQUIRED RESOURCES	Very low	Low	Moderate	High		**No included studies**
COST EFFECTIVENESS	Favours the comparison	Probably favours the comparison	Does not favour either the intervention or the comparison	Probably favours the intervention	Favours the intervention	**No included studies**
EQUITY	Reduced	Probably reduced	**Probably no impact**	Probably increased	Increased	Don't know
ACCEPTABILITY	No	Probably no	Probably yes	**Yes**		Don't know
FEASIBILITY	No	Probably no	Probably yes	**Yes**		Don't know

STRENGTH TRAINING ON VOLUNTARY STRENGTH PARTIALLY PARALYSED MUSCLES: Randomised Controlled Trial Details

STUDY	COMPARISON	DOSAGE/DETAILS	PARTICIPANTS	N (RX/C)	OUTCOME	ROB 2 PEDRO
**BYE 2017**	Strength training V No intervention	4 sets of 10RM, 3 x per week for 12 weeks	C1-S5 SCI	30/30	Maximal voluntary isometric strength in Nm	Some Concerns of Risk of Bias PEDro = 8/10
**CHEN 2020**	Strength training V No intervention	200 contraction per day, 6 days per week for 6 weeks	C1-S5 SCI	58/59	Strength manual muscle test	Some Concerns of Risk of Bias PEDro = 8/10
**GLINSKY 2008**	Strength training V No intervention	3 sets of 10RM, 3 x per week for 8 weeks	C4-C7 tetraplegia	15/16	Strength in Nm	Some Concerns of Risk of Bias PEDro = 8/10

FES cycling (v no intervention) on atrophy (prevention) in people with SCI and paralysis of the lower limbs

**P**	People with SCI who have paralysis of the lower limbs	**Evidence recommendation**<br>Weak for (100%)	**Weak evidence recommendation FOR**<br>FES cycling may be provided to decrease atrophy in people with SCI and paralysis of the lower limbs
**I**	FES cycling		
**C**	No intervention	**Consensus-based opinion statement**<br>No opinion statement	
**O**	Atrophy		
	**SUMMARY**	2 RCTs[70-71]	Standardised Mean Difference (95% CI)<br>3 (2 to 4)<br>Favours FES cycling

**GRADE**	**Risk of bias**	**Inconsistency**	**Imprecision**	**Indirectness**	**Publication bias**
Very low certainty ⊕○○○	Very serious	No serious	Serious	Serious	Serious

Study or Subgroup	Experimental Mean	Experimental SD	Experimental Total	Control Mean	Control SD	Control Total	Weight	Std. Mean Difference IV, Fixed, 95% CI
Baldi et al, 1998	7.7	8.7	9	-26.8	13.2	9	69.0%	2.94 [1.51, 4.37]
Demchak et al, 2005	5,500	1,192	5	1,566	1,118	5	31.0%	3.07 [0.95, 5.20]
**Total (95% CI)**			**14**			**14**	**100.0%**	**2.98 [1.80, 4.17]**

Heterogeneity: Chi² = 0.01, df = 1 (P = 0.92); I² = 0%
Test for overall effect: Z = 4.93 (P < 0.00001)

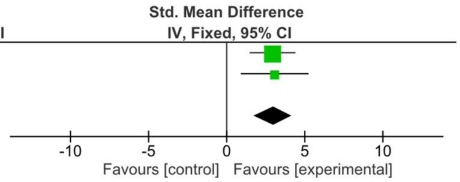

**FES CYCLING ON ATROPHY: GRADE Evidence to Decision**						
**PROBLEM**	No	Probably no	Probably yes	**Yes**		Don't know
**DESIRABLE EFFECTS**	Trivial	Small	Moderate	**Large**		Don't know
**UNDESIRABLE EFFECTS**	Large	Moderate	Small	Trivial		**Don't know**
**CERTAINTY OF EVIDENCE**	**Very low**	Low	Moderate	High		No included studies
**HOW MUCH PEOPLE VALUE THE MAIN OUTCOME**	Important uncertainty or variability	Possibly important uncertainty or variability	**Probably no important uncertainty or variability**	No important uncertainty or variability		
**BALANCE OF EFFECTS**	Favours the Control	Probably favours the Control	Does not favour either the intervention (I) or the comparison (C)	Probably favours the I	**Favours the I**	Don't know
**RESOURCES REQUIRED**	Large costs	**Moderate costs**	Negligible costs and savings	Moderate savings	Large savings	Don't know

FES CYCLING ON ATROPHY: GRADE Evidence to Decision						
CERTAINTY OF EVIDENCE OF REQUIRED RESOURCES	Very low	Low	Moderate	High		**No included studies**
COST EFFECTIVENESS	Favours the comparison	Probably favours the comparison	Does not favour either the intervention or the comparison	Probably favours the intervention	Favours the intervention	**No included studies**
EQUITY	Reduced	**Probably reduced**	Probably no impact	Probably increased	Increased	Don't know
ACCEPTABILITY	No	Probably no	Probably yes	**Yes**		Don't know
FEASIBILITY	No	Probably no	**Probably yes**	Yes		Don't know

FES CYCLING ON ATROPHY: Randomised Controlled Trial Details

STUDY	COMPARISON	DOSAGE/DETAILS	PARTICIPANTS	N (RX/C)	OUTCOME	ROB 2 PEDRO
**BALDI 1998**	FES cycle ergometry V No intervention	FES cycle ergometer 3 x per week for 3 weeks	C5-T12 Frankel A and B SCI	9/9	Atrophy Total body mass (gluteal)	High Risk of Bias PEDro = 4/10
**DEMCHAK 2005**	FES cycle ergometry V No intervention	FES cycle ergometer 3 x per week for 13 weeks	AIS A and B SCI	5/5	Atrophy – Muscle fibre cross sectional area	High Risk of Bias PEDro = 5/10

	Electrical stimulation alone (v no intervention) on voluntary strength of partially paralysed muscles in people with SCI					
**P**	People with SCI (partially-paralysed muscles)	Evidence recommendation<br>Weak Against (96%)		**Weak evidence recommendation AGAINST**<br>Electrical stimulation alone should not be provided to improve voluntary strength of partially paralysed muscles in people with SCI.<br>Clinical note: When electrical stimulation is used in partially paralysed muscles it should be combined with voluntary effort.		
**I**	Electrical stimulation alone					
**C**	No intervention	**Consensus-based opinion statement**<br>No opinion statements				
**O**	Voluntary strength					
	**SUMMARY**	1 RCT[72]		Mean difference (95% CI): Strength in Nm<br>0 (-0.5 to 0.6)<br>Favours electrical stimulation		
	**GRADE**<br>Very Low certainty<br>⊕○○○	**Risk of bias**<br>Serious	**Inconsistency**<br>Serious	**Imprecision**<br>No serious	**Indirectness**<br>Serious	**Publication bias**<br>Serious

Study or Subgroup	Experimental Mean	Experimental SD	Experimental Total	Control Mean	Control SD	Control Total	Mean Difference IV, Fixed, 95% CI
Glinsky et al, 2009	3.34	1.1021	32	3.3	1.1021	32	0.04 [-0.50, 0.58]

Mean Difference
IV, Fixed, 95% CI

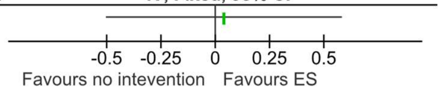

ELECTRICAL STIMULATION ON VOLUNTARY STRENGTH PARTIALLY PARALYSED MUSCLES: GRADE Evidence to Decision						
**PROBLEM**	No	Probably no	Probably yes	**Yes**		Don't know
**DESIRABLE EFFECTS**	Trivial	Small	Moderate	Large		**Don't know**
**UNDESIRABLE EFFECTS**	Large	Moderate	Small	Trivial		**Don't know**
**CERTAINTY OF EVIDENCE**	**Very low**	Low	Moderate	High		No included studies
**HOW MUCH PEOPLE VALUE THE MAIN OUTCOME**	Important uncertainty or variability	Possibly important uncertainty or variability	**Probably no important uncertainty or variability**	No important uncertainty or variability		
**BALANCE OF EFFECTS**	Favours the Control	Probably favours the Control	**Does not favour either the intervention (I) or the comparison (C)**	Probably favours the I	Favours the I	Don't know
**RESOURCES REQUIRED**	Large costs	Moderate costs	**Negligible costs and savings**	Moderate savings	Large savings	Don't know
**CERTAINTY OF EVIDENCE OF REQUIRED RESOURCES**	Very low	Low	Moderate	High		**No included studies**

ELECTRICAL STIMULATION ON VOLUNTARY STRENGTH PARTIALLY PARALYSED MUSCLES: GRADE Evidence to Decision						
**COST EFFECTIVENESS**	Favours the comparison	Probably favours the comparison	Does not favour either the intervention or the comparison	Probably favours the intervention	Favours the intervention	**No included studies**
**EQUITY**	Reduced	Probably reduced	**Probably no impact**	Probably increased	Increased	Don't know
**ACCEPTABILITY**	No	Probably no	Probably yes	**Yes**		Don't know
**FEASIBILITY**	No	Probably no	Probably yes	**Yes**		Don't know

ELECTRICAL STIMULATION ON VOLUNTARY STRENGTH PARTIALLY PARALYSED MUSCLES: Randomised Controlled Trial Details

STUDY	COMPARISON	DOSAGE/DETAILS	PARTICIPANTS	N (RX/C)	OUTCOME	ROB 2 PEDRO
**GLINSKY 2009**	Strength training plus ES V Strength training and Sham ES	6 sets of 10 Reps, 3 x per week for 8 weeks	C4 to C7 tetraplegia	32/32	Strength in Nm	Some Concerns of Risk of Bias PEDro = 9/10

CONSENSUS-BASED OPINION STATEMENTS

	Strength training combined with electrical stimulation (v no intervention) on voluntary strength of partially paralysed muscles in people with SCI.					
**P**	People with SCI (partially-paralysed muscles)	**Evidence recommendation**<br>○ No evidence recommendation<br>Reason: No recommendation due to insufficient or inconclusive evidence.		**Weak opinion statement <u>FOR</u>**<br>Electrical Stimulation combined with strength training may be provided to improve voluntary strength of partially paralysed muscles in people with SCI.		
**I**	Electrical Stimulation combined with strength training					
**C**	No intervention	**Consensus-based opinion statement**<br>● Weak for (95%)				
**O**	Voluntary strength					
	**SUMMARY**	1 RCT[73]		Mean difference (95% CI): Strength in Nm<br>14 (1 to 27)<br>Favours electrical stimulation combined with strength training		
	**GRADE**<br>Very low certainty<br>⊕○○○	**Risk of bias**<br>Serious	**Inconsistency**<br>Serious	**Imprecision**<br>Serious	**Indirectness**<br>Serious	**Publication bias**<br>Serious

Study or Subgroup	Experimental Mean	SD	Total	Control Mean	SD	Total	Mean Difference IV, Fixed, 95% CI
Harvey et al, 2010	66	14.8313	10	52	14.8313	10	14.00 [1.00, 27.00]

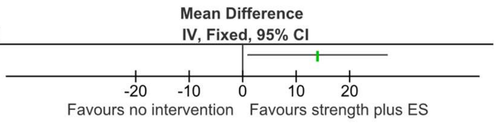

STRENGTH TRAINING PLUS ELECTRICAL STIMULATION ON VOLUNTARY STRENGTH PARTIALLY PARALYSED MUSCLES: GRADE Evidence to Decision						
PROBLEM	No	Probably no	Probably yes	**Yes**		Don't know
DESIRABLE EFFECTS	Trivial	**Small**	Moderate	Large		Don't know
UNDESIRABLE EFFECTS	Large	Moderate	Small	Trivial		**Don't know**
CERTAINTY OF EVIDENCE	**Very low**	Low	Moderate	High		No included studies
HOW MUCH PEOPLE VALUE THE MAIN OUTCOME	Important uncertainty or variability	Possibly important uncertainty or variability	**Probably no important uncertainty or variability**	No important uncertainty or variability		
BALANCE OF EFFECTS	Favours the Control	Probably favours the Control	Does not favour either the intervention (I) or the comparison (C)	Probably favours the I	**Favours the I**	Don't know
RESOURCES REQUIRED	Large costs	Moderate costs	**Negligible costs and savings**	Moderate savings	Large savings	Don't know

**STRENGTH TRAINING PLUS ELECTRICAL STIMULATION ON VOLUNTARY STRENGTH PARTIALLY PARALYSED MUSCLES: GRADE Evidence to Decision**						
**CERTAINTY OF EVIDENCE OF REQUIRED RESOURCES**	Very low	Low	Moderate	High		**No included studies**
**COST EFFECTIVENESS**	Favours the comparison	Probably favours the comparison	Does not favour either the intervention or the comparison	Probably favours the intervention	Favours the intervention	**No included studies**
**EQUITY**	Reduced	Probably reduced	**Probably no impact**	Probably increased	Increased	Don't know
**ACCEPTABILITY**	No	Probably no	Probably yes	**Yes**		Don't know
**FEASIBILITY**	No	Probably no	Probably yes	**Yes**		Don't know

STRENGTH TRAINING PLUS ELECTRICAL STIMULATION ON VOLUNTARY STRENGTH PARTIALLY PARALYSED MUSCLES: Randomised Controlled Trial Details

STUDY	COMPARISON	DOSAGE/DETAILS	PARTICIPANTS	N (RX/C)	OUTCOME	ROB 2 PEDRO
**HARVEY 2010**	Strength training plus electrical stimulation V No intervention	12 sets of 10 3 x per week for 8 weeks	C3-L2 SCI	10/10	Strength in Nm	Some Concerns of Risk of Bias PEDro = 8/10

Whole body vibration (v no intervention) on voluntary strength in people with SCI

**P**	People with SCI	**Evidence recommendation**<br>○ No evidence recommendation<br>Reason: No recommendation due to insufficient or inconclusive evidence.	**Strong opinion statement AGAINST**<br>Whole body vibration should not be provided to improve voluntary strength in people with SCI.
**I**	Whole body vibration		
**C**	No intervention	**Consensus-based opinion statement**<br>● Strong Against (77%)	
**O**	Strength		
	**SUMMARY**	1 RCT[74]	Mean difference (95% CI): Strength in kg<br>1.1 (-8.0 to 10.27)<br>Favours vibration

**GRADE**	**Risk of bias**	**Inconsistency**	**Imprecision**	**Indirectness**	**Publication bias**
Very low certainty<br>⊕○○○	Serious	Serious	Very serious	Serious	Serious

Study or Subgroup	Experimental Mean	Experimental SD	Experimental Total	Control Mean	Control SD	Control Total	Mean Difference IV, Fixed, 95% CI
Bosveld et al, 2015	1.41	11.4315	12	0.29	11.4315	12	1.12 [-8.03, 10.27]

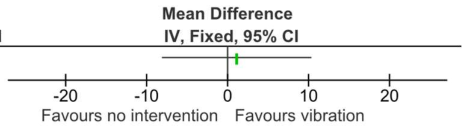

VIBRATION ON VOLUNTARY STRENGTH PARTIALLY PARALYSED MUSCLES: GRADE Evidence to Decision						
**PROBLEM**	No	Probably no	Probably yes	**Yes**		Don't know
**DESIRABLE EFFECTS**	Trivial	Small	Moderate	Large		**Don't know**
**UNDESIRABLE EFFECTS**	Large	Moderate	Small	Trivial		**Don't know**
**CERTAINTY OF EVIDENCE**	**Very low**	Low	Moderate	High		No included studies
**HOW MUCH PEOPLE VALUE THE MAIN OUTCOME**	Important uncertainty or variability	Possibly important uncertainty or variability	**Probably no important uncertainty or variability**	No important uncertainty or variability		
**BALANCE OF EFFECTS**	Favours the Control	Probably favours the Control	**Does not favour either the intervention (I) or the comparison (C)**	Probably favours the I	Favours the I	Don't know
**RESOURCES REQUIRED**	Large costs	**Moderate costs**	Negligible costs and savings	Moderate savings	Large savings	Don't know
**CERTAINTY OF EVIDENCE OF REQUIRED RESOURCES**	Very low	Low	Moderate	High		**No included studies**

VIBRATION ON VOLUNTARY STRENGTH PARTIALLY PARALYSED MUSCLES: GRADE Evidence to Decision						
COST EFFECTIVENESS	Favours the comparison	Probably favours the comparison	Does not favour either the intervention or the comparison	Probably favours the intervention	Favours the intervention	**No included studies**
EQUITY	Reduced	Probably reduced	**Probably no impact**	Probably increased	Increased	Don't know
ACCEPTABILITY	No	Probably no	Probably yes	**Yes**		Don't know
FEASIBILITY	No	**Probably no**	Probably yes	Yes		Don't know

VIBRATION ON VOLUNTARY STRENGTH PARTIALLY PARALYSED MUSCLES: Randomised Controlled Trial Details

STUDY	COMPARISON	DOSAGE/DETAILS	PARTICIPANTS	N (RX/C)	OUTCOME	ROB 2 PEDRO
**BOSVELD 2015**	Whole body vibration on platform V Sham vibration on platform	Whole body Vibration (four 45-second bouts with 1-minute intervening rest periods)	Chronic motor incomplete SCI C2 to T12F	12/12	Maximal isometric quadriceps strength in kg	Some Concerns of Risk of Bias PEDro = 4/10

13. Physiotherapy interventions for fitness and cardiorespiratory health

EVIDENCE RECOMMENDATIONS

	Arm cranking (v no intervention) on cardiorespiratory fitness in people with SCI				
**P**	People with SCI	**Evidence recommendation**<br>◯ Weak for (81%)		**Weak evidence recommendation <u>FOR</u>**<br>Arm cranking may be provided to improve cardiorespiratory fitness in people with SCI.<br>Clinical note: Arm cranking for cardiorespiratory fitness may not be appropriate for people with shoulder pain or overuse.	
**I**	Arm cranking				
**C**	No intervention	**Consensus-based opinion statement**<br>◯ No opinion statements			
**O**	Cardiorespiratory Fitness				
	**SUMMARY**	3 RCTs [54,75-76]		Mean difference (95% CI): Cardiorespiratory fitness expressed as Vo2 Peak<br>4.7 (1.4 to 8.0)<br>Favours arm cranking	
	**GRADE**<br>Very low certainty<br>⊕○○○	**Risk of bias**<br>Very serious	**Inconsistency**<br>No serious	**Imprecision**<br>No serious	**Indirectness**<br>Serious
				**Publication bias**<br>Serious	

Study or Subgroup	Experimental Mean	Experimental SD	Experimental Total	Control Mean	Control SD	Control Total	Weight	Mean Difference IV, Fixed, 95% CI
Akkurt et al, 2017	23.4	7.4869	17	16.8	7.4869	16	40.9%	6.60 [1.49, 11.71]
Nightingale et al, 2018	21.7	5.1	13	18.3	6.3	8	40.0%	3.40 [-1.77, 8.57]
Taylor et al, 1986	26.3	3	5	23	8	5	19.1%	3.30 [-4.19, 10.79]
**Total (95% CI)**			**35**			**29**	**100.0%**	**4.69 [1.42, 7.96]**

Heterogeneity: Chi² = 0.91, df = 2 (P = 0.64); I² = 0%
Test for overall effect: Z = 2.81 (P = 0.005)

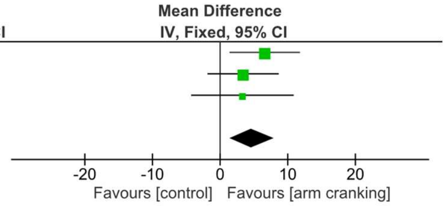

ARM CRANKING ON CARDIORESPIRATORY FITNESS: GRADE Evidence to Decision						
**PROBLEM**	No	Probably no	Probably yes	**Yes**		Don't know
**DESIRABLE EFFECTS**	Trivial	Small	**Moderate**	Large		Don't know
**UNDESIRABLE EFFECTS**	Large	Moderate	Small	Trivial		**Don't know**
**CERTAINTY OF EVIDENCE**	**Very low**	Low	Moderate	High		No included studies
**HOW MUCH PEOPLE VALUE THE MAIN OUTCOME**	Important uncertainty or variability	Possibly important uncertainty or variability	**Probably no important uncertainty or variability**	No important uncertainty or variability		

ARM CRANKING ON CARDIORESPIRATORY FITNESS: GRADE Evidence to Decision						
BALANCE OF EFFECTS	Favours the Control	Probably favours the Control	Does not favour either the intervention (I) or the comparison (C)	Probably favours the I	**Favours the I**	Don't know
RESOURCES REQUIRED	Large costs	**Moderate costs**	Negligible costs and savings	Moderate savings	Large savings	Don't know
CERTAINTY OF EVIDENCE OF REQUIRED RESOURCES	Very low	Low	Moderate	High		**No included studies**
COST EFFECTIVENESS	Favours the comparison	Probably favours the comparison	Does not favour either the intervention or the comparison	Probably favours the intervention	Favours the intervention	**No included studies**
EQUITY	Reduced	**Probably reduced**	Probably no impact	Probably increased	Increased	Don't know
ACCEPTABILITY	No	Probably no	Probably yes	**Yes**		Don't know
FEASIBILITY	No	Probably no	Probably yes	**Yes**		Don't know

ARM CRANKING ON CARDIORESPIRATORY FITNESS: Randomised Controlled Trial Details

STUDY	COMPARISON	DOSAGE/DETAILS	PARTICIPANTS	N (RX/C)	OUTCOME	ROB 2 PEDRO
**AKKURT 2017**	Arm cranking (plus usual care) V Usual care	3 days per week, 1.5 hours/week 50-70% pVO2 (A borg scale score of lightly hard-moderately hard)	C7-L5 SCI	17/16	Vo2 peak	High Risk of Bias PEDro = 6/10
**NIGHTINGALE 2018**	Arm cranking (portable desktop ergometer) V No intervention	4 x per week for 6 weeks (moderate intensity)	Below T2 SCI	13/8	Vo2 peak	Some Concerns of Risk of Bias PEDro = 5/10
**TAYLOR 1986**	Arm cranking V No intervention	30 minutes, 5 x per week for 8 consecutive weeks (50rev/min)	paraplegia	5/5	Vo2 peak	High Risk of Bias PEDro = 5/10

Hand Cycling (v no intervention) on cardiorespiratory fitness in people with SCI

**P**	People with SCI	**Evidence recommendation**<br>◯ Weak for (88%)	**Weak evidence recommendation FOR**<br>Hand cycling may be provided to improve cardiorespiratory fitness in people with SCI.<br><br>Clinical note: Hand cycling for cardiorespiratory fitness may not be appropriate for people with shoulder pain or overuse
**I**	Hand cycling		
**C**	No intervention	**Consensus-based opinion statement**<br>◯ No opinion statements	
**O**	Cardiorespiratory Fitness		
	**SUMMARY**	1 RCT[77]	Mean difference (95% CI): Cardiorespiratory fitness expressed as Vo2 Peak<br>5.9 (3.7 to 8.1)<br>Favours hand cycling

GRADE	Risk of bias	Inconsistency	Imprecision	Indirectness	Publication bias
Very low certainty ⊕○○○	Serious	Serious	No serious	Serious	Serious

Study or Subgroup	Experimental Mean	Experimental SD	Experimental Total	Control Mean	Control SD	Control Total	Mean Difference IV, Fixed, 95% CI
Kim et al, 2015	4.4	3.1	8	-1.5	0.7	7	5.90 [3.69, 8.11]

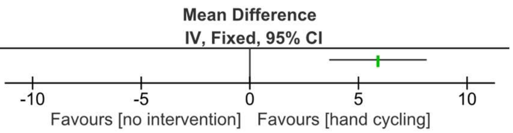

HAND CYCLING ON CARDIORESPIRATORY FITNESS: GRADE Evidence to Decision						
**PROBLEM**	No	Probably no	Probably yes	**Yes**		Don't know
**DESIRABLE EFFECTS**	Trivial	**Small**	Moderate	Large		Don't know
**UNDESIRABLE EFFECTS**	Large	Moderate	Small	Trivial		**Don't know**
**CERTAINTY OF EVDENCE**	**Very low**	Low	Moderate	High		No included studies
**HOW MUCH PEOPLE VALUE THE MAIN OUTCOME**	Important uncertainty or variability	Possibly important uncertainty or variability	**Probably no important uncertainty or variability**	No important uncertainty or variability		
**BALANCE OF EFFECTS**	Favours the Control	Probably favours the Control	Does not favour either the intervention (I) or the comparison (C)	Probably favours the I	**Favours the I**	Don't know
**RESOURCES REQUIRED**	Large costs	**Moderate costs**	Negligible costs and savings	Moderate savings	Large savings	Don't know
**CERTAINTY OF EVIDENCE OF REQUIRED RESOURCES**	Very low	Low	Moderate	High		**No included studies**

HAND CYCLING ON CARDIORESPIRATORY FITNESS: GRADE Evidence to Decision						
COST EFFECTIVENESS	Favours the comparison	Probably favours the comparison	Does not favour either the intervention or the comparison	Probably favours the intervention	Favours the intervention	**No included studies**
EQUITY	Reduced	**Probably reduced**	Probably no impact	Probably increased	Increased	Don't know
ACCEPTABILITY	No	Probably no	Probably yes	**Yes**		Don't know
FEASIBILITY	No	Probably no	**Probably yes**	Yes		Don't know

HAND CYCLING ON CARDIORESPIRATORY FITNESS: Randomised Controlled Trial Details

STUDY	COMPARISON	DOSAGE/DETAILS	PARTICIPANTS	N (RX/C)	OUTCOME	ROB 2 PEDRO
**KIM 2015**	Indoor hand cycling V No intervention (usual activities)	Indoor hand bike - 60 minutes per day, 3 days per week for 6 weeks	C5-T11 SCI	8/7	Vo2 peak	Some Concerns of Risk of Bias PEDro = 5/10

Circuit training (v no intervention) on cardiorespiratory fitness in people with SCI

**P**	People with SCI	**Evidence recommendation**<br>◯ Weak for (100%)	**Weak evidence recommendation FOR**<br>Circuit training may be provided to improve cardiorespiratory fitness in people with SCI.
**I**	Circuit training		
**C**	No intervention	**Consensus-based opinion statement**<br>◯ No opinion statements	
**O**	Cardiorespiratory Fitness		
	**SUMMARY**	4 RCTs[65,78-80]	Standardised Mean Difference (95% CI)<br>0.5 (0 to 0.9)<br>Favours circuit training

**GRADE**<br>Very low certainty<br>⊕◯◯◯	**Risk of bias**<br>Serious	**Inconsistency**<br>No serious	**Imprecision**<br>Very serious	**Indirectness**<br>Serious	**Publication bias**<br>Serious

Study or Subgroup	Experimental Mean	Experimental SD	Experimental Total	Control Mean	Control SD	Control Total	Weight	Std. Mean Difference IV, Fixed, 95% CI
Bombardier et al, 2000	0	17.4909	6	-1	17.4909	7	16.9%	0.05 [-1.04, 1.14]
Hicks et al, 2003	45.9	32.4	11	32.5	20.4	12	29.1%	0.48 [-0.35, 1.31]
Kim et al, 2019	3.5	2.4	11	2.3	3.5	6	19.9%	0.40 [-0.60, 1.41]
Ma et al, 2019	17.83	4.91	14	13.94	5.51	14	34.1%	0.72 [-0.04, 1.49]
**Total (95% CI)**			**42**			**39**	**100.0%**	**0.48 [0.03, 0.93]**

Heterogeneity: Chi² = 1.00, df = 3 (P = 0.80); I² = 0%
Test for overall effect: Z = 2.08 (P = 0.04)

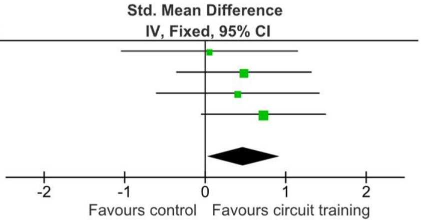

CIRCUIT TRAINING ON CARDIORESPIRATORY FITNESS: GRADE Evidence to Decision						
PROBLEM	No	Probably no	Probably yes	**Yes**		Don't know
DESIRABLE EFFECTS	Trivial	**Small**	Moderate	Large		Don't know
UNDESIRABLE EFFECTS	Large	Moderate	Small	Trivial		**Don't know**
CERTAINTY OF EVIDENCE	**Very low**	Low	Moderate	High		No included studies
HOW MUCH PEOPLE VALUE THE MAIN OUTCOME	Important uncertainty or variability	Possibly important uncertainty or variability	**Probably no important uncertainty or variability**	No important uncertainty or variability		
BALANCE OF EFFECTS	Favours the Control	Probably favours the Control	Does not favour either the intervention (I) or the comparison (C)	Probably favours the I	**Favours the I**	Don't know
RESOURCES REQUIRED	Large costs	**Moderate costs**	Negligible costs and savings	Moderate savings	Large savings	Don't know

CIRCUIT TRAINING ON CARDIORESPIRATORY FITNESS: GRADE Evidence to Decision						
CERTAINTY OF EVIDENCE OF REQUIRED RESOURCES	Very low	Low	Moderate	High		**No included studies**
COST EFFECTIVENESS	Favours the comparison	Probably favours the comparison	Does not favour either the intervention or the comparison	Probably favours the intervention	Favours the intervention	**No included studies**
EQUITY	Reduced	**Probably reduced**	Probably no impact	Probably increased	Increased	Don't know
ACCEPTABILITY	No	Probably no	Probably yes	**Yes**		Don't know
FEASIBILITY	No	Probably no	**Probably yes**	Yes		Don't know

CIRCUIT TRAINING ON CARDIORESPIRATORY FITNESS: Randomised Controlled Trial Details

STUDY	COMPARISON	DOSAGE/DETAILS	PARTICIPANTS	N (RX/C)	OUTCOME	ROB 2 PEDRO
**BOMBARDIER 2000**	Circuit training (telehealth) V No intervention	16 sessions of telehealth over 6 months	People with SCI	6/7	Vo2 Peak	Some Concerns of Risk of Bias PEDro = 6/10
**HICKS 2003**	Circuit training (Pushing, arm ergometry and PRE) V Sham (education)	Supervised progressive exercise 2 x weekly for 9 months. Each session 90-120 minutes	C4-L2 SCI	11/10	Power output in Watts	High Risk of Bias PEDro = 5/10
**KIM 2019**	Circuit training (Resistance and aerobic training) V No intervention	3 x weekly for 6 weeks. Each session was one hour	C5-T10 SCI	11/6	Vo2 Peak	Some Concerns of Risk of Bias PEDro = 6/10
**MA 2019**	Circuit training (Physical activity coaching including a programme) V No intervention	8 sessions, 1x week for 8 weeks. Each session was 140-180 minutes	People with SCI	14/14	Vo2 Peak	Some Concerns of Risk of Bias PEDro = 5/10

CONSENSUS-BASED OPINION STATEMENTS

	FES cycling (v no intervention) on cardiorespiratory fitness in people with SCI		
**P**	People with SCI	**Evidence recommendation**<br>○ No evidence recommendation<br>Reason: No RCTs	**Strong opinion statement FOR**<br>FES cycling should be provided to improve cardiorespiratory fitness in people with SCI.
**I**	FES cycling		
**C**	No intervention	**Consensus-based opinion statement**<br>● Strong for (77%)	
**O**	Cardiorespiratory Fitness		

	Combined arm cranking and leg cycling (plus or minus Electrical Stimulation) v no intervention to improve cardiorespiratory fitness in people with SCI		
**P**	People with SCI	**Evidence recommendation**<br>○ No evidence recommendation<br>Reason: No RCTs	**Strong opinion statement FOR**<br>Combined arm cranking and leg cycling (plus or minus Electrical Stimulation) should be provided to improve cardiorespiratory fitness in people with SCI
**I**	Combined arm cranking and leg cycling (plus or minus Electrical Stimulation)		
**C**	No intervention	**Consensus-based opinion statement**<br>● Strong for (89%)	
**O**	Cardiorespiratory Fitness		

	Individual or team sports (v no intervention) on cardiovascular health in people with SCI		
**P**	People with SCI	**Evidence recommendation**<br>◯ No evidence recommendation<br>Reason: No RCTs	**Strong opinion statement <u>FOR</u>**<br>Individual or team sports should be available to improve cardiovascular health in people with SCI.
**I**	Individual or team sports		
**C**	No intervention	**Consensus-based opinion statement**<br>● Strong for (96%)	
**O**	Cardiorespiratory Fitness		

	Wheelchair pushing (v no intervention) on cardiorespiratory fitness in people with SCI who are wheelchair dependent					
**P**	People with SCI who are wheelchair dependent	**Evidence recommendation**<br>◯ No evidence recommendation<br>Reason: No recommendation due to insufficient or inconclusive evidence.		**Weak opinion statement <u>FOR</u>**<br>Wheelchair pushing may be provided to improve cardiorespiratory fitness in people with SCI who are wheelchair dependent.<br><br>Clinical note: Wheelchair pushing for cardiorespiratory fitness may not be appropriate for people with shoulder pain or overuse.		
**I**	Wheelchair pushing					
**C**	No intervention	**Consensus-based opinion statement**<br>● Weak for (83%)				
**O**	Cardiorespiratory Fitness (Vo2 peak)					
	**SUMMARY**	1 RCT[81]		Mean Difference (95% CI)<br>0 (-0.2 to 0.1)		
	**GRADE**<br>Very low certainty<br>⊕◯◯◯	**Risk of bias**<br>Serious	**Inconsistency**<br>Serious	**Imprecision**<br>Very serious	**Indirectness**<br>Serious	**Publication bias**<br>Serious

Study or Subgroup	Experimental Mean	SD	Total	Control Mean	SD	Total	Mean Difference IV, Fixed, 95% CI
van der Scheer et al, 2016	-0.07	0.21783	12	-0.05	0.18088	13	-0.02 [-0.18, 0.14]

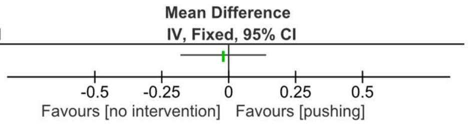

WHEELCHAIR PUSHING ON CARDIORESPIRATORY FITNESS: GRADE Evidence to Decision						
**PROBLEM**	No	Probably no	Probably yes	**Yes**		Don't know
**DESIRABLE EFFECTS**	Trivial	Small	Moderate	Large		**Don't know**
**UNDESIRABLE EFFECTS**	Large	Moderate	Small	Trivial		**Don't know**
**CERTAINTY OF EVIDENCE**	**Very low**	Low	Moderate	High		No included studies
**HOW MUCH PEOPLE VALUE THE MAIN OUTCOME**	Important uncertainty or variability	Possibly important uncertainty or variability	**Probably no important uncertainty or variability**	No important uncertainty or variability		
**BALANCE OF EFFECTS**	Favours the Control	Probably favours the Control	**Does not favour either the intervention (I) or the comparison (C)**	Probably favours the I	Favours the I	Don't know
**RESOURCES REQUIRED**	Large costs	Moderate costs	**Negligible costs and savings**	Moderate savings	Large savings	Don't know
**CERTAINTY OF EVIDENCE OF REQUIRED RESOURCES**	Very low	Low	Moderate	High		**No included studies**
**COST EFFECTIVENESS**	Favours the comparison	Probably favours the comparison	Does not favour either the intervention or the comparison	Probably favours the intervention	Favours the intervention	**No included studies**
**EQUITY**	Reduced	Probably reduced	**Probably no impact**	Probably increased	Increased	Don't know
**ACCEPTABILITY**	No	Probably no	Probably yes	**Yes**		Don't know
**FEASIBILITY**	No	Probably no	Probably yes	**Yes**		Don't know

WHEELCHAIR PUSHING ON CARDIORESPIRATORY FITNESS: Randomised Controlled Trial Details

STUDY	COMPARISON	DOSAGE/DETAILS	PARTICIPANTS	N (RX/C)	OUTCOME	ROB 2 PEDRO
**VAN DER SCHEER 2016**	Wheelchair treadmill propulsion V No intervention	Wheelchair treadmill propulsion, twice a week (30 mins) for 16 weeks (30-40% HRR)	C4 to L5 SCI	12/13	Vo2 Peak	Some Concerns of Risk of Bias PEDro = 7

Appendix 1: Additional administration details for the Guidelines

Guideline Management Committee

A Guideline Management Committee was convened to oversee the development of the Australian and New Zealand Physiotherapy Guidelines for people with SCI. The committee was assembled by the chair and co-chair. The purpose of the Guideline Management Committee was oversight and governance of the project.

In scope:

- Oversee the process, management, governance and rollout of the Physiotherapy Clinical Guidelines Project.
- Provide recommendations about the process, management, governance and rollout of the Physiotherapy Clinical Guidelines.

Out of scope:

- Decisions about the clinical questions covered in the clinical guidelines. This was the responsibility of the Guideline Development Group.
- Decisions about the recommendations within the clinical practice guidelines for Physiotherapists and Consumers. This was the responsibility of the Guideline Development Group.
- Approval of the clinical guidelines for physiotherapists and consumers. This was the responsibility of the Guideline Development Group.

Membership:
Membership of the Guideline Management Committee included a chairperson, representatives of the guideline funding agency, a consumer and content experts (clinical guidelines, SCI physiotherapy, SCI research/evidence). Key responsibilities of the Project management committee included (Adapted from NICE 2014)[82]

All members:

- Contributed to meetings.
- Declared relevant conflicts of interest.
- Contributed and provided strategies for resolution of issues within the project as/if they occurred.
- Considered and contributed to suggestions about parties to review and provided feedback on the guidelines.
- Approved the terms of reference.

Chair:

- Contributed to drafting of terms of reference.
- Facilitated participation of Project management committee members.
- Managed conflicts of interest.
- Updated the Project management committee on project milestones and developments between meetings.
- Organised workflow of the Project management committee should tasks be required.

Consumers:

- Provided advice and recommendations about consumer issues and concerns related to the project as/if they occurred.

Content Experts:

- Applied their knowledge to assist the group to carry out the project to the highest standard possible.
- Provided advice on best practice in the areas in which they had experience and expertise.
- Assisted the Project management committee in understanding best practice in the area which they had experience and expertise.

Guideline Management Committee members

Name	Affiliation
A/Prof Joanne Glinsky (Chair and Project coordinator)	Kolling Institute, Faculty of Medicine and Health, The University of Sydney, Sydney, NSW, Australia<br>John Walsh Centre for Rehabilitation Research, Northern Sydney Local Health District, Sydney, NSW, Australia
Professor Lisa Harvey (Co-chair and Project lead)	Kolling Institute, Faculty of Medicine and Health, The University of Sydney, Sydney, NSW, Australia<br>John Walsh Centre for Rehabilitation Research, Northern Sydney Local Health District, Sydney, NSW, Australia
Mr Nick Taylor	iCare, NSW, Australia
Ms Jacqueline Scott	iCare, NSW, Australia
Ms Wendy Harris	No affiliation
Ms Marsha Ben	Royal Rehab, Sydney, NSW, Australia
Professor Coralie English	School of Health Sciences, University of Newcastle, NSW, Australia
Mr Mark McDonald	Victorian Spinal Cord Service, Austin Health, Victoria, Australia
Ms Emilie Gollan	Princess Alexandra Hospital, Metro South health, Qld, Australia
Ms Christina Kerr	National Injury Insurance Scheme, Queensland
Ms Jacqueline Woerner	Transport Accident Commission, Queensland

Guideline Development Committee

A Guideline Development Committee was convened to develop the Australian and New Zealand Physiotherapy Guidelines for people with SCI. The committee was assembled by the chair and co-chair. The purpose of the Guideline Development Committee was to decide on the clinical questions contained within the guideline and provide recommendations about these clinical questions.

In scope:

- Decisions about the treatments and clinical questions covered in the clinical guidelines.
- Decisions about the recommendations within the clinical practice guidelines for Physiotherapists and Consumers.
- Approval of the clinical guidelines for physiotherapists and consumers.
- Provided recommendations about the implementation of the guidelines.

Out of scope:

- Management of the Physiotherapy Clinical Guidelines Project. This was the responsibility of the Project Management Committee.

Membership:

Membership of the Guideline Development Committee included a chairperson, content experts across the continuum of care (including physiotherapists, academics, exercise physiologists) and consumers with knowledge of provision of evidence-based care. Key responsibilities of the steering committee members included (adapted from NICE 2014)[83]

All members:

- Contributed to meetings
- Declared relevant conflicts of interest
- Contributed to decisions about the clinical questions contained within the guidelines.
- Voted on recommendations to be contained within the guidelines
- Contributed and provided strategies for resolution of issues with the project as/if they occurred.
- Approved terms of reference.

Chair:

- Contributed to drafting of terms of reference.
- Facilitated participation of committee members.
- Managed conflicts of interest.
- Updated the committee on project milestones and developments between meetings.
- Organised workflow for tasks as required.

Content Experts (Physiotherapists, exercise physiologist, academics):

- Applied their knowledge to assist the group to carry out the project to the highest standard possible.

- Provided advice on best practice in the areas in which they had experience and expertise.
- Assisted in understanding best practice in the areas which had experience and expertise.

Consumers:

- Provided advice and recommendations about consumer issues and concerns related to the project as/if they occurred.

Guideline Development Committee members – Rehabilitation

Name	Affiliation
A/Prof Joanne Glinsky (Chair and Project coordinator)	Kolling Institute, Faculty of Medicine and Health, The University of Sydney, Sydney, NSW, Australia<br>John Walsh Centre for Rehabilitation Research, Northern Sydney Local Health District, Sydney, NSW, Australia
Professor Lisa Harvey (Co-chair and Project lead)	Kolling Institute, Faculty of Medicine and Health, The University of Sydney, Sydney, NSW, Australia<br>John Walsh Centre for Rehabilitation Research, Northern Sydney Local Health District, Sydney, NSW, Australia
Ms Keira Ralston (Physiotherapist member)	Kolling Institute, Faculty of Medicine and Health, The University of Sydney, Sydney, NSW, Australia<br>John Walsh Centre for Rehabilitation Research, Northern Sydney Local Health District, Sydney, NSW, Australia
Ms Jackie Chu (Meeting co-ordinator)	Kolling Institute, Faculty of Medicine and Health, The University of Sydney, Sydney, NSW, Australia<br>John Walsh Centre for Rehabilitation Research, Northern Sydney Local Health District, Sydney, NSW, Australia
Mr Mark McDonald (Victorian lead)	Victorian Spinal Cord Service, Austin Health, Victoria, Australia
Ms Emilie Gollan (Queensland co-lead)	Princess Alexandra Hospital, Metro South health, Qld, Australia
Ms Brooke Wadsworth (Queensland co-lead)	Princess Alexandra Hospital, Metro South health, Qld, Australia
Ms Deanne Wilson (South Australian lead)	Spinal Outreach Team, Central Adelaide Local Health Service, South Australia

Name	Affiliation
Ms Marsha Ben (Physiotherapist member)	Royal Rehab, Sydney, Australia
Ms Jacqui White (Physiotherapist member)	Royal Rehab, Sydney, Australia
Mr Adrian Byak (Physiotherapist member)	Optimise your level physiotherapy, Sydney, Australia
Dr Jonathon Tang (Consumer and Medical member)	Northern Sydney Local Health District, Sydney, NSW, Australia
Ms Donna Rainey (Physiotherapist member)	Royal Rehab, Sydney, Australia
Mr Jason Redhead (Physiotherapist member)	Royal Rehab, Sydney, Australia
Ms Amanda Haber (Physiotherapist member)	Royal Rehab, Sydney, Australia
Dr Liz Bye (Physiotherapist member)	Neuroscience Research Australia, Sydney Australia
Ms Fernanda Di Natal (Physiotherapist member)	Prince of Wales Hospital, Southeastern Sydney Local Health District, Sydney, Australia
Dr Che Fornusek (Academic member)	University of Sydney, Sydney, Australia
Ms Lydia Chen (Physiotherapist member)	Royal North Shore Hospital, Northern Sydney Local Health District, Sydney, NSW, Australia
Ms Sophia Denis (Physiotherapist member)	Prince of Wales Hospital, Southeastern Sydney Local Health District, Sydney, Australia
Mr Jai Peach (Physiotherapist member)	Princess Alexandra Hospital, Metro South health, Qld, Australia
Dr Camila Quel De Oliveira (Physiotherapist and academic member)	University of Technology, Sydney, Australia
Ms Sheelagh Donahoe (Physiotherapist member)	Hampstead Rehabilitation Centre, Central Adelaide Local Health Network, South Australia
Dr Jennifer Dunn (Physiotherapist and academic member)	Orthopaedic Surgery and Musculoskeletal Medicine, University of Otago, Christchurch, New Zealand
Dr Jo Nunnerley (Physiotherapist and academic member)	Orthopaedic Surgery and Musculoskeletal Medicine, University of Otago, Christchurch, New Zealand
Dr Verna Stavric (Physiotherapist and academic member)	Auckland University of Technology, Auckland, New Zealand

Name	Affiliation
Ms Maree Waters (Physiotherapist member)	Middlemore Hospital, Manukau District Health Board, Auckland, New Zealand
Ms Jennie McCorkell	My Turn Rehabilitation, Queensland, Australia
Mr Anthony Nakhle (Physiotherapist member)	Spinal Life Australia, Queensland, Australia
Ms Lucy Maughan (Physiotherapist member)	Queensland Spinal Cord Injuries Service, Queensland, Australia
Ms Leanne Rees (Physiotherapist member)	Victorian Spinal Cord Service, Austin Health, Victoria, Australia
Ms Janet McCarthy (Physiotherapist member)	Victorian Spinal Cord Service, Austin Health, Victoria, Australia
Ms Mel Kotze (Physiotherapist member)	Victorian Spinal Cord Service, Austin Health, Victoria, Australia

Guideline Development Committee members - Respiratory

Name	Affiliation
A/Prof Joanne Glinsky (Chair and Project coordinator)	Kolling Institute, Faculty of Medicine and Health, The University of Sydney, Sydney, NSW, Australia<br>John Walsh Centre for Rehabilitation Research, Northern Sydney Local Health District, Sydney, NSW, Australia
Professor Lisa Harvey (Co-chair and Project lead)	Kolling Institute, Faculty of Medicine and Health, The University of Sydney, Sydney, NSW, Australia<br>John Walsh Centre for Rehabilitation Research, Northern Sydney Local Health District, Sydney, NSW, Australia
Ms Keira Ralston (Physiotherapist member)	Kolling Institute, Faculty of Medicine and Health, The University of Sydney, Sydney, NSW, Australia<br>John Walsh Centre for Rehabilitation Research, Northern Sydney Local Health District, Sydney, NSW, Australia
Ms Jackie Chu (Meeting coordinator)	Kolling Institute, Faculty of Medicine and Health, The University of Sydney, Sydney, NSW, Australia<br>John Walsh Centre for Rehabilitation Research, Northern Sydney Local Health District, Sydney, NSW, Australia

Name	Affiliation
Professor David Berlowitz (Academic member)	University of Melbourne, Victoria, Australia<br>Austin Health, Victoria, Australia
Ms Jack Ross (Physiotherapist member)	Victorian Spinal Cord Service, Austin Health, Victoria, Australia
Mr Mark McDonald (Physiotherapist member)	Victorian Spinal Cord Service, Austin Health, Victoria, Australia
Ms Sara Calthorpe (Physiotherapist member)	Victorian Spinal Cord Service, Austin Health, Victoria, Australia
Ms Jacqui Agostinello (Physiotherapist member)	Victorian Spinal Cord Service, Austin Health, Victoria, Australia
Ms Emilie Gollan (Physiotherapist member)	Princess Alexandra Hospital, Metro South health, Qld, Australia
Ms Brooke Wadsworth (Physiotherapist member)	Princess Alexandra Hospital, Metro South health, Qld, Australia
Ms Deanne Wilson (Physiotherapist member)	Spinal Outreach Team, Central Adelaide Local Health Service, South Australia
Dr Liz Bye (Physiotherapist and academic member)	Neuroscience Research Australia, Sydney Australia
Ms Lydia Chen (Physiotherapist member)	Royal North Shore Hospital, Northern Sydney Local Health District, Sydney, NSW, Australia
Ms Sophia Denis (Physiotherapist member)	Prince of Wales Hospital, Southeastern Sydney Local Health District, Sydney, Australia
Mr Jai Peach (Physiotherapist member)	Princess Alexandra Hospital, Metro South health, Qld, Australia
Mr Tony McDonald (Physiotherapist member)	Hampstead Rehabilitation Centre, Central Adelaide Local Health Network, South Australia
Dr Verna Stavric (Observer)	Auckland University of Technology, Auckland, New Zealand
Ms Maree Waters (Physiotherapist member)	Middlemore Hospital, Manukau District Health Board, Auckland, New Zealand
Mr Anthony Nakhle (Observer)	Spinal Life Australia, Queensland, Australia
Ms Leanne Rees (Observer)	Victorian Spinal Cord Service, Austin Health, Victoria, Australia
Ms Yahlina Bhamji (Physiotherapist member)	Middlemore Hospital, Manukau District Health Board, Auckland, New Zealand

Name	Affiliation
Ms Joanna Mather (Physiotherapist member)	Middlemore Hospital, Manukau District Health Board, Auckland, New Zealand
Ms Liesl Davis (Physiotherapist member)	Royal North Shore Hospital, Northern Sydney Local Health District, Sydney, NSW, Australia
Ms Lynn Blecher (Physiotherapist member)	Prince of Wales Hospital, Southeastern Sydney Local Health District, Sydney, Australia
Ms Helen Patterson (Physiotherapist member)	Royal North Shore Hospital, Northern Sydney Local Health District, Sydney, NSW, Australia
Mr Mario Dcruz (Consumer and medical member)	No affiliation

Conflict of interest

This guideline has been produced in accordance with the processes outlined in the Australian and New Zealand Physiotherapy Guidelines for people with SCI Conflict of Interest (COI) Policy. COI disclosures will be available on the website.

Pre- Guideline engagement

Pre-guideline engagement of consumers, physiotherapists and stakeholders informed PICO questions and development of the guideline. The full report will be available on the guidelines website.

Public consultation

This guideline was released for public consultation on 20/9/22. Public consultation closed on 10/10/22. Key stakeholders identified by the guideline management and development committees were invited to make submissions. De-identified submissions and responses will be publicly available on completion of the process.

Appendix 2: Additional technical details for the Guidelines

PICO questions

Questions that included Participants, Intervention, Comparison and Outcome (PICO) were decided by the guideline development group prior to commencement of the process. PICO questions were added or changed over the course of the process by the guideline development group. The PICO questions considered in this review are detailed below:

> Respiratory muscle training v no intervention on respiratory muscle strength in people with SCI who have respiratory muscle weakness.
>
> Abdominal binders in sitting v no intervention on lung volumes in people with SCI who have respiratory muscle weakness.
>
> Supine v sitting on lung volumes in people with SCI who have abdominal muscle paralysis (full or partial)
>
> Intermittent application of positive pressure devices v no intervention on lung volume in non-ventilated people with acute SCI who have respiratory muscle weakness.
>
> Additional application of positive pressure devices v no intervention on lung volume in ventilated people SCI
>
> Deep breathing exercises v no intervention on lung volumes in people with SCI who have respiratory muscle weakness.
>
> Air stacking v no intervention on lung volumes in people with SCI who have respiratory muscle weakness.
>
> Abdominal FES v no intervention on lung volumes in people with SCI who have respiratory muscle weakness.
>
> Targeted postural drainage v no intervention on secretion clearance in people with SCI who have respiratory muscle weakness
>
> Manually assisted cough v no intervention on secretion clearance in people with SCI who have abdominal muscle paralysis (full or partial)
>
> Mechanically assisted cough (insufflation/exsufflation) v no intervention on secretion clearance in people with SCI who have respiratory muscle weakness
>
> Mechanically assisted cough (insufflation/exsufflation) plus manually assisted cough v no intervention on secretion clearance in people with SCI who have abdominal muscle paralysis (full or partial)
>
> Percussion and vibration v no intervention on secretion clearance in people with SCI who have respiratory muscle weakness

Abdominal binders v no intervention to improve cough in people with SCI who have abdominal muscle paralysis (full or partial)

Abdominal FES v no intervention on stimulated cough in people with SCI who have abdominal paralysis (partial or full).

Positive expiratory pressure devices v no intervention on secretion clearance in people with SCI who have expiratory muscle weakness.

Abdominal binders v no intervention on postural hypotension in people with SCI

Manual wheelchair skills training v no intervention on wheelchair skills in people with SCI

Virtual reality sitting training v no intervention on ability to sit in people with SCI

Power wheelchair skills training v no intervention on power wheelchair skills in people with SCI who are dependent on a power wheelchair for mobility

Bed mobility v no intervention on ability to move in bed in people with SCI

Sitting training v no intervention on ability to sit in people with SCI and motor function in the lower limbs

Sitting training v no intervention on ability to sit in people with SCI and paralysis of the lower limbs/trunk

Transfer training v no intervention on ability to transfer in people with SCI

Vertical transfer training v no intervention on ability to vertically transfer in people with SCI who are wheelchair dependent

Sit to stand training v no intervention on ability to move from sit to stand in people with SCI and motor function in the lower limbs

Standing training v no intervention on ability to stand in people with SCI and motor function in the lower limbs

Stair training v no intervention to improve the ability to climb stairs in people with SCI and motor function in the lower limbs

Hand function training v no intervention to improve hand function in people with tetraplegia

Robotic Upper limb training v no intervention to improve upper limb function in people with tetraplegia

Walking training vs no intervention to improve walking in people with SCI and motor function in the lower limbs. Walking training can include:

- Overground gait training
- Treadmill gait training (+/- body weight support)
- Treadmill gait training with electrical stimulation (+/- body weight support)
- Overground gait training and electrical stimulation
- Robotic overground gait training

- Robotic treadmill gait training
- Conventional therapy (package of interventions including gait training)
- Gait training with orthotics

Conventional therapy (package of interventions including gait training) vs treadmill gait training (with or without body weight support) to improve walking in people with SCI and motor function in the lower limbs

Upper limb training, Hand function training and FES v no intervention to improve hand function in people with tetraplegia

Upper limb virtual reality v no intervention to improve hand function in people with tetraplegia

Tenodesis splinting v no intervention to improve a tenodesis grip in people with C6 or C7 tetraplegia

Overground gait training v Robotic gait training to improve walking in people with SCI and potential for upright mobility

Overground gait training vs Treadmill gait training (with or without body weight support) to improve walking in people with SCI and motor function in the lower limbs

Treadmill gait training (with or without body weight support) vs Robotic gait training to improve walking in people with SCI and motor function in the lower limbs

Hydrotherapy v land therapy to improve mobility for people with SCI

Gait training (any type) v no intervention to improve walking for people with no motor function in the lower limbs

TENS v no intervention to treat pain in people with SCI

Education to avoid overuse and trauma v no intervention to prevent and treat shoulder pain in people with SCI

Shoulder exercises v no intervention to treat shoulder pain in people with SCI

Massage vs no intervention to treat pain in people with SCI

Positioning v no intervention to prevent shoulder pain in people with SCI

Passive movements v no intervention to prevent or treat shoulder pain

Equipment to support the shoulder v no intervention to prevent shoulder subluxation in people with SCI at risk of shoulder subluxation

Neuromuscular electrical stimulation v no intervention to prevent shoulder subluxation in people with SCI at risk of shoulder subluxation

Shoulder support devices/braces v no intervention to prevent shoulder subluxation in people with SCI

Neuromuscular electrical stimulation v no intervention to treat shoulder subluxation in people with SCI

Shoulder support devices/braces v no intervention to treat shoulder subluxation in people with SCI

Long duration stretch v no intervention on joint mobility in people with SCI

Active Assisted Exercise v no intervention on prevention of loss of joint mobility in people with SCI who are at risk of contracture

Active Assisted Exercise v no intervention on treatment of loss of joint mobility in people with SCI

Passive standing v no intervention on joint mobility in people with SCI and paralysed lower limbs

Serial casting v no intervention on joint mobility in people with SCI

Hand splinting versus no intervention on prevention of hand contractures in people with tetraplegia

Hand splinting versus no intervention on treatment of hand contractures in people with tetraplegia

Upper and lower limb splinting versus no intervention on prevention of contractures in people with SCI who are at risk of contracture

Passive range of motion exercises v no intervention on joint mobility in people with SCI

Passive range of motion exercises v no intervention on spasticity in people with SCI

Passive standing v no intervention on spasticity in people with SCI

FES cycling v no intervention on spasticity in people with SCI

Vibration v no intervention on spasticity in people with SCI

Passive standing v no intervention on bone mineral density

Elevation v no intervention on swelling in people with SCI

Neuromuscular electrical stimulation v no intervention on swelling

Lymphatic massage v no intervention on swelling

ES cycling v no intervention on swelling

Strength training v no intervention to improve voluntary strength of non-paralysed muscles in people with SCI

Strength training v no intervention on voluntary strength of partially paralysed muscles

FES cycling v no intervention to decrease atrophy in people with SCI and paralysis of the lower limbs

Electrical stimulation alone v no intervention on voluntary strength of partially paralysed muscles in people with SCI

Strength training combined with electrical stimulation v no intervention on voluntary strength of partially paralysed muscles in people with SCI

Whole body vibration v no intervention on voluntary strength in people with SCI

Arm cranking v no intervention on cardiorespiratory fitness in people with SCI

Hand Cycling v no intervention on cardiorespiratory fitness in people with SCI

Circuit training v no intervention on cardiorespiratory fitness in people with SCI

FES cycling v no intervention to improve cardiorespiratory fitness in people with SCI

Individual or team sports v no intervention to improve cardiovascular health in people with SCI

Wheelchair pushing v no intervention on cardiorespiratory fitness in people with SCI who are wheelchair dependent

Decision making process of the Guideline Development Committee

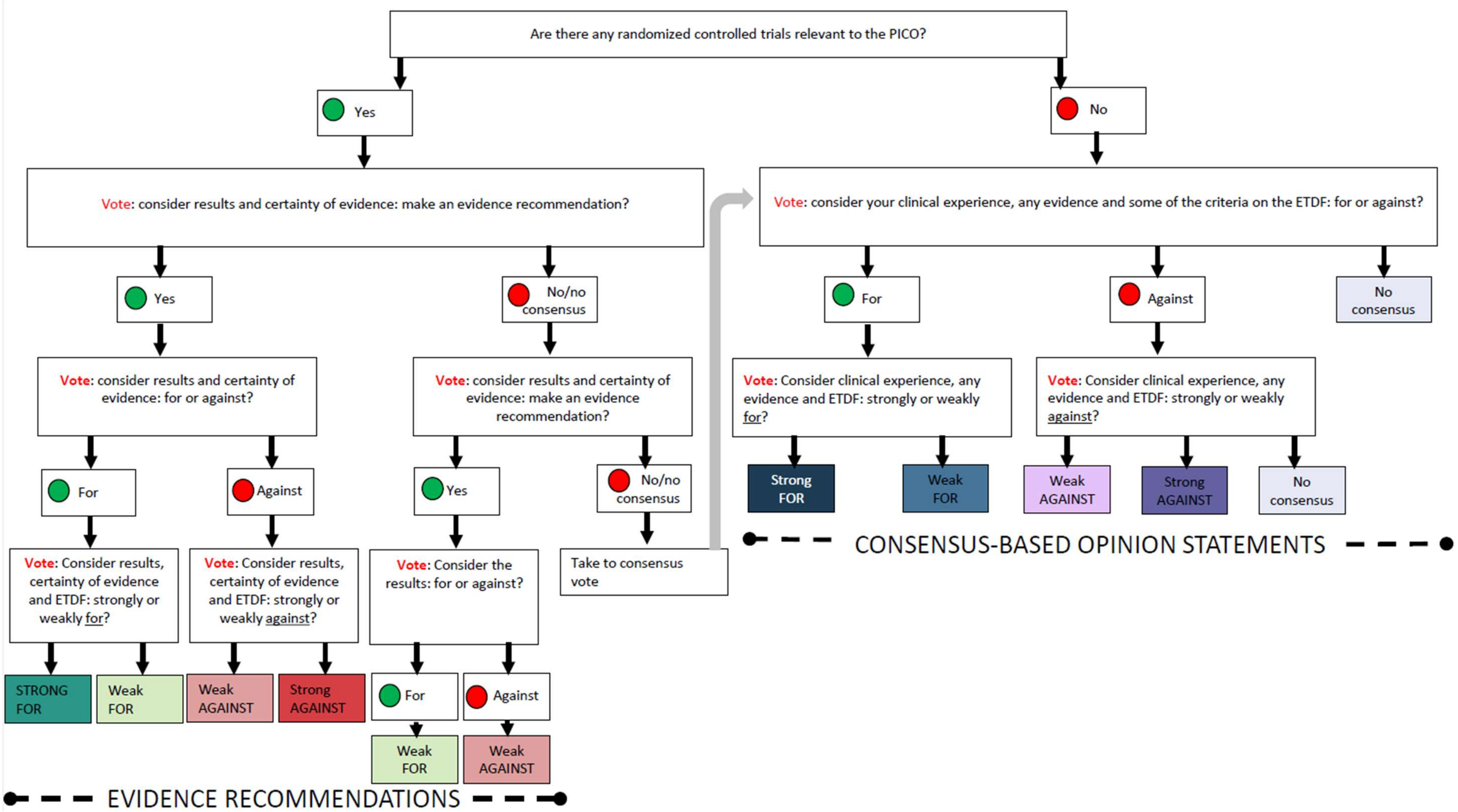

Search strategies

Database: EBM Reviews - Cochrane Central Register of Controlled Trials <July 2020>

Search Strategy:

----

1 exp Spinal Cord Injuries/ (1659)

2 Quadriplegia/ (190)

3 Paraplegia/ (205)

4 (myelopath* adj3 (trauma* or post-trauma*)).ti,ab. (3)

5 ((spine or spinal or vertebra*) adj3 (fracture* or trauma* or injur* or damag*)).ti,ab. (6670)

6 (spinal cord adj3 (injur* or contus* or lacerat* or transect* or trauma* or isch?emia)).ti,ab. (3232)

7 (parapleg* or quadripleg* or tetrapleg*).ti,ab. (886)

8 (central spinal cord adj3 syndrome).ti,ab. (1)

9 SCI.ti,ab. (2218)

10 1 or 2 or 3 or 4 or 5 or 6 or 7 or 8 or 9 (8073)

Database: Embase Classic+Embase <1947 to 2020 August 13>

Search Strategy:

----

1 exp spinal cord injury/ (80239)

2 exp spinal cord ischemia/ (4284)

3 exp paraplegia/ (27814)

4 (myelopath* adj3 (trauma* or post trauma*)).ti,ab. (229)

5 ((spine or spinal or vertebra*) adj3 (fracture* or trauma* or injur* or damag*)).ti,ab. (96510)

6 (spinal cord adj3 (injur* or contus* or lacerat* or transect* or trauma* or isch?emia)).ti,ab. (59016)

7 (central spinal cord adj3 syndrome).ti,ab. (12)

8 SCI.ti,ab. (48864)

9 (parapleg* or quadripleg* or tetrapleg*).ti,ab. (33783)

10 or/1-9 (188718)

11 Clinical trial/ (1001390)

12 Randomized controlled trial/ (618265)

13 Randomization/ (87877)

14 Single blind procedure/ (39886)

15 Double blind procedure/ (177567)

16 Crossover procedure/ (64374)

17 Placebo/ (364157)

18 Randomi?ed controlled trial$.tw. (234769)

19 Rct.tw. (38172)

20 Random allocation.tw. (2134)

21 Randomly allocated.tw. (36077)

22 Allocated randomly.tw. (2600)

23 (allocated adj2 random).tw. (983)

24 Single blind$.tw. (25493)

25 Double blind$.tw. (216990)

26 ((treble or triple) adj blind$).tw. (1225)

27 Placebo$.tw. (316762)

28 Prospective study/ (622386)

29 or/11-28 (2275463)

30 Case study/ (80602)

31 Case report.tw. (446697)

32 Abstract report/ or letter/ (1158981)

33 or/30-32 (1675336)

34 Animal experiment/ not (human experiment/ or human/) (2264248)

35 33 or 34 (3894371)

36 (10 and 29) not 35 (14857)

37 limit 36 to conference abstract status (3250)

38 36 not 37 (11607)

Database: Ovid MEDLINE(R) ALL <1946 to August 13, 2020>

Search Strategy:

----

1 exp spinal cord injuries/ or central cord syndrome/ or Spinal Cord Compression/ (48726)

2 exp Spinal Cord/ and exp "Wounds and Injuries"/ (9945)

3 exp paraplegia/ or quadriplegia/ (19569)

4 (myelopath* adj3 (trauma* or post-trauma*)).ti,ab. (180)

5 ((spine or spinal or vertebra*) adj3 (fracture* or trauma* or injur* or damag*)).ti,ab. (69962)

6 (spinal cord adj3 (injur* or contus* or lacerat* or transect* or trauma* or isch?emia)).ti,ab. (44866)

7 SCI.ti,ab. (34659)

8 (central spinal cord adj3 syndrome).ti,ab. (10)

9 (parapleg* or quadripleg* or tetrapleg*).ti,ab. (23126)

10 or/1-9 (132080)

11 Randomized controlled trials as Topic/ (135270)

12 Randomized controlled trial/ (511146)

13 Random allocation/ (103360)

14 Double blind method/ (159244)

15 Single blind method/ (28900)
16 Clinical trial/ (524255)
17 exp Clinical Trials as Topic/ (344404)
18 or/11-17 (1178511)
19 (clinic$ adj trial$1).tw. (370575)
20 ((singl$ or doubl$ or treb$ or tripl$) adj (blind$3 or mask$3)).tw. (173714)
21 Placebos/ (35022)
22 Placebo$.tw. (216979)
23 Randomly allocated.tw. (28975)
24 (allocated adj2 random).tw. (799)
25 or/19-24 (639473)
26 18 or 25 (1458119)
27 Case report.tw. (314378)
28 Letter/ (1094253)
29 Historical article/ (359648)
30 Review of reported cases.pt. (0)
31 Review, multicase.pt. (0)
32 or/27-31 (1752210)
33 exp animals/ not humans.sh. (4725270)
34 26 not (32 or 33) (1333215)
35 34 and 10 (7555)

References

1. Schünemann H, Brozek J, Guyatt G, Oxman A. The Grade handbook (2013). Last accessed 20/6/22 at URL https://gdt.gradepro.org/app/handbook/handbook.html
2. Kirshblum SC, Burns SP, Biering-Sorensen F, Donovan W, Graves DE, Jha A, Johansen M, Jones L, Krassioukov A, Mulcahey MJ, Schmidt-Read M, Waring W. International standards for neurological classification of spinal cord injury (revised 2011). J Spinal Cord Med. 2011 Nov;34(6):535-46.
3. National Health and Medical Research Council. Procedures and requirements for meeting the 2011 NHMRC standard for clinical practice guidelines. Melbourne: National Health and Medical Research Council; 2011.
4. PEDro Physiotherapy Evidence Database www.PEDro.org Last accessed 24/9/22.
5. Sterne JAC, Savović J, Page MJ, Elbers RG, Blencowe NS, Boutron I, Cates CJ, Cheng HY, Corbett MS, Eldridge SM, Emberson JR, Hernán MA, Hopewell S, Hróbjartsson A, Junqueira DR, Jüni P, Kirkham JJ, Lasserson T, Li T, McAleenan A, Reeves BC, Shepperd S, Shrier I, Stewart LA, Tilling K, White IR, Whiting PF, Higgins JPT. RoB 2: a revised tool for assessing risk of bias in randomised trials. BMJ. 2019 Aug 28;366:l4898.
6. Liaw MY, Lin MC, Cheng PT, et al. Resistive inspiratory muscle training: its effectiveness in patients with acute complete cervical cord injury. Archives of Physical Medicine and Rehabilitation 2000 Jun;81(6):752-756.
7. Litchke L, Lloyd L, Schmidt E, et al. Comparison of two concurrent respiratory resistance devices on pulmonary function and time trial performance of wheelchair athletes. Therapeutic Recreation Journal 2011;45(2):147-159.
8. Litchke LG, Russian CJ, Lloyd LK, et al. Effects of respiratory resistance training with a concurrent flow device on wheelchair athletes. The Journal of Spinal Cord Medicine 2008;31(1):65-71.
9. Loveridge B, Badour M, Dubbo H. Ventilatory Muscle Endurance Training in Quadriplegics; effects on breathing pattern. Paraplegia 1989; 27: 329-339.
10. Mueller G, Hopman MTE and Perret C. Comparison of respiratory muscle training methods in individuals with motor complete tetraplegia. Topics in Spinal Cord Injury Rehabilitation 2012;18(2):118-121.
11. Postma K, Haisma JA, Hopman MTE, et al. Resistive inspiratory muscle training in people with spinal cord injury during inpatient rehabilitation: a randomized controlled trial. Physical Therapy 2014 ;94(12):1709-1719 2014.
12. Soumyashree S, Kaur J. Effect of inspiratory muscle training (IMT) on aerobic capacity, respiratory muscle strength and rate of perceived exertion in paraplegics. Journal of spinal cord medicine 2018: 1-7.
13. West CR, CR, Taylor BJ, Campbell IG, Romer LM. Effects of inspiratory muscle training on exercise responses in Paralympic athletes with cervical spinal cord injury. Scandinavian journal of medicine & science in sports 2014; 24: 764.

14. Boswell-Ruys CL, Lewis CRH, Wijeysuriya NS, et al. Impact of respiratory muscle training on respiratory muscle strength, respiratory function and quality of life in individuals with tetraplegia: a randomised clinical trial. Thorax 2020;75:279-288.
15. Roth EJ, Stenson KW, Powley S, Oken J, Primack S, Nussbaum SB, Berkowitz M. Expiratory muscle training in spinal cord injury: a randomized controlled trial. Arch Phys Med Rehabil. 2010 Jun;91(6):857-61.
16. Boaventura, C. D.Gastaldi, A. C.Silveira, J. M.Santos, P R.Guimaraes, R. C.De, L. L. C. Effect of an abdominal binder on the efficacy of respiratory muscles in seated and supine tetraplegic patients. Physiotherapy 2003 May;89(5):290-295.
17. Wadsworth, B. M. Haines, T. P. Cornwell, P. L. Rodwell, L. T. Paratz, J. D. An abdominal binder improves lung volumes and voice in people with tetraplegic spinal cord injury. Archives of Physical Medicine and Rehabilitation 2012 Dec;93(12):2189-2197.
18. Hart, N. Laffont, I.de la Sota, A. P.Lejaille, M.Macadou, G.Polkey, M. I.Denys, P.Lofaso, F. Respiratory effects of combined truncal and abdominal support in patients with spinal cord injury. Archives of Physical Medicine and Rehabilitation 2005 Jul;86(7):1447-1451
19. Bodin P, Fagevik Olsen M, Bake B, Kreuter M. Effects of abdominal binding on breathing patterns during breathing exercises in persons with tetraplegia. Spinal Cord 2005; 43: 117–122.
20. Goldman JM, Rose LS, Williams SJ, Silver JR, Denison DM. Effect of abdominal binders on breathing in tetraplegic patients. Thorax 1986; 41: 940–945.
21. Laffont I, Bensmail D, Lortat-Jacob S, et al. Intermittent positive-pressure breathing effects in patients with high spinal cord injury. Archives of physical medicine and rehabilitation 2008; 89: 1575-1579.
22. Jeong, JH, Yoo WG. Effects of air stacking on pulmonary function and peak cough flow in patients with cervical spinal cord injury. Journal of Physical Therapy Science 2015 Jun;27(6):1951-1952.
23. Cheng, P.Chen, C.Wang, C.Chung, C. Effect of neuromuscular electrical stimulation on cough capacity and pulmonary function in patients with acute cervical cord injury. Journal of Rehabilitation Medicine 2006 Jan;38(1):32-36.
24. Kirby RL, Mitchell D, Sabharwal S, et al. Manual wheelchair skills training for community-dwelling veterans with spinal cord injury: a randomized controlled trial. PLoS ONE 2016 Dec;11(12):e0168330.
25. Rice LA, Smith I, Kelleher AR, et al. Impact of the clinical practice guideline for preservation of upper limb function on transfer skills of persons with acute spinal cord injury. Archives of Physical Medicine and Rehabilitation 2013 Jul;94(7):1230-1246.
26. Worobey LA, Kirby RL, Heinemann AW et al Effectiveness of Group Wheelchair Skills Training for People With Spinal Cord Injury: A Randomized Controlled Trial. Archives of Physical Medicine and Rehabilitation 2016 Oct;97(10):1777-1784.

27. Yeo SS, Kwon JW. Wheelchair Skills Training for Functional Activity in Adults with Cervical Spinal Cord Injury. International journal of sports medicine 2018; 39: 924-928.
28. Tak S, Choi W and Lee S. Game-based virtual reality training improves sitting balance after spinal cord injury: a single-blinded, randomized controlled trial. Medical Science Technology 2015 Jun 26;56:53-59.
29. Alexeeva N et al. Comparison of training methods to improve walking in persons with chronic spinal cord injury: a randomized clinical trial. Journal of spinal cord medicine 2011; 34: 362-369.
30. Lucareli PR, Lima MO, Lima FPS, et al. Gait analysis following treadmill training with body weight support versus conventional physical therapy: a prospective randomized controlled single blind study. Spinal Cord 2011 Sep;49(9):1001-1007.
31. Piira A, Lannem AM, Sorensen M, et al. Manually assisted body-weight supported locomotor training does not re-establish walking in non-walking subjects with chronic incomplete spinal cord injury: A randomized clinical trial. Journal of rehabilitation medicine 2019; 51: 113-119.
32. Sadeghi H, Banitalebi E, Dehkordi M. The effect of body-weight-supported training exercises on functional ambulation profile in patients with paraplegic spinal cord injury. Phys Treat 2015; 4: 205–212.
33. Boswell-Ruys CL, Harvey LA, Barker JJ, et al. Training unsupported sitting in people with chronic spinal cord injuries: a randomized controlled trial. Spinal Cord 2010 Feb;48(2):138-14.
34. Harvey LA, Ristev D, Hossain MS, et al. Training unsupported sitting does not improve ability to sit in people with recently acquired paraplegia: a randomised trial. Journal of Physiotherapy 2011;57(2):83-90.
35. Beekhuizen KS, Field-Fote EC. Massed practice versus massed practice with stimulation: effects on upper extremity function and cortical plasticity in individuals with incomplete cervical spinal cord injury. Neurorehabilitation and neural repair 2005; 19: 33.
36. Harvey LA, Dunlop SA, Churilov L, et al. Early intensive hand rehabilitation is not more effective than usual care plus one-to-one hand therapy in people with sub-acute spinal cord injury ('Hands On'): a randomised trial. Journal of physiotherapy 2017; 63: 197-204.
37. Hoffman L F-FE. Effects of practice combined with somatosensory or motor stimulation on hand function in persons with spinal Cord Injury. Topics in spinal cord injury rehabilitation 2013; 19: 288.
38. Dimbwadyo-Terrer I, Gil-Agudo A, Segura-Fragoso A, et al. Effectiveness of the virtual reality system toyra on upper limb function in people with tetraplegia: a pilot randomized clinical trial. BioMed Research International 2016; BioMed Research International 2016; 6397828.
39. Lim DY, Hwang DM, Cho KH, et al. A Fully Immersive Virtual Reality Method for Upper Limb Rehabilitation in Spinal Cord Injury. Annals of rehabilitation medicine 2020. DOI: https://dx.doi.org/10.5535/arm.19181

40. Prasad S, Aikat R, Labani S, Khanna N. Efficacy of Virtual Reality in Upper Limb Rehabilitation in Patients with Spinal Cord Injury: A Pilot Randomized Controlled Trial. Asian spine journal 2018; 12: 927-934.
41. Alcobendas-Maestro M E-RAC-LRMM-GAP-MGG-VEMJL. Lokomat robotic-assisted versus overground training within 3 to 6 months of incomplete spinal cord lesion: randomized controlled trial. Neurorehabilitation and neural repair 2012; 26: 1058.
42. Esclarin-Ruz A A-MMC-LRP-MGF-SMAG-VEMJL. A comparison of robotic walking therapy and conventional walking therapy in individuals with upper versus lower motor neuron lesions: a randomized controlled trial. Archives of physical medicine and rehabilitation 2014; 95: 1023.
43. Hornby TG, Campbell DD, Zemon DH, et al. Clinical and quantitative evaluation of robotic-assisted treadmill walking to retrain ambulation after spinal cord injury. Topics in Spinal Cord Injury Rehabilitation 2005 Fall;11(2):1-17.
44. Senthilvelkumar T, Magimairaj H, Fletcher J, et al. Comparison of body weight-supported treadmill training versus body weight-supported overground training in people with incomplete tetraplegia: a pilot randomized trial [with consumer summary]. Clinical Rehabilitation 2015 Jan;29(1):42-49.
45. Yang JF, Musselman KE, Livingstone D, Brunton K, Hendricks G, Hill D. et al. Repetitive mass practice or focused precise practice for retraining walking after incomplete spinal cord injury? A pilot randomized clinical trial. Neurorehabil Neural Repair 2014; 28: 314-324.
46. Dobkin B, Apple D, Barbeau H, Basso M, Behrman A, Deforge D, Ditunno J, Dudley G, Elashoff R, Fugate L, Harkema S, Saulino M, Scott M; Spinal Cord Injury Locomotor Trial Group. Weight-supported treadmill vs over-ground training for walking after acute incomplete SCI. Neurology. 2006 Feb 28;66(4):484-93.
47. Field-Fote Ec RKE. Influence of a locomotor training approach on walking speed and distance in people with chronic spinal cord injury: a randomized clinical trial. Physical therapy 2011; 91: 48.
48. Bi X, Lv H, Chen B-L, Li X, Wang X-Q. Effects of transcutaneous electrical nerve stimulation on pain in patients with spinal cord injury: a randomized controlled trial. Journal of Physical Therapy Science 2015; 27: 23-25.
49. Celik EC, Erhan B, Gunduz B, Lakse E. The effect of low-frequency TENS in the treatment of neuropathic pain in patients with spinal cord injury. Spinal cord 2013; 51: 334.
50. Cardenas DD, Felix ER, Cowan R, et al. Effects of Home Exercises on Shoulder Pain and Pathology in Chronic Spinal Cord Injury: A Randomized Controlled Trial. American journal of physical medicine & rehabilitation 2020; 99: 504-513.
51. Curtis KA, Tyner TM, Zachary L, et al. Effect of a standard exercise protocol on shoulder pain in long-term wheelchair users. Spinal cord 1999; 37: 421-429.

52. Dondal K, Kulkarni V, Patole R, et al. Effect of Shoulder Exercises on Functional Performance in Paraplegic Wheelchair users having Shoulder Pain. Indian Journal of Physiotherapy & Occupational Therapy 2015; 9: 83-86.
53. Mulroy SJ, Thompson L, Kemp B, et al. Strengthening and optimal movements for painful shoulders (STOMPS) in chronic spinal cord injury: a randomized controlled trial. Physical therapy 2011; 91: 305-324.
54. Nightingale TE, Rouse PC, Walhin JP, et al. Home-based exercise enhances health-related quality of life in persons with spinal cord injury: a randomized controlled trial. Archives of Physical Medicine and Rehabilitation 2018 Oct;99(10):1998-2006.
55. Crowe J, MacKay-Lyons M and Morris H. A multi-centre, randomized controlled trial of the effectiveness of positioning on quadriplegic shoulder pain. Physiotherapy Canada 2000 Fall;52(4):266-273.
56. Chase T, Jha A, Brooks CA, et al. A pilot feasibility study of massage to reduce pain in people with spinal cord injury during acute rehabilitation. Spinal Cord 2013 Nov;51(11):847-851.
57. Lovas J, Tran Y, Middleton J, Bartrop R, Moore N, Craig A. Managing pain and fatigue in people with spinal cord injury: A randomized controlled trial feasibility study examining the efficacy of massage therapy. Spinal Cord 2017; 55: 162-166.
58. Ben M, Harvey L, Denis S, et al. Does 12 weeks of regular standing prevent loss of ankle mobility and bone mineral density in people with recent spinal cord injuries? Australian journal of physiotherapy 2005;51:251.
59. Harvey LA, Batty J, Crosbie J, et al. A randomized trial assessing the effects of 4 weeks of daily stretching on ankle mobility in patients with spinal cord injuries. Arch Phys Med Rehabil 2000; 81:1340-1347.
60. Harvey LA, Byak AJ, Ostrovskaya M, et al. Randomised trial of the effects of four weeks of daily stretch on extensibility of hamstring muscles in people with spinal cord injuries. Aust J Physiotherapy 2003; 49:176-181.
61. Harvey L, Herbert R, Glinsky J, Moseley A and Bowden J. Effects of six months of regular passive movements on ankle joint mobility in people with spinal cord injury: A randomised controlled trial. Spinal Cord 2009. 47:62-68.
62. Chang Y-J, Liang J-N, Hsu M-J, et al. Effects of continuous passive motion on reversing the adapted spinal circuit in humans with chronic spinal cord injury. Archives of physical medicine and rehabilitation 2013; 94: 822-828.
63. Kwok, S., Harvey, L., Glinsky, J. et al. Does regular standing improve bowel function in people with spinal cord injury? A randomised crossover trial 2015. Spinal Cord 53, 36–41.
64. Ralston KE, Harvey LA, Batty J, et al. Functional electrical stimulation cycling has no clear effect on urine output, lower limb swelling, and spasticity in people with spinal cord injury: a randomised cross-over trial [with consumer summary]. Journal of Physiotherapy 2013 Dec;59(4):237-243
65. Hicks Al, Martin KA, Ditor DS, et al. Long-term exercise training in persons with spinal cord injury: effects on strength, arm ergometry performance and psychological well-being. Spinal cord 2003; 41: 34.

66. Yildirim A, Sürücü GD, Karamercan A, Gedik DE, Atci N, Dülgeroğlu D, Özgirgin N. Short-term effects of upper extremity circuit resistance training on muscle strength and functional independence in patients with paraplegia. Journal of Back and Musculoskeletal Rehabilitation. 2016 Nov 21;29(4):817-823.
67. Bye EA, Harvey LA, Gambhir A, et al. Strength training for partially paralysed muscles in people with recent spinal cord injury: a within-participant randomised controlled trial. Spinal Cord 2017 May;55(5):460-465.
68. Chen LW et al. effects of 10,000 voluntary contractions over 8 weeks on the strength of very weak muscles in people with spinal cord injury: a randomised controlled trial. Spinal cord 2020.
69. Glinsky J, Harvey L, Korten M, et al. Short-term progressive resistance exercise may not be effective at increasing wrist strength in people with tetraplegia: a randomised controlled trial. Australian Journal of Physiotherapy 2008;54(2):103-108 2008.
70. Demchak TJ, Linderman JK, Mysiw WJ, Jackson R, Suun J, Devor ST. Effects of functional electric stimulation cycle ergometry training on lower limb musculature in acute sci individuals. J Sport Sci Med 2005;4(3):263–71.
71. Baldi JC, Jackson RD, Moraille R and Mysiw WJ. Muscle atrophy is prevented in patients with acute spinal cord injury using functional electrical stimulation. Spinal cord 1998; 36: 463.
72. Glinsky J, Harvey L, van Es P, et al. The addition of electrical stimulation to progressive resistance training does not enhance the wrist strength of people with tetraplegia: a randomized controlled trial. Clinical rehabilitation 2009; 23: 696-704.
73. Harvey LA, Fornusek C, Bowden JL, et al. Electrical stimulation plus progressive resistance training for leg strength in spinal cord injury: a randomized controlled trial. Spinal Cord 2010 Jul;48(7):570-575 2010.
74. Bosveld R, Field-Fote EC. Single-dose effects of whole body vibration on quadriceps strength in individuals with motor-incomplete spinal cord injury. J Spinal Cord Med. 2015 Nov;38(6):784-91.
75. Akkurt H, Karapolat HU, Kirazli Y, Kose T. The effects of upper extremity aerobic exercise in patients with spinal cord injury: a randomized controlled study. Eur J Phys Rehabil Med. 2017 Apr;53(2):219-227.
76. Taylor AW, McDonell E, Brassard L. The effects of an arm ergometer training programme on wheelchair subjects. Paraplegia 1986. 24:105-114.
77. Kim D-I, Lee H, Lee B-S, et al. Effects of a 6-Week Indoor Hand-Bike Exercise Program on Health and Fitness Levels in People with Spinal Cord Injury: A Randomized Controlled Trial Study. Archives of physical medicine and rehabilitation 2015; 96: 2033-2040.e2031.
78. Bombardier CH, Dyer JR, Burns P, et al. A tele-health intervention to increase physical fitness in people with spinal cord injury and cardiometabolic disease or risk factors: a pilot randomized controlled trial. Spinal cord 2020.
79. Kim DI, Taylor JA, Tan CO, Park H, Kim JY, Park SY, Chung KM, Lee YH, Lee BS, Jeon JY. A pilot randomized controlled trial of 6-week combined

exercise program on fasting insulin and fitness levels in individuals with spinal cord injury. Eur Spine J. 2019 May;28(5):1082-1091.

80. Ma, J. K. West, C. R. Martin Ginis, K. A. The effects of a patient and provider co-developed, behavioral physical activity intervention on physical activity, psychosocial predictors, and fitness in individuals with spinal cord injury: a randomized controlled trial. Sports Medicine 2019 Jul;49(7):1117-1131.
81. Van der Scheer JW, de Groot S, Tepper M, Faber W; ALLRISC group, Veeger DH, van der Woude LH. Low-intensity wheelchair training in inactive people with long-term spinal cord injury: A randomized controlled trial on fitness, wheelchair skill performance and physical activity levels. J Rehabil Med. 2016 Jan;48(1):33-42.
82. National Institute for Health and Care Excellence. Developing Nice Guidelines the Manual. 31 October 2014. https://www.nice.org.uk/process/pmg20/chapter/appendices Last accessed 24/9/22.
